# Borazatruxenes†
†Electronic supplementary information (ESI) available: Detailed synthetic protocols, modelling and analytical data. CCDC 1843607, 1843608 and 1865474. For ESI and crystallographic data in CIF or other electronic format see DOI: 10.1039/c9sc02489a


**DOI:** 10.1039/c9sc02489a

**Published:** 2019-08-28

**Authors:** Simone Limberti, Liam Emmett, Anamaria Trandafir, Gabriele Kociok-Köhn, G. Dan Pantoş

**Affiliations:** a Department of Chemistry , University of Bath , Bath , BA2 7AY , UK . Email: g.d.pantos@bath.ac.uk

## Abstract

We report the synthesis and characterization of a series of arene-borazine hybrids called borazatruxenes. These molecules are BN-isosteres of truxene whereby the central benzene core has been replaced by a borazine ring. The straightforward three step synthesis, stability and their chiroptical and electronic properties recommend them as new scaffolds for BN–carbon hybrid materials. Computational studies at DFT level, closely matching the experimental data, provided insights in the electronic structure of these molecules.

## Abstract 2

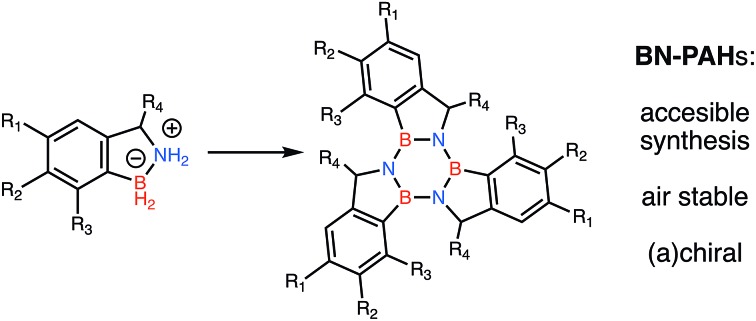
We report the straight forward synthesis of a series of arene-borazine hybrids (BN-PAHs) called borazatruxenes; the DFT, solid state and solution characterisation are reported along with the separation and chiroptical studies of four optical isomers.

## Introduction

The interest in polycyclic aromatic hydrocarbons (PAHs) has grown over the past decade due to their stability, tuneable properties and versatility.^1^–^3^ This has made them ideal candidates for use as semiconductors in the next generation of organic electronic components.^4^,^5^ Moreover, the electronic properties of PAHs are intimately related to their structure; therefore small modifications in their scaffold can lead to vastly different optical and electronic properties.^6^–^14^

Truxene is a C_3_-symmetric PAH that has been intensively studied for the past 15–20 years.^15^ In particular, it has been used as a precursor to polyarenes,^16^,^17^ liquid crystals^18^,^19^ and hemi-fullerenes,^20^,^21^ all of which have potential to be used in organic electronics.^22^ Many truxene derivatives have been synthesised^23^–^25^ and utilized for their light emitting and light harvesting properties^26^–^28^ while other analogues have found applications in dendrimers,^29^–^31^ gels,^32^,^33^ photoresists,^34^,^35^ fluorescent probes,^36^,^37^ and two photon absorbers.^38^,^39^

The B–N bond is quasi-isosteric and isoelectronic with the C=C bond,^40^,^41^ except that it is more polarized^42^ due to the difference in electronegativity between the B and N atoms. Therefore, replacing a C=C with a B–N in a PAH will lead to quasi-identical geometries but drastically different electronic structures.^43^ This concept has been at the core of the development of BN doped graphenes and BN-PAHs. Borazine containing PAHs are prone to hydrolytic decomposition which delayed the development of this field.^43^ In most cases, this reactivity is mitigated by steric bulk,^44^,^45^ introduction of electron rich substituents or structural confinement of the borazine ring.^45^–^52^

## Results and discussion

**Scheme 1:**
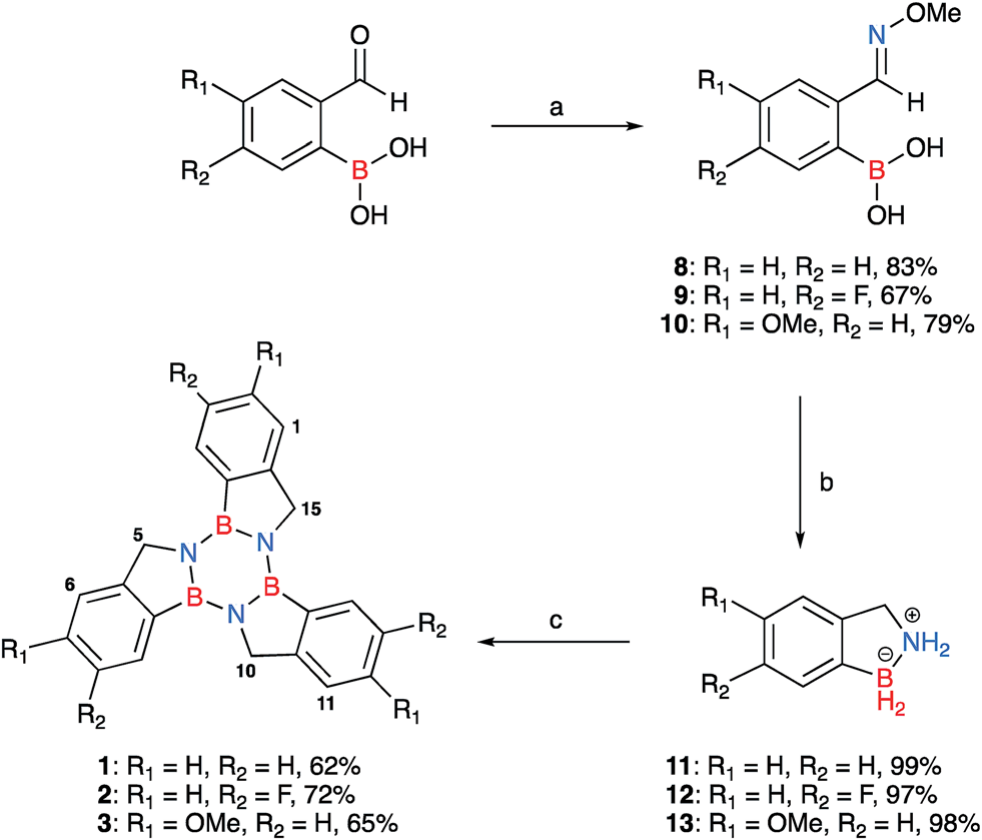
Synthesis of borazatruxenes **1–3** from substituted 2-formylphenylboronic acids; the isolated yields for each derivative are given after their descriptor. (a) (i) H_2_O, 45 °C, 15 min, (ii) NH_2_OMe·HCl (1.3 equiv.), 10% NaOH (w/w%) pH 7, (iii) reflux, 15 min; (b) LiAlH_4_ (3 equiv.) THF, –78 °C, then room temperature, followed by heating to reflux, 3 h; (c) PhMe, μW irradiation, 2 h, 180 °C. The numbering scheme adopted for the borazatruxenes is also shown for compounds **1–3**.

Herein we introduce a new class of borazine-PAHs that are moisture stable§
§The borazatruxenes are stable in solid state for at least one year if kept under N_2_ in close capped vials; they are stable in solution for at least two weeks with minimal decomposition. and easy to synthesise. Borazatruxenes are truxene analogues in which the central benzene core has been replaced by borazine. The B atom is directly linked to the phenylene rings which are connected *via* a methylene bridge to the N atom of the borazine core. This particular arrangement is responsible for the borazatruxenes' hydrolytic stability. Borazatruxenes **1–3** were synthesised according to the synthetic pathway illustrated in Scheme 1.

Commercially available *meta*- and *para*- substituted 2-formylbenzeneboronic acids were reacted with methoxyamine hydrochloride under refluxing conditions for 15 minutes in water (pH 7) to produce compounds **8–10** in high yields (70–90%). LiAlH_4_ in THF was added dropwise to a solution of phenylboronic acid derivatives **8–10** in THF at –78 °C, the mixture was allowed to warm to room temperature followed by heating to reflux for 3 hours. In all cases, the amine-borane^53^ product **11–13**, a BN analogue of indane, was isolated in excellent yields (85–90%). Trimerisation^54^ of **11–13** under microwave-assisted conditions afforded the desired borazatruxenes **1–3** in 56–65% yields as white solids after a simple filtration/washing protocol.

**Scheme 2:**
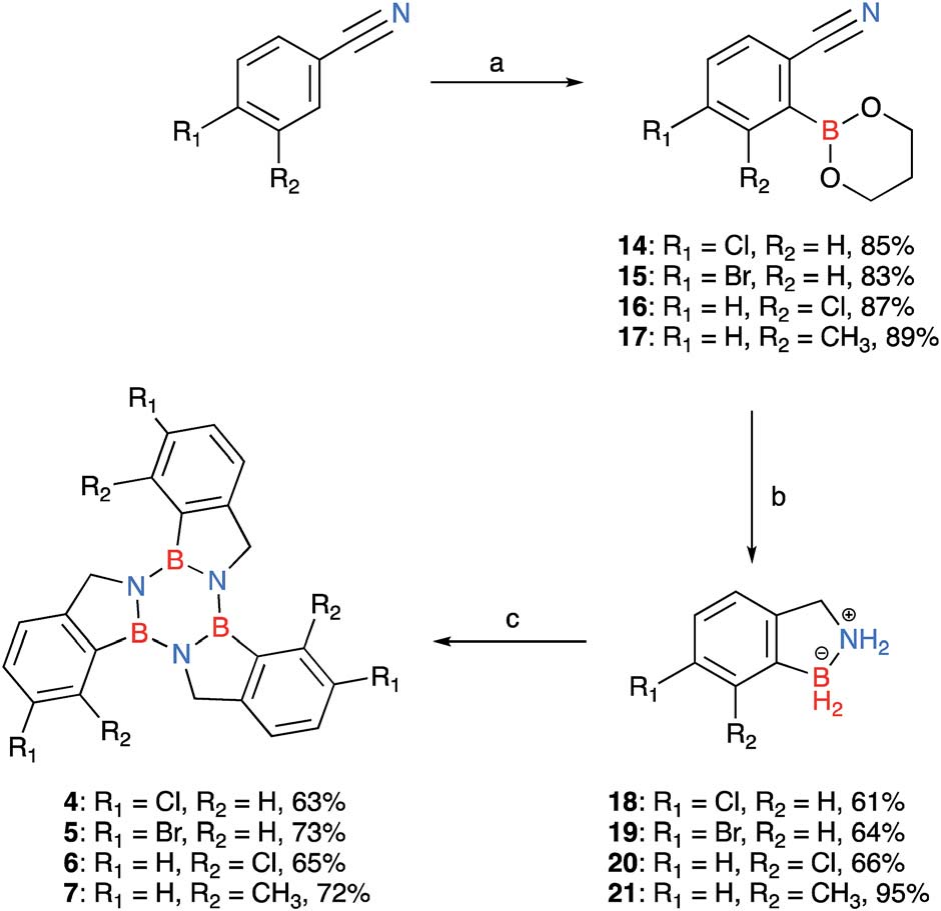
Synthesis of borazatruxenes **4–7** from substituted benzonitriles; the isolated yields for each derivative are given after their descriptor. (a) (i) LTMP (*in situ*: *n*-BuLi 2.5 M in *n*-hexane (1.5 equiv.) and TMP (1.5 equiv.)), THF, –10 °C, (ii) B(O^i^Pr)_3_ (2.0 equiv.), –78 °C, benzonitrile (1.0 equiv.) then room temperature, 16 h, (iii) AcOH (2.2 equiv.), 1,3-propanediol (6.0 equiv.); (b) LiAlH_4_ (5.0 equiv.) THF, –78 °C, then room temperature, followed by μW irradiation, 1.5 h, 90 °C; (c) PhMe, μW irradiation, 2 h, 180 °C.

**Scheme 3:**
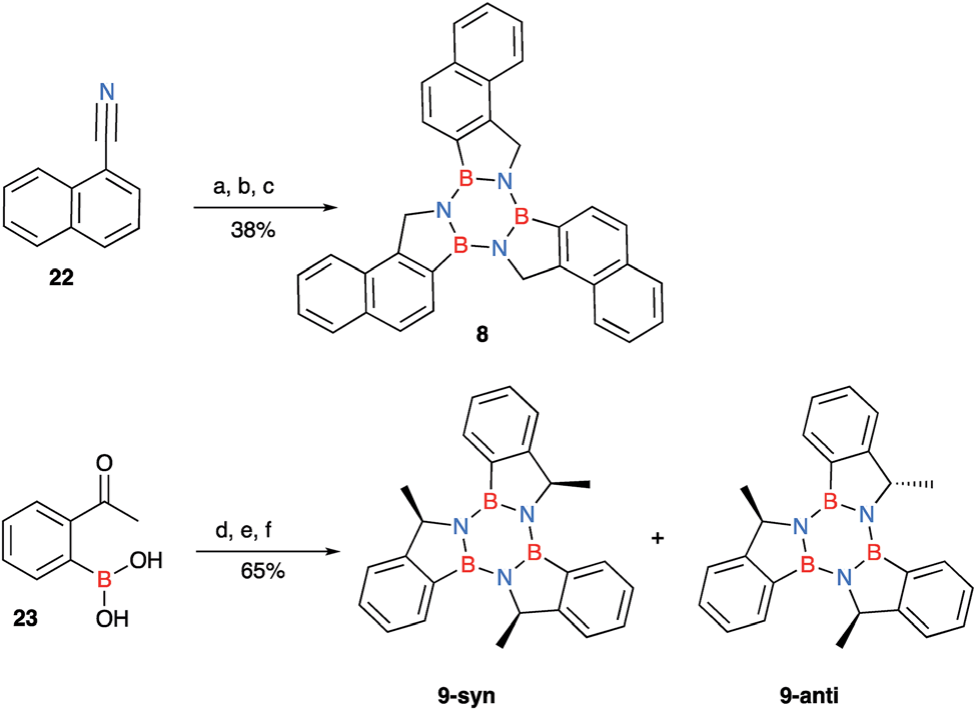
Synthesis of naphthylborazatruxene **8** from 1-naphthonitrile and the chiral trimethylborazatruxene **9**, the reported yields are over three steps. (a) (i) LTMP (*in situ*: *n*-BuLi 2.5 M in *n*-hexane (1.5 equiv.) and TMP (1.5 equiv.)), THF, –10 °C, (ii) B(O^i^Pr)_3_ (2.0 equiv.), –78 °C, benzonitrile (1.0 equiv.), then room temperature, o.n, (iii) AcOH (2.2 equiv.), 1,3-propanediol (6.0 equiv.); (b) LiAlH_4_ (3.0 equiv.) THF, –78 °C, then room temperature, followed by reflux, 48 h; (c) PhMe, μW irradiation, 2 h, 180 °C; (d) NH_2_OMe·HCl (1.2 equiv.), MeOH, 36 °C, 16 h; (e) LiAlH_4_ (5.0 equiv.) THF, –78 °C, then room temperature, followed by μW irradiation, 1.5 h, 90 °C; (f) PhMe, μW irradiation, 2 h, 180 °C.

Due to the limited number of commercially available 2-formylphenylboronic acids and their relatively lengthy syntheses, a second pathway was devised in order to obtain functionalised borazatruxenes (Scheme 2). 2-Cyanobenzene-boronic esters **14–17** were synthesised from *meta*- and *para*- substituted benzonitriles. The benzonitriles were reacted with lithium tetramethylpiperidine (LTMP) and B(O^i^Pr)_3_ at –78 °C, followed by slow warming to room temperature (20 °C) overnight to afford the respective *ortho*- substituted 2-cyanophenylboronic acid. The reaction was quenched using AcOH (2.2 equiv.) followed by an *in situ* protection of the boronic acid with 1,3-propanediol. The use of a protecting group is key for isolation of **14–17**, and the choice of 1,3-propanediol allows the reduction to the corresponding amine-borane products **18–21** in very good yields (65–95%), without requiring a further deprotection step (Scheme 2 and ESI†). Using more robust protecting groups, such as pinacol or neopentyl glycol, results in the reduction of the CN group to the corresponding 1° amine while the protected boronic acid remains intact under the reduction conditions utilised. Trimerisation of amine-boranes **18–21**, using microwave assisted synthesis in dry toluene at 180 °C for 2 hours, provided borazatruxenes **4–7** in 50–70% yields. Two additional borazatruxenes, **8** and **9** (Scheme 3), have been synthesised in order to expand the scope of the reaction to larger PAHs and chiral derivatives (*vide infra*), respectively.

Borazatruxene **1** is soluble in organic solvents of medium polarity. This is in stark contrast to the parent truxene, which has very poor solubility in most common solvents.^15^ The halogenated borazatruxene derivatives have significantly lower solubility compared to **1**, which is likely due to their increased molecular weight. The ^1^H-NMR of **1** (CDCl_3_, 300 MHz) indicates that the aromatic protons closest to the borazine rings are most deshielded (8.03–8.05 ppm) while the external ones experience a lower influence of the BN anisotropy with chemical shifts in the range of 7.42–7.59 ppm. The ^11^B NMR spectrum (CDCl_3_, 96 MHz) displays a peak resonating at 37.2 ppm which is in good agreement with a typical ^11^B chemical shift of a substituted borazine. The UV-vis spectrum of **1** shows a very intense and broad absorbance centered at 250 nm followed by three distinct peaks of lower intensity at 279.5, 272.0 and 265.0 nm. These peaks are blue shifted compared to corresponding truxene ones, thus highlighting that introducing BN bonds into the all-carbon system increases the HOMO–LUMO gap (Fig. 3). The borazatruxenes have higher quantum yields than the corresponding all-carbon derivatives. Borazatruxenes **5** and **8** aggregate at concentrations higher than 0.05 mM, while compounds **3**, **5** and **6** are non-emissive. The variable temperature UV-vis and emission spectra, as well as the excitation spectra for the molecules that exhibited emission, are collated in the ESI, Fig. S10–S18.† The extinction coefficients, quantum yields and the excitation and emission wavelengths are collated in Table 1.

**Table 1:** Collated UV-vis and fluorescence spectroscopic data of borazatruxenes **1–9**^*a*^

Borazatruxene	*λ*_ex_ (nm)	*ε* (L mol^–1^ cm^–1^)	*λ*_em_ (nm)	*Φ* ^*b*^ (A)
**1**	279.5	10 650	294	0.065
**2**	285.5	10 280	301	0.104
**3**	287.5	12 950	380	–^*c*^
**4**	290	6740	305	0.099
**5**	290	2880	294	–^*c*^
**6**	289	4410	298	–^*c*^
**7**	278	6563	301	0.039
**8**	298.5	7930	304	–^*c*^
**9**	272	3177	293	0.038^*d*^

^*a*^HPLC grade CHCl_3_.

^*b*^Determined using anthracene standard.

^*c*^Not determined due to aggregation.

^*d*^The solvent was *n*-hexane : 2-propanol 9 : 1.

**Fig. 1:**
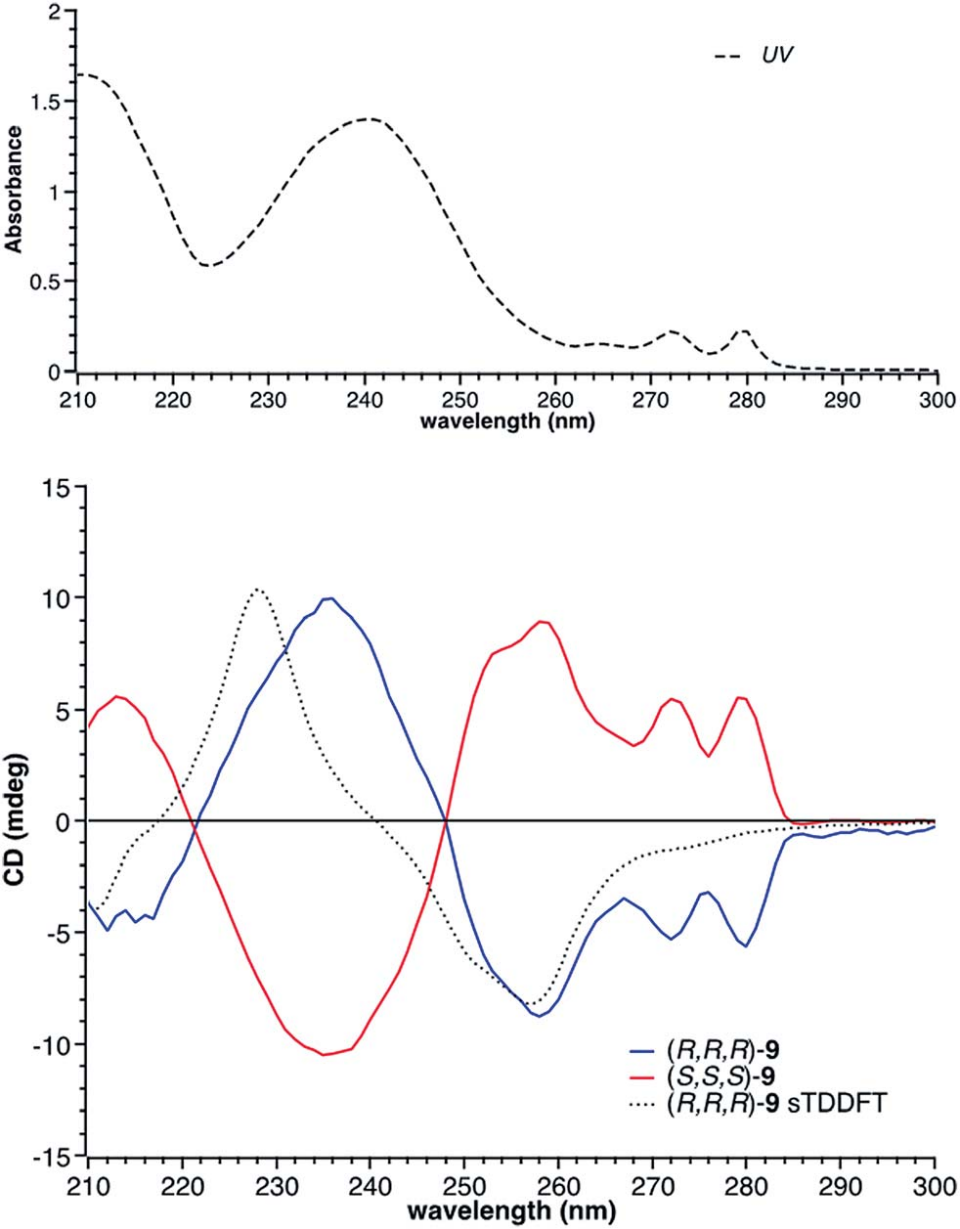
Top: UV spectrum of (*R*,*R*,*R*)-**9** (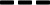); bottom: the CD spectra of the two enantiomers of the *syn* isomer of **9**, along with the computed CD spectrum (sTDDFT) of the (*R*,*R*,*R*)-**9**.

There are four possible optical isomers of borazatruxene **9** despite having three stereogenic centres. This is because the molecule has C_3_ symmetry which makes the (*R*,*R*,*S*) isomer identical with the (*R*,*S*,*R*) and (*S*,*R*,*R*) isomers. Therefore, the only possible isomers are the enantiomeric pairs *syn*: (*R*,*R*,*R*) and (*S*,*S*,*S*) with all three methyl groups located on the same side of the borazatruxenes plane, and *anti*: (*R*,*R*,*S*) and (*S*,*S*,*R*), where one methyl is on the opposite side of the plane with respect to the other two (Scheme 3). The *syn* and *anti* enantiomeric pairs can be readily separated because the *syn* isomer is more soluble in an 8 : 2 *n*-hexane : CH_2_Cl_2_ mixture and thus it can be isolated through recrystallisation. Furthermore, all four stereoisomers can be separated using a Chiralpak OD chiral column (details in the ESI†). The selectivity factor for the *syn* enantiomers is 1.92 (98 : 2 *n*-hexane : propan-2-ol, 0.5 ml min^–1^) while the *anti* enantiomers are separated with a selectivity factor of 1.17 (99 : 1 *n*-hexane : propan-2-ol, 0.5 ml min^–1^). The Circular Dichroism (CD) spectra of the two enantiomeric pairs *syn* (*R*,*R*,*R* and *S*,*S*,*S*) and *anti* (*R*,*R*,*S* and *R*,*S*,*S*) of chiral derivative **9** display strong Cotton effects on both the arene and borazine absorbance. The identification of all four isomers was possible by comparing the experimental (Fig. 1) with the computed (*vide infra*) CD spectra.

**Fig. 2:**
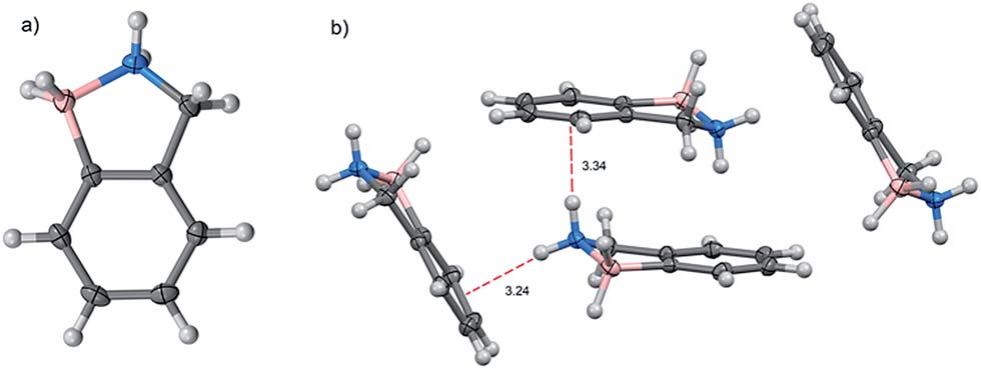
(a) Molecular structure and (b) unit cell of BN-indane **11**.

The molecular structure of BN-indane **11** (Fig. 2) was obtained from the X-ray analysis of single crystals grown by slow evaporation from an ethyl acetate solution. This compound crystallised in the centrosymmetric monoclinic space group *P*2_1/*n*_ with four molecule units per unit cell packed in staggered array motif.

The distance between boron and nitrogen atom is 1.637 Å consistent with an N → B Lewis-type interaction. There are two weak NH···π intermolecular interactions between the amine group with the aromatic rings of two neighbouring molecules: N···Ph(C2–C7) distance of 3.239 Å and N···Ph(C3, C4, C5) distance of 3.339 Å.

**Fig. 3:**
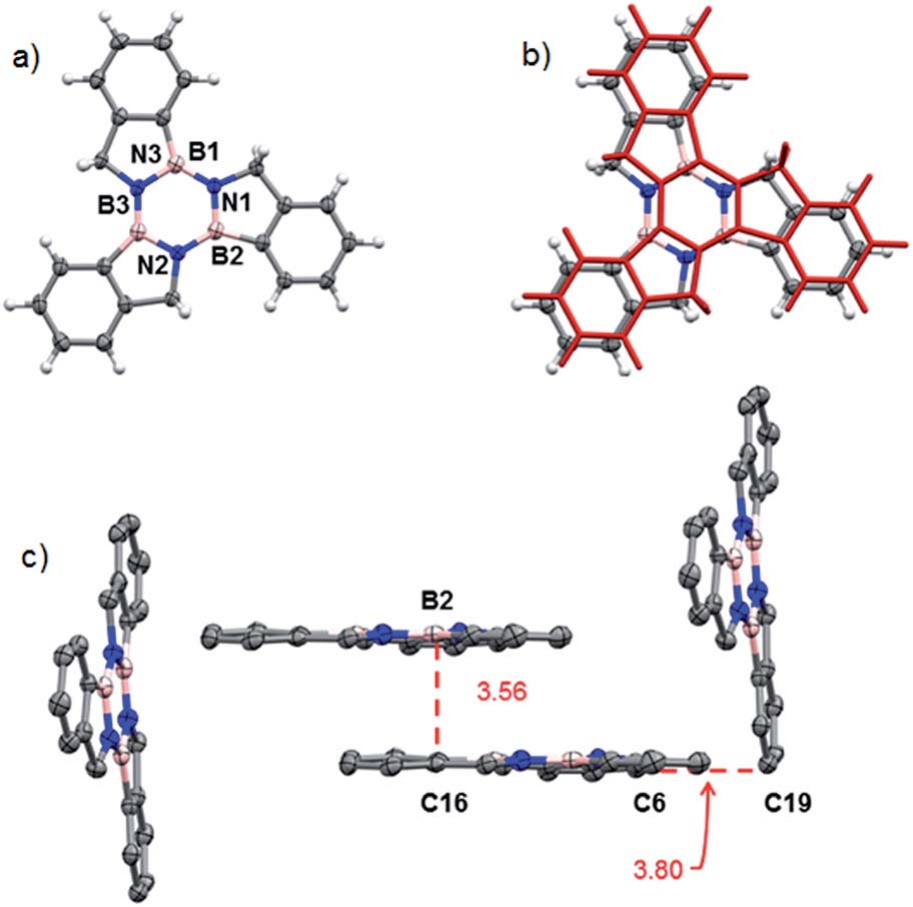
(a) Top view of the molecular structure of **1**;^44^ (b) the molecular structure of **1**, as determined by X-ray crystallography, overlaid (RMSD = 0.0603) with the computed structure for **1** in red, translated to (*x* + 1/2, *y*, *z* + 1/2) with respect to the X-ray coordinates; (c) packing structure highlighting the CH···π and aromatic π···π interactions.

Structural proof of borazatruxene **1** came from an X-ray diffraction analysis of single crystals grown by slow evaporation from a CH_2_Cl_2_ solution. Fig. 3A shows a top view of the X-ray structure of **1**, which crystallises in a *P*2_1_/*c* space group. This compound forms a staggered L-shaped configuration in the unit cell (Fig. 3C) where weak CH···π bonding is seen between C6 and C19 with the close contact distance being 3.801 Å. The π surfaces are also aligned parallel to one another with the closest distance between B2 and C16 being 3.561 Å.

**Fig. 4:**
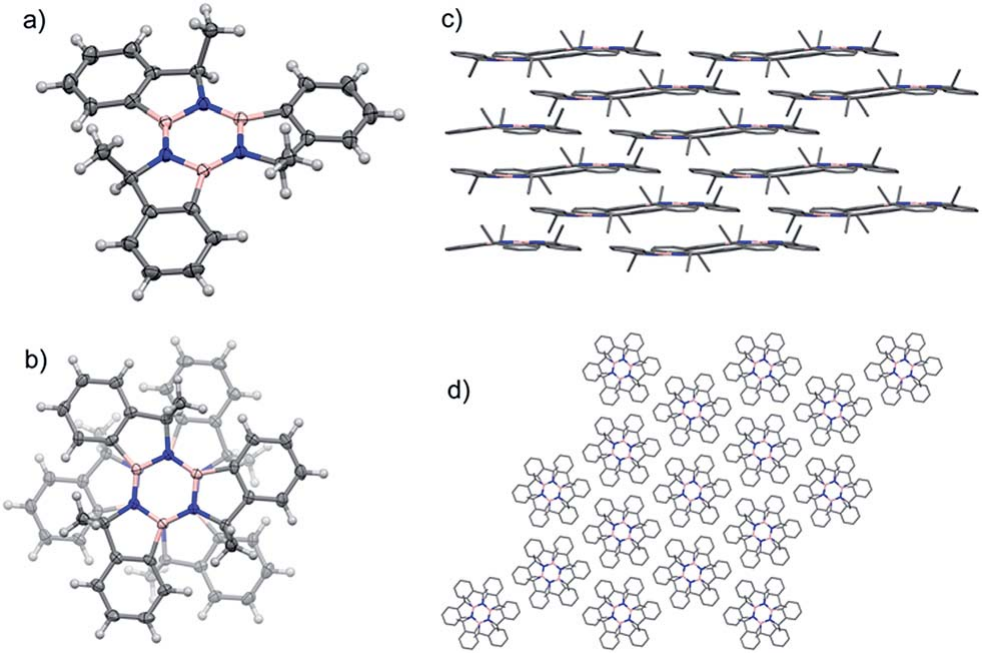
(a) Angled view of the molecular structure of *syn*-**9**;^44^ (b) stacking detail of the two enantiomers present in the structure. (c) Packing structure highlighting the layers and columns (d) present in the crystal structure.

Single crystals of the *syn* isomer of **9** were obtained from a 9 : 1 hexane : 2-propyl alcohol mixture. The X-ray diffraction revealed that the (*R*,*R*,*R*)-**9** and (*S*,*S*,*S*)-**9** co-crystallised in the *R*3[combining macron] space group. In this structure the two enantiomers adopt a 60° rotated face-on stacked arrangement with an intermolecular B–N distance of 3.749 Å and the Me groups pointing outwards (Fig. 4).

The molecules pack in an off-set columnar arrangement with a distance of 1.981 Å between two sequential planes of distinct columns.

The X-ray determined structure of **1** allowed us to validate a series of molecular modelling parameters used to predict the geometry of borazatruxenes **1–9**. Fig. 3b shows the translated (*x* + 1/2, *y*, *z* + 1/2) overlay of the experimentally determined (X-ray diffraction) co-ordinates and the computed ones. Geometry optimisations were performed using M06-2X,^55^ M11,^56^ M11-L^57^ or B3LYP^58^,^59^ exchange-correlation functionals and various basis sets as implemented in the *Gaussian 16* software. Also, TD-DFT^60^,^61^ (sTDDFT^62^) methods were employed to calculate the UV-vis and CD spectra with the above mentioned functionals (Fig. S8 and S21†), the best prediction for the CD spectra being obtained by M06-2X, while the closest agreement for the UV-vis spectra being given by the M11-L functional (Table S11†). Similarly the AICD properties (Fig. S22†) of borazatruxenes can be predicted at the M06-2X/6-311G level. A short study of the required geometry optimization time *versus* the agreement to experimental X-ray data (Table S10, Fig. S20†) yields M06-2X and M11 functionals along with 6-31G and 6-31G(d,p) basis sets as excellent choices for the modelling of borazatruxenes geometries while using reasonable computation resources.

## Conclusions

In summary, we have developed the synthesis of a new class of BN-PAHs that incorporate a borazine unit in place of the central benzene core of a truxene. These derivatives have higher solubilities than the parent all-carbon derivatives and can be synthesised in three steps from commercially available starting materials. These derivatives are air and moisture stable which is in contrast to the majority of non-sterically hindered borazines. We have synthesised and separated the first chiral derivatives of these molecules which also represents a premiere in the larger truxene family. The borazatruxenes molecules are likely to become important materials in molecular electronic devices due to their unique structure which combines areas of electron-conductance with electron-insulating domains. Further investigations into the synthesis of new borazatruxenes analogues are currently in progress in our group.

## Conflicts of interest

There are no conflicts to declare.

## Supplementary Material

Supplementary information

Crystal structure data

## References

[cit1] Tsefrikas V. M., Scott L. T. (2006). Chem. Rev..

[cit2] Feng X., Pisula W., Müllen K. (2009). Pure Appl. Chem..

[cit3] Dössel L., Gherghel L., Feng X., Müllen K. (2011). Angew. Chem., Int. Ed..

[cit4] Wu J., Pisula W., Müllen K. (2007). Chem. Rev..

[cit5] Chen F., Tao N. J. (2009). Acc. Chem. Res..

[cit6] Guo X., Baumgarten M., Müllen K. (2013). Prog. Polym. Sci..

[cit7] Wu J. and Müllen K., All-benzenoid Polycyclic Aromatic Hydrocarbons: Synthesis, Self-assembly and Applications in Organic Electronics, WILEY-VCH Verlag GmbH & Co. KGaA, 1st edn, 2006.

[cit8] Liu Z., Ishibashi J. S. A., Darrigan C., Dargelos A., Chrostowska A., Li B., Vasiliu M., Dixon D. A., Liu S.-Y. (2017). J. Am. Chem. Soc..

[cit9] Chrostowska A., Xu S., Mazière A., Boknevitz K., Li B., Abbey E. R., Dargelos A., Graciaa A., Liu S.-Y. (2014). J. Am. Chem. Soc..

[cit10] Ishibashi J. S. A., Marshall J. L., Mazière A., Lovinger G. J., Li B., Zakharov L. N., Dargelos A., Graciaa A., Chrostowska A., Liu S.-Y. (2014). J. Am. Chem. Soc..

[cit11] Tsuchiya S., Saito H., Nogi K., Yorimitsu H. (2019). Org. Lett..

[cit12] Zhang C., Zhang L., Sun C., Sun W., Liu X. (2019). Org. Lett..

[cit13] Müller M., Maichle-Mössmer C., Bettinger H. F. (2014). Angew. Chem., Int. Ed..

[cit14] Abengózar A., García-García P., Sucunza D., Sampedro D., Pérez-Redondo A., Vaquero J. J. (2019). Org. Lett..

[cit15] Goubard F., Dumur F. (2014). RSC Adv..

[cit16] Shi K., Wang J.-Y., Pei J. (2015). Chem. Rec..

[cit17] Boorum M. M. (2001). Science.

[cit18] Destrade C., Gasparoux H., Babeau A., Tinh N. H., Malthete J. (1981). Mol. Cryst. Liq. Cryst..

[cit19] Isoda K., Yasuda T., Kato T. (2009). Chem.–Asian J..

[cit20] Scott L. T. (2002). Science.

[cit21] Abdourazak A. H., Marcinow Z., Sygula A., Sygula R., Rabideau P. W. (1995). J. Am. Chem. Soc..

[cit22] Ruiz C., García-Frutos E. M., Hennrich G., Gómez-Lor B. (2012). J. Phys. Chem. Lett..

[cit23] de Frutos Ó., Gómez-Lor B., Granier T., Monge Á., Gutiérrez-Puebla E., Echavarren A. M. (1999). Angew. Chem., Int. Ed..

[cit24] Gómez-Lor B., de Frutos Ó., Ceballos P. A., Granier T., Echavarren A. M. (2001). Eur. J. Org. Chem..

[cit25] González-Cantalapiedra E., Ruiz M., Gómez-Lor B., Alonso B., García-Cuadrado D., Cárdenas D. J., Echavarren A. M. (2005). Eur. J. Org. Chem..

[cit26] Yang Z., Xu B., He J., Xue L., Guo Q., Xia H., Tian W. (2009). Org. Electron..

[cit27] Huang J., Xu B., Su J.-H., Chen C. H., Tian H. (2010). Tetrahedron.

[cit28] Omer K. M., Kanibolotsky A. L., Skabara P. J., Perepichka I. F., Bard A. J. (2007). J. Phys. Chem. B.

[cit29] Cao X.-Y., Zhang W.-B., Wang J.-L., Zhou X.-H., Lu H., Pei J. (2003). J. Am. Chem. Soc..

[cit30] Wang L., Jiang Y., Luo J., Zhou Y., Zhou J., Wang J., Pei J., Cao Y. (2009). Adv. Mater..

[cit31] Koenen J.-M., Jung S., Patra A., Helfer A., Scherf U. (2012). Adv. Mater..

[cit32] Tseng K.-P., Kao M.-T., Tsai T. W. T., Hsu C.-H., Chan J. C. C., Shyue J.-J., Sun S.-S., Wong K.-T. (2012). Chem. Commun..

[cit33] Gomez-Esteban S., Pezella M., Domingo A., Hennrich G., Gómez-Lor B. (2013). Chem.–Eur. J..

[cit34] Hattori S., Yamada A., Saito S., Asakawa K., Koshiba T., Nakasugi T. (2009). J. Photopolym. Sci. Technol..

[cit35] Guilhabert B., Laurand N., Herrnsdorf J., Chen Y., Mackintosh A. R., Kanibolotsky A. L., Gu E., Skabara P. J., Pethrick R. A., Dawson M. D. (2010). J. Opt..

[cit36] Haughey A.-M., Guilhabert B., Kanibolotsky A. L., Skabara P. J., Dawson M. D., Burley G. A., Laurand N. (2014). Biosens. Bioelectron..

[cit37] Yuan M. S., Wang Q., Wang W., Wang D. E., Wanga J., Wang J. (2014). Analyst.

[cit38] Tao C.-H., Yang H., Zhu N., Yam V. W.-W., Xu S.-J. (2008). Organometallics.

[cit39] Chan C. K. M., Tao C.-H., Tam H.-L., Zhu N., Yam V. W.-W., Cheah K.-W. (2009). Inorg. Chem..

[cit40] Müller M., Behnle S., Maichle-Mössmer C., Bettinger H. F. (2014). Chem. Commun..

[cit41] Liu Z., Marder T. B. (2008). Angew.Angew.
Chem., Int. Ed.Chem., Int. Ed..

[cit42] Côté M., Haynes P., Molteni C. (2001). Phys. Rev. B.

[cit43] Bonifazi D., Fasano F., Lorenzo-Garcia M. M., Marinelli D., Oubaha H., Tasseroul J. (2015). Chem. Commun..

[cit44] Kervyn S., Kalashnyk N., Riello M., Moreton B., Tasseroul J., Wouters J., Jones T. S., De Vita A., Costantini G., Bonifazi D. (2013). Angew. Chem., Int. Ed..

[cit45] Marinelli D., Fasano F., Najjari B., Demitri N., Bonifazi D. (2017). J. Am. Chem. Soc..

[cit46] Biswas S., Müller M., Tönshoff C., Eichele K., Maichle-Mössmer C., Ruff A., Speiser B., Bettinger H. F. (2012). Eur. J. Org. Chem..

[cit47] Müller M., Maichle-Mössmer C., Sirsch P., Bettinger H. F. (2013). ChemPlusChem.

[cit48] Ciccullo F., Calzolari A., Píš I., Savu S.-A., Krieg M., Bettinger H. F., Magnano E., Chassé T., Casu M. B. (2016). J. Phys. Chem. C.

[cit49] Snyder J. A., Grüninger P., Bettinger H. F., Bragg A. E. (2017). J. Phys. Chem. A.

[cit50] Dosso J., Tasseroul J., Fasano F., Marinelli D., Biot N., Fermi A., Bonifazi D. (2017). Angew. Chem., Int. Ed..

[cit51] Krieg M., Reicherter F., Haiss P., Ströbele M., Eichele K., Treanor M.-J., Schaub R., Bettinger H. F. (2015). Angew. Chem., Int. Ed..

[cit52] Sánchez-Sánchez C., Brüller S., Sachdev H., Müllen K., Krieg M., Bettinger H. F., Nicolaï A., Meunier V., Talirz L., Fasel R., Ruffieux P. (2015). ACS Nano.

[cit53] Beguerie M., Faradji C., Vendier L., Sabo-Etienne S., Alcaraz G. (2017). ChemCatChem.

[cit54] Luo W., Campbell P. G., Zakharov L. N., Liu S.-Y. (2011). J. Am. Chem. Soc..

[cit55] Zhao Y., Truhlar D. G. (2008). Theor. Chem. Acc..

[cit56] Peverati R., Truhlar D. G. (2011). J. Phys. Chem. Lett..

[cit57] Peverati R., Truhlar D. G. (2012). J. Phys. Chem. Lett..

[cit58] Becke A. D. (1993). J. Chem. Phys..

[cit59] Lee C., Yang W., Parr R. G. (1988). Phys. Rev. B: Condens. Matter Mater. Phys..

[cit60] Hohenberg P., Kohn W. (1964). Phys. Rev..

[cit61] Runge E., Gross E. K. U. (1984). Phys. Rev. Lett..

[cit62] Bannwarth C., Grimme S. (2014). Comput. Theor. Chem..

