# Supplementary material for: Borazatruxenes
Source: Chem Sci. 2019 Aug 28;10(41):9565–70. doi: 10.1039/c9sc02489a (PMC6979502; doi:10.1039/c9sc02489a)
Supplement: Supplementary file 1 [file SC-010-C9SC02489A-s001.pdf]

Electronic Supplementary Material (ESI) for Chemical Science.
This journal is © The Royal Society of Chemistry 2019

Supplementary Information for:

Borazatruxenes

Simone Limberti, Liam Emmett, Anamaria Trandafir, Gabriele Kociok-Köhn and Dr G. Dan Pantoş*

Department of Chemistry, University of Bath, Bath, BA2 7AY, UK

E-mail: g.d.pantos@bath.ac.uk

Contents:

GENERAL EXPERIMENTAL 2

SYNTHETIC DETAILS 3

HPLC DATA FOR COMPOUND 9 20

CD DATA FOR COMPOUND 9 24

UV-VIS, EMISSION AND EXCITATION SPECTRA 27

X-RAY DATA FOR COMPOUNDS 11, 1 AND 9-*SYN* 35

MOLECULAR MODELLING DATA 41

NMR SPECTRA 46

INFRARED DATA FOR BORAZATRUXENES 50

REFERENCES 51

General Experimental

All reactions were carried out using anhydrous solvents and kept under an inert atmosphere of nitrogen as specified. Solvents were obtained by passing through anhydrous alumina columns using Innovative Technology Inc. PS-400-7 solvent purification system. All reagents were purchased from commercial suppliers: Acros Organics, Alfa Aesar, Sigma Aldrich, TCI Europe, Gross, Fluorochem or Apollo Scientific and used without further purification.

^{1}H, ^{11}B and ^{13}C were performed on Bruker Advance 300 (^{1}H 300 MHz, ^{11}B 96 MHz ^{13}C 75 MHz), Bruker Advance 400 (^{1}H 400 MHz, ^{11}B 128 MHz and ^{13}C 100 MHz) and Bruker Advance 500 (^{1}H 500 MHz, ^{11}B 160 MHz and ^{13}C 125 MHz) as stated. Chemical shifts are reported in parts per million (ppm) relative to tetramethyl silane (δ = 0.00) and $BF_3{\cdot}Et_2O$ (δ = 0.00) for ^{1}H NMR and ^{11}B NMR respectively. Coupling constants are reported in Hertz (Hz) and signal multiplicity is denoted as singlet (s), doublet (d), doublet of doublet (dd), quartet (q), multiplet (m) and broad (b). All spectra were acquired at the specified temperatures.

Mass spectrometry (MS) was performed using either a Finnigan MAT 95 XP high resolution double focussing (BE) mass spectrometer in EI mode performed by the EPSRC National Mass Spectrometry Facility at Swansea, UK or a Finnigan LCQ Classic mass spectrometer in ESI mode. The microwave reactions were carried out in either CEM Discover, CEM Explorer 12, or Biotage Initiator dedicated microwave reactors. HPLC analyses were carried out on a Perkin Elmer 200series instrument equipped with a diode array detector and a Jasco OR-1590 chiral detector controlled via a PE Nelson 950 interface. The UV-vis, CD, Emission and Excitation spectra were acquired on an Applied Photophysics Chirascan spectrophotometer equipped with a SEM and a Peltier-controlled sample holder. This research made use of the Balena High Performance Computing (HPC) Service at the University of Bath.

The common solvent impurities in ^{1}H and ^{13}C NMR in very small amounts were water, grease, benzene and chlorobenzene. The chemical shift of the above impurities in *d*-chloroform and *d*-1,1,2,2-tetrachloroethane are as follows.

	chloroform		*d*-1,1,2,2-tetrachloroethane	
	^{1}H	^{13}C	^{1}H	^{13}C
Water	1.56 (s)	-	1.61 (s)	-
Grease*	0.86 (s), 1.26 (s)	29.8	1.26 (s), 0.89 (s)	29.7
Benzene	7.36 (s)	128.4	7.38 (s)	127.8
Chlorobenzene	7.43 - 7.14 (m)	134.3, 129.7, 128.6, 126.4	7.38 – 7.29 (m)	-

Synthetic details

General Procedure A: synthesis of (2-((methoxyimino)methyl)phenyl)boronic acid derivatives.[1]

2-Formylboronic acid was charged into a 25 mL RBF alongside a stir bar. Deionised water was added to the flask and the reactants were heated to 45 °C for 15 minutes to hydrolyse any boroxine impurities. At RT, methoxylamine hydrochloride was added portion-wise, resulting in the formation of a distinct white precipitate. The suspension was neutralised (pH 7) by addition of NaOH solution (10% v/v). The reactants were refluxed for 15 minutes. During cooling, the stir bar was removed and the flask replaced on the warm heating mantle and cooled slowly, allowing for slow crystallisation of the product. At RT the flask was placed in a fridge to further crystallise overnight. Whether crystals or precipitate, the product was filtered, washed with fresh deionised water and placed under vacuum (1 mbar).

General Procedure B: Lithiation-borylation of benzonitrile derivatives.[2]

1,1,2,2-Tetramethylpyrollidine was charged into a flame dried 2-neck 100 mL RBF alongside a stir bar under N_2. THF was syringed into the flask to give a homogenous solution. The flask was cooled to 0 °C when n-butyl lithium solution (2.5 M in hexane) was added dropwise to produce a yellow solution. The flask was cooled to -78 °C where triisopropylborate was added slowly and stirred for 10 minutes. A premade solution of benzonitrile derivative in THF was added dropwise to the flask, where a distinct colour change was seen. The flask was left to stir overnight, allowing the reaction mixture to slowly warm to RT. AcOH was added to quench the reaction, stirring for 30 minutes. 1,3-propanediol was added *via* syringe which was left to mix for a further 30 minutes. The reaction was extracted with CH_2Cl_2, washing the organic layer with $KHPO_4$ (10% v/v). The aqueous layer was back extracted with CH_2Cl_2 and the organic fractions combined. The organic layer was dried over $MgSO_4$, filtered and concentrated. The resulting residue was re-dissolved in CH_2Cl_2 and an extra 4 equivalence 1,3-propanediol was added and stirred for 1 hour. The organic layer was washed with water. The aqueous layer was back extracted with CH_2Cl_2, combining the organic layers. The organic layer was dried over $MgSO_4$, filtered and concentrated.

General Procedure C: Reduction of (2-((Methoxyamino)methyl)benzene boronic acid or 2-cyanobenzeneboronate ester derivatives to amine-boranes.[3]

(2-((Methoxyamino)methyl)benzene boronic acid or 2-cyanobenzeneboronate ester derivative was charged into a flame dried 2-neck 100 mL RBF alongside a stir bar under N_2. THF was syringed into the flask, dissolving the starting material. The flask was cooled to -78 °C where $LiAlH_4$ (1.0 mol in THF) was added dropwise over 5 minutes. The reactants were allowed to mix for 10 minutes before removing the dry ice/acetone bath and allowing the flask to warm to RT. The reaction was refluxed for 3 hours. Once cooled, the flask was submerged in an ice bath before the reaction was quenched with water. The reaction mixture was filtered, washing with THF and concentrated. For purification, the resulting white solid was suspended in CH_2Cl_2 and washed with

1M HCl (2 x 30 mL) and brine (1 x 30 mL) where necessary. The organic layer was dried over $MgSO_4$, filtered and concentrated.

General Procedure D: Reduction of benzonitrile boronic ester and phenylacetonitrile boronic ester derivatives to amine-boranes by µW assisted conditions.

A 10 mL µW tube equipped with a stirring bar and sealed with a perforated plastic cap was flame-dried under reduced pressure though a needle. After back filling with argon, a solution of either benzonitrile boronic ester or phenylacetonitrile boronic ester derivative in anhydrous THF under argon atmosphere was cannulated and the system was cooled to -78°C. After 15 min, freshly titrated $LiAlH_4$ (≈1.0 M in THF) (5.0 equiv.) was added dropwise under vigorous stirring, and then the resulting mix was slowly allowed to warm to room temperature. The needle was removed to seal, and the µW tube was placed into the µW machine and the mixture was stirred and irradiated by microwave dielectric heating at 90 °C (power 40W). After 1 h, the reaction mixture was cooled to room temperature, diluted with anhydrous THF and transferred in a 100 mL conical flask. After cooling to -5 °C, the reaction was quenched dropwise with water under vigorous stirring yielding a white precipitate. The formed suspension was filtered on a plug of $MgSO_4$ and the solid was washed with THF (3 x 10 mL) and EtOAc (3 x 10 mL). After the organic solvents were removed under reduced pressure, the residue was re-dissolved in EtOAc (20 mL), transferred to a separating funnel and washed with distilled water (6 x 10 mL) and brine (1 x 10 mL). The combined water phase was back-extracted once with EtOAc (15 mL) while the combined organic phase was dried over $MgSO_4$, filtered and the solvent removed under reduced pressure to afford the purified desired.

General procedure E: Synthesis of borazatruxene derivatives.

A 10 mL µW tube equipped with a stirring bar and sealed with a perforated plastic cap was flame-dried under reduced pressure though a needle. After back filling with argon, a solution of amine-borane derivative in anhydrous toluene was cannulated and the suspension was stirred and irradiated by microwave dielectric heating at 180 °C (power 300W). After 3 h, the reaction mixture was cooled to room temperature, diluted with EtOAc and transferred in a 100 mL flask. After the organic solvents were removed under reduced pressure, the crude was washed with hexane (3 x 5 mL) and hexane:CH_2Cl_2 (50:50) (3 x 5 mL) and then dried under reduced pressure to afford the purified desired.

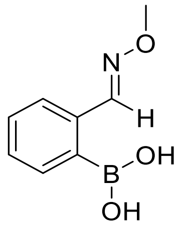

(2-((methoxyamino)methyl)benzene boronic acid, **8**.

Compound **8** was synthesised according to general procedure **A** using 2-formylbenzeneboronic acid (200 mg, 1.33 mmol), methoxylamine hydrochloride (151 mg, 1.81 mmol) in water (5 mL). After filtration and removal of residual water under vacuum, product was isolated as glassy crystals (83% yield).

^{1}**H NMR** (300 MHz, $CDCl_3$, 25 °C); 8.21 – 8.17 (^{3}J = 6.87, ^{4}J = 1.91, dd, 1H, *Ar*C**H**) 8.19 (s, 1H, C**H**) 7.51 – 7.36 (m, 3H, *Ar*C**H**) 4.02 (s, 3H, NOC**H$_3$**). ^{11}B (96 MHz, $CDCl_3$, 25 °C); 32.2. ^{13}C (125 MHz, $CDCl_3$. 25 °C); 153.21, 138.31, 135.0, 133.1, 130.9, 129.9, 62.2. **HRMS** calcd. for $C_8H_{10}BNO_3$ [M+H]+ (m/z): 180.0827, found: 180.0823. Melting Point 91 – 93 °C

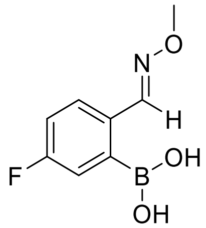

(5-Fluoro-(2-((methoxyamino)methyl))benzene boronic acid, **9**.

Compound **9** was synthesised according to general procedure **A** using 5-fluoro-2-formylbenzeneboronic acid (200 mg, 1.19 mmol), methoxylamine hydrochloride (135 mg, 1.62 mmol) in water (5 mL). Dissolution of starting material was not observed after 15 minutes of heating, yet precipitation after addition of methoxylamine hydrochloride was evident. The precipitate persisted after reflux and was isolated following filtration to give the desired product as a white solid (67 %).

^{1}**H NMR** (300 MHz, $CDCl_3$, 25 °C); 8.16 (s, 1H, C**H**), 7.92 – 7.87 (^{3}J = 10.03, ^{4}J = 2.80, dd, 1H, *Ar*C**H**), 7.37 – 7.32 (^{3}J = 8.5, ^{4}J = 5.4, dd, 1H, *Ar*C**H**), 7.20 – 7.12 (^{3}J = 11.81, ^{4}J = 2.88, td, 1H, *Ar*C**H**), 4.01 (s, 1H, 4.01). ^{11}**B NMR** (96 MHz, $CDCl_3$, 25 °C); 31.35. ^{13}**C NMR** (125 MHz, $CDCl_3$. 25 °C); 161.7, 135.4, 125.5, 125.2, 117.9, 117.6, 62.2. **HRMS** calc. for $C_8H_9BFNO_3$ [M+H]+ (m/z): 198.0732, found: 198.0729. Melting Point 117-119 °C

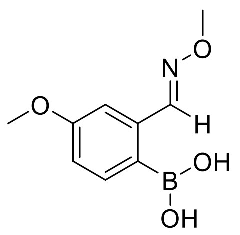

(4-Methoxy-(2-((methoxyamino)methyl))benzeneboronic acid, **10**.

Compound **10** was synthesised according to general procedure **A** using 4-methoxy-2-formylbenzeneboronic acid (200 mg, 1.11 mmol), methoxylamine hydrochloride (126 mg, 1.51 mmol) and water (5 mL). Dissolution of starting material was not observed after 15 minutes of heating, yet precipitation after addition of methoxylamine hydrochloride was evident. The precipitate persisted after reflux and was isolated to give the desired product as a white solid (79 %).

^{1}**H NMR** (300 MHz, $CDCl_3$, 25 °C); 8.17 - 8.14 (^{3}J = 7.63, d, 1H, s, *Ar*C**H**), 8.14 (s, 1H, C**H**) 6.98 - 6.94 (^{3}J = 8.44 Hz, ^{4}J = 2.55 Hz, dd, 1H, *Ar*C**H**), 6.9 – 6.89 (^{4}J = 2.52 Hz, d, 1H, *Ar*C**H**), 4.03 (S, 3H, NOC**H$_3$**) 3.85 (s, 1H, COC**H$_3$**). ^{11}**B NMR** (96 MHz, $CDCl_3$, 25 °C); 31.7. ^{13}C (125 MHz, $CDCl_3$. 25 °C); 161.6, 153.2, 140.5, 136.8, 119.7, 114.2, 62.3, 55.4. **HRMS** calcd. for $C_9H_{12}BNO_4$ [M+H]+ (m/z): 210.0932, found: 210.0932. Melting Point 163-165 °C

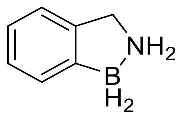

2,3-Dihydrobenzo[1,2]azaborole, **11**

Compound **11** was synthesised according to general procedure **C** reacting amine-borane (500 mg, 2.79 mmol) and 3 equiv. $LiAlH_4$ (8.38 mL, 8.38 mmol) in THF (30 mL) at -78 °C. The reaction mixture was quenched by adding water dropwise, very slowly. The reaction was warmed to room temperature and placed under reflux. The precipitate was filtered and washed with THF and the organic fraction concentrated to yield a white solid without further purification (99% yield).

^{1}**H NMR** (300 MHz, $CDCl_3$, 25 °C); 7.46 - 7.44 (^{3}J=7.12, 1H, d, *Ar*C**H**), 7.21 (1H, m, *Ar*C**H**), 7.21 (2H, m, *Ar*C**H**) 4.18 (^{3}J=11.65, 2H, t, C**H$_2$**NH_2), 4.03 (2H, b, CH_2N**H$_2$**). ^{11}**B NMR** (96 MHz, $CDCl_3$, 25 °C); -6.88. ^{13}**C NMR** (125 MHz, $CDCl_3$. 25 °C); 139.0, 135.8, 129.4, 127.3, 125.14, 121.5, 51.0. ^{1}**H NMR** (300 MHz, DMSO, 25 °C); 7.21 – 7.19 (^{3}J=6.97, 1H, d, *Ar*C**H**), 7.07 - 6.92 (3H, m, *Ar*C**H**), 5.93 (2H, b, CH_2N**H$_2$**), 3.95 (^{3}J=5.93, 2H, t, C**H$_2$**NH_2). ^{11}**B NMR** (96 MHz, DMSO, 25 °C); -8.56. ^{13}**C NMR** (125 MHz, DMSO, 25 °C); 141.1, 128.6, 125.8, 123.9, 121.0, 49.4. ^{1}**H NMR** (300 MHz, C_6D_6, 25 °C); 7.82 - 7.80 (^{3}J = 7.48, 1H, d, *Ar*C**H**,), 7.28 - 7.23 (^{3}J = 7.25, 1H, t, *Ar*C**H**), 7.14 - 7.09 (^{3}J = 7.52, 1H, t, *Ar*C**H**), 6.89 - 6.87 (^{3}J = 7.47, 1H, d, *Ar*C**H**), 3.02 - 2.98 (^{3}J = 3.00, 2H, t, C**H$_2$**NH_2), 2.25 (2H, b, CH_2N**H$_2$**). ^{11}**B NMR** (96 MHz, C_6D_6, 25 °C); -6.65. (1B, t). ^{13}**C NMR** (125 MHz, C_6D_6, 25 °C); 139.8, 130.5, 127.9, 126.3, 125.3, 121.6, 51.3. **HRMS** calc. for $C_7H_{10}BN$ [M+H]+ (m/z): 118.0821, found: 118.0823. Melting Point 87- 89 °C

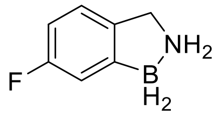

6-Fluoro-2,3-dihydrobenzo[1,2]azaborole, **12**

Compound **12** was synthesised according to general procedure **C** reacting amine-borane (500 mg, 2.54 mmol) and 3 equiv. $LiAlH_4$ (7.62 mL, 7.62 mmol) in THF (30 mL) at -78 °C. The reaction was warmed to room temperature and placed under reflux. The reaction mixture was quenched by adding water dropwise, very slowly. The precipitate was filtered and washed with THF and the organic fraction concentrated to yield a white solid (97% yield).

^{1}**H NMR** (300 MHz, DMSO, 25 °C); 7.09-7.05 (^3J = 8.15, ^4J= 5.09, 1H, dd, *Ar*C**H**,), 6.91-6.87 (^3J = 5.09, ^4J= 2.49 1H, dd, *Ar*C**H**), 6.76-6.69 (^3J = 8.87, ^4J = 2.99, 1H, td, *Ar*C**H**), 6.03. (2H, b, $CH_2N\mathbf{H_2}$) 3.93 (^3J = 5.94, 2H, t, $C\mathbf{H_2}NH_2$). ^{11}**B NMR** (96 MHz, DMSO, 25 °C); -9.75. ^{13}**C NMR** (125 MHz, DMSO, 25 °C); 139.2, 136.8, 124.9, 122.4,114.4, 110.6, 48.71. ^{1}**H NMR** (300 MHz, C_6D_6, 25 °C); 7.50-7.46 (^3J = 9.00, 1H, d, *Ar*C**H**,), 6.82-6.75 (^3J = 8.63, ^4J= 2.52 1H, td, *Ar*C**H**), 6.63-6.58 (^3J = 8.15, ^4J = 4.82, 1H, dd, *Ar*C**H**), 2.86 (^3J = 6.05, 2H, t, $C\mathbf{H_2}NH_2$), 2.18 (2H, b, $CH_2N\mathbf{H_2}$). ^{11}**B NMR** (96 MHz, C_6D_6, 25 °C); -6.92 (1B, t) ^{13}**C NMR** (125 MHz, C_6D_6, 25 °C); 134.6, 125.9, 122.2, 116.3, 116.0, 111.9, 111.6, 50.2. **HRMS** calcd. for C_7H_9BFN [M+H]+ (m/z): 136.0728, found: 136.0728. Melting Point 106-108°C

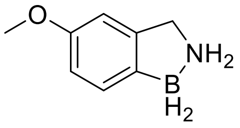

5-Methoxy-2,3-dihydrobenzo[1,2]azaborole, **13**

Compound **13** was synthesised according to general procedure **C** reacting amine-borane (500 mg, 2.39 mmol) and 3 equiv. $LiAlH_4$ (7.18 mL, 7.18 mmol) in THF (30 mL) at -78 °C. The reaction was warmed to room temperature and placed under reflux. The reaction mixture was quenched by adding water dropwise, very slowly. The precipitate was filtered and washed with THF and the organic fraction concentrated to yield a white solid (98% yield).

^{1}**H NMR** (300 MHz, DMSO, 25 °C); 7.14 – 7.11 (1H, d, *Ar*C**H**), 6.74 (1H, d, *Ar*C**H**), 6.69 – 6.66 (1H, dd, *Ar*C**H**), 6.00 (2H, b, $N\mathbf{H_2}$), 3.97 (^3J = 5.98, 2H, t, $C\mathbf{H_2}NH_2$) 3.73 (3H, s, $OC\mathbf{H_3}$). ^{11}**B NMR** (96 MHz, DMSO, 25 °C); -8.64. ^{13}C (125 MHz, DMSO. 25 °C); 157.2, 142.6, 129.2, 112.3, 106.9, 54.8, 49.5. ^{1}**H NMR** (300 MHz, C_6D_6, 25 °C); 7.68-7.65 (^3J =7.93, 1H, d, *Ar*C**H**,), 6.86-6.83 (^3J = 8.63, ^4J= 2.08 1H, dd, *Ar*C**H**), 6.60-6.59 (^3J = 1.25, 1H, d, *Ar*C**H**), 3.45 (3H, s, $OC\mathbf{H_3}$) 3.03 (^3J = 5.97, 2H, t, $C\mathbf{H_2}NH_2$), 2.57 (2H, b, $CH_2N\mathbf{H_2}$). ^{11}**B NMR** (96 MHz, C_6D_6, 25 °C); -6.67 (1B, t). ^{13}**C NMR** (125 MHz, C_6D_6, 25 °C); 158.63, 141.0, 130.7, 113.1, 107.2, 55.0, 50.8. **HRMS** calcd. for $C_8H_{12}BNO$ [M+H]+ (m/z): 148.0928, found: 148.0925. Melting Point 108-110 °C

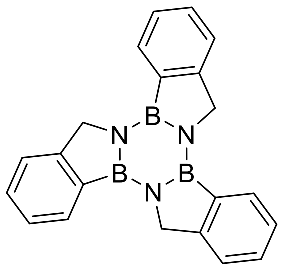

Borazatruxene, **1**

Compound **1** was synthesised according to general procedure **E**. Amine borane **13** (100 mg, 0.84 mmol) was charged into a microwave tube alongside a stir bar. Toluene was syringed into the tube and sonicated for 10 minutes. The tube was placed in a microwave and irradiated for 2 hours at 180 °C. The resulting precipitate was filtered and washed with toluene, THF and CH_2Cl_2. The product was isolated as a white precipitate (62%).

^{1}H (300 MHz, $CDCl_3$, 25 °C); 8.05 – 8.03 (^{3}J = 6.97, d, 1H *Ar*C**H**), 7.59 – 7.42 (m, 3H, *Ar*C**H**), 5.00 (s, 2H, C**H$_2$**). ^{11}B (96 MHz, $CDCl_3$, 25 °C); 34.5. ^{13}C (125 MHz, $CDCl_3$. 25 °C); 154.2, 131.0, 129.8, 126.5, 123.2, 52.4. ^{13}C (SSNMR); 154.4, 137.1, 129.9, 126.3, 123.1, 51.7. **HRMS** calcd. for $C_{21}H_{18}B_3N_3$ [M+H]+ (m/z): 346.1853, found: 346.1843.

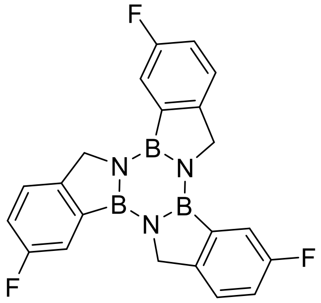

3,8,13-trifluoroborazatruxene, **2**

Compound **2** was synthesised according to general procedure **E**. Amine borane **14** (100 mg, 0.73 mmol) was charged into a microwave tube alongside a stir bar. Toluene was syringed into the tube and sonicated for 10 minutes. The tube was placed in a microwave and irradiated for 2 hours at 180 °C. The resulting precipitate was filtered and washed with toluene, THF and CH_2Cl_2. The product was isolated as a white precipitate (72%).

^{1}H (300 MHz, CDCl3, 25 °C); 7.65 - 7.62 ($^{3}J_{FH}$ =8.20, $^{4}J_{HH}$ = 2.55, dd, 1H *Ar*C**H**), 7.54 - 7.50 ($^{3}J_{FH}$ =8.34, $^{4}J_{HH}$ = 4.65, dd, 1H, *Ar*C**H**), 7.25 - 7.18 ($^{3}J_{HH,FH}$ = 17.62, ^{4}J = 2.43, td, 1H, *Ar*C**H**), 4.94 (s, 2H, C**H2**). ^{11}B (SSNMR); 25.8, 14.2, 1.4. ^{13}C (SSNMR); 160.8, 149.2, 138.0, 123.8, 116.0, 50.7. **HRMS** calcd. for $C_{21}H_{15}B_3F_3N_3$ [M+H]+ (m/z): 400.1571, found: 400.1571.

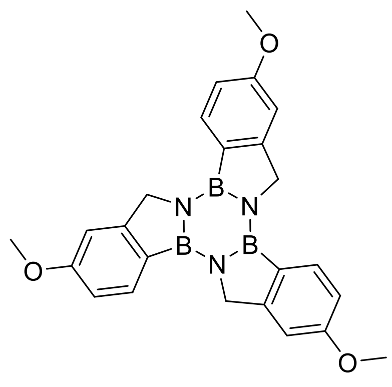

2,7,12-trimethoxyborazatruxene, **3**

Compound **3** was synthesised according to general procedure **E**. Amine borane **15** (100 mg, 0.67 mmol) was charged into a microwave tube alongside a stir bar. Toluene was syringed into the tube and sonicated for 10 minutes. The tube was placed in a microwave and irradiated for 2 hours at 180 °C. The resulting precipitate was filtered and washed with toluene, THF and CH_2Cl_2. The product was isolated as a white precipitate (65%).

^{1}H (300 MHz, CDCl3, 25 °C); 7.91 - 7.88 (3*J* = 8.07, d, 1H *Ar*C**H**), 7.07 - 7.06 (4*J* = 2.12, d, 1H, *Ar*CH), 7.00 - 6.97 (3*J* = 8.09, 4*J* = 2.24, dd, 1H, *Ar*C**H**), 4.91 (s, 2H, C**H2**) 3.90 (s, 3H, C**H3**). ^{11}B (SSNMR); 26.5, 14.7, 0.7. ^{13}C (SSNMR); 161.3, 158.2, 132.8, 129.8, 116.8, 107.6, 52.9. **HRMS** calcd. for $C_{24}H_{24}B_3N_3O_3$ [M+H]+ (m/z): 436.2170, found: 436.2168.

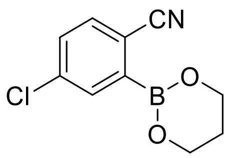

4-Chloro-2-(1,3,2-dioxaborinan-2-yl)benzonitrile **14**

Compound **14** was synthesised according to General Procedure **B**. 1,1,2,2-tetramethylpiperidine (0.29 mL, 1.74 mmol) was charged into a 2-neck RBF followed by THF (5 mL). The flask was cooled to 0 °C and *n*-BuLi (0.70 mL, 1.74 mmol) was added dropwise. The flask was further cooled to -78 °C where triisopropyl borate (0.47 mL, 2.04 mmol) was syringed into the reaction mixture. A solution of 4-chlorobenzonitrile (200 mg, 1.45 mmol) in THF (3 mL) was charged into the flask dropwise, noting a distinct colour change of yellow to red. Leaving the flask submerged in the dry ice/acetone bath, the reaction was allowed to mix overnight, slowly warming to room temperature. The reaction was quenched with AcOH (0.17 mL, 2.91 mmol) and stirred for 30 minutes. 1,3 propanediol (0.21 mL, 2.91 mmol) was added into the quenched reaction mixture and stirred for a further 30 minutes. The reaction was diluted with CH_2Cl_2 (20 mL) and washed with $KHPO_4$ (10% v/v, 3 x 10 mL), brine (1 x 10 mL) and then dried over $MgSO_4$. The reactants were filtered and concentrated. The crude reaction mixture was re-dissolved in CH_2Cl_2 (20 mL) and 1,3-propanediol (0.42 mL, 5.82 mmol) and

mixed for 1 hours. This was washed with water (3 x 10 mL) and dried over $MgSO_4$. The solution was filtered and concentrated to give purified desired product as a yellow solid (85%).

^{1}H (300 MHz, $CDCl_3$, 25 °C); 7.84-7.83 (^4J=2.16, 1H, d, *Ar*C**H**), 7.61-7.58 (^3J=8.26, 1H, d, *Ar*C**H**), 7.45-7.42 (^3J=8.25, ^4J=2.25, 1H, dd, *Ar*C**H**), 4.21 (^3J=5.46, 4H, t, -OC**H₂**CH₂C**H₂**O-) 2.13-2.06 (2H, m, -OCH₂C**H₂**CH₂O-). ^{11}B (96 MHz, $CDCl_3$, 25 °C); 29.1. ^{13}C (125 MHz, $CDCl_3$. 25 °C); 138.6, 135.4, 135.0, 130.8, 119.0, 114.7, 62.6, 27.3. **HRMS** calcd. for $C_{10}H_9BClNO_2$ [M+H]+ (m/z): 222.0488, found: 222.0488. Melting Point 141-143 °C.

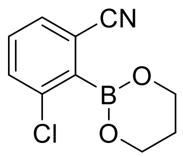

3-Chloro-2-(1,3,2-dioxaborinan-2-yl)benzonitrile **16**

Compound **16** was synthesised according to General Procedure **B.** 1,1,2,2-tetramethylpiperidine (0.29 mL, 1.74 mmol) was charged into a 2-neck RBF followed by THF (5 mL). The flask was cooled to 0 °C and *n*-BuLi (0.70 mL, 1.74 mmol) was added dropwise. The flask was further cooled to -78 °C where triisopropyl borate (0.47 mL, 2.04 mmol) was syringed into the reaction mixture. A solution of 3-chlorobenzonitrile (200 mg, 1.45 mmol) in THF (3 mL) was charged into the flask dropwise, noting a distinct colour change of yellow to red. Leaving the flask submerged in the dry ice/acetone bath, the reaction was allowed to mix overnight, slowly warming to room temperature. The reaction was quenched with AcOH (0.17 mL, 2.91 mmol) and stirred for 30 minutes. 1,3 propanediol (0.21 mL, 2.91 mmol) was added into the quenched reaction mixture and stirred for a further 30 minutes. The reaction was diluted with CH_2Cl_2 (20 mL) and washed with $KHPO_4$ (10% v/v, 3 x 10 mL), brine (1 x 10 mL) and then dried over $MgSO_4$. The reactants were filtered and concentrated. The crude reaction mixture was re-dissolved in CH_2Cl_2 (20 mL) and 1,3-propanediol (0.42 mL, 5.82 mmol) and mixed for 1 hours. This was washed with water (3 x 10 mL) and dried over $MgSO_4$. The solution was filtered and concentrated to give purified desired product as a yellow solid (87%).

^{1}H (300 MHz, $CDCl_3$, 25 °C); 7.54-7.51 (2H, m, *Ar*C**H**), 7.37-7.32 (^3J = 8.40, ^4J = 7.40, 1H, dd, *Ar*C**H**), 4.24 (4H, t, -OC**H₂**CH₂C**H₂**O-) 2.21-2.14 (2H, m, -OCH₂C**H₂**CH₂O-). ^{11}B (96 MHz, $CDCl_3$, 25 °C); 30.0. ^{13}C (125 MHz, $CDCl_3$. 25 °C); 133.0, 130.6, 130.4, 62.8, 27.3. **HRMS** calcd. for $C_{10}H_9BClNO_2$ [M+H]+ (m/z): 222.0488, found: 222.0486. Melting Point 112-114 °C.

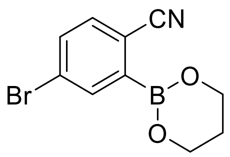

4-Bromo-2-(1,3,2-dioxaborinan-2-yl)benzonitrile **15**

Compound **15** was synthesised according to General Procedure **B**. 1,1,2,2-tetramethylpiperidine (0.22 mL, 1.31 mmol) was charged into a 2-neck RBF followed by THF (5 mL). The flask was cooled to 0 °C and *n*-BuLi (0.53 mL, 1.31 mmol) was added dropwise. The flask was further cooled to -78 °C where triisopropyl borate (0.36 mL, 1.54 mmol) was syringed into the reaction mixture. A solution of 4-bromobenzonitrile (200 mg, 1.10 mmol) in THF (3 mL) was charged into the flask dropwise, noting a distinct colour change of yellow to red. Leaving the flask submerged in the dry ice/acetone bath, the reaction was allowed to mix overnight, slowly warming to room temperature. The reaction was quenched with AcOH (0.13 mL, 2.20 mmol) and stirred for 30 minutes. 1,3 propanediol (0.16 mL, 2.20 mmol) was added into the quenched reaction mixture and stirred for a further 30 minutes. The reaction was diluted with CH_2Cl_2 (20 mL) and washed with $KHPO_4$ (10% v/v, 3 x 10 mL), brine (1 x 10 mL) and then dried over $MgSO_4$. The reactants were filtered and concentrated. The crude reaction mixture was re-dissolved in CH_2Cl_2 (20 mL) and 1,3-propanediol (0.32 mL, 4.40 mmol) and mixed for 1 hours. This was washed with water (3 x 10 mL) and dried over $MgSO_4$. The solution was filtered and concentrated to give purified desired product as a yellow solid (83%).

^{1}H (300 MHz, $CDCl_3$, 25 °C); 8.00 - 7.99 (^{4}J=1.86, 1H, d, *Ar*C**H**), 7.63 - 7.59 (^{3}J=7.44, ^{4}J=2.12, 1H, dd, *Ar*C**H**), 7.53 - 7.5 (^{3}J=8.23, 1H, d, *Ar*C**H**), 4.22 (^{3}J=5.42, 4H, t, -OC**H$_2$**CH$_2$C**H$_2$**O-) 2.13 - 2.06 (2H, m, -OCH$_2$C**H$_2$**CH$_2$O-). ^{11}B (96 MHz, $CDCl_3$, 25 °C); 28.9. ^{13}C (125 MHz, $CDCl_3$. 25 °C); 138.3, 135.0, 133.8, 127.3, 119.1, 115.2, 62.5, 27.2. **HRMS** calcd. for $C_{10}H_9BBrNO_2$ [M+H]+ (m/z): 265.9985, found: 265.9985. Melting Point 69 – 71 °C

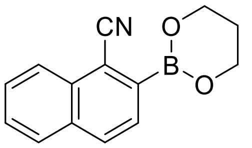

2-(1,3,2-dioxaborinan-2-yl)-1-naphthonitrile **24**

Compound **24** was synthesised according to General Procedure **B**. 1,1,2,2-tetramethylpiperidine (0.26 mL, 1.57 mmol) was charged into a 2-neck RBF followed by THF (5 mL). The flask was cooled to 0 °C and *n*-BuLi (0.63 mL, 1.57 mmol) was added dropwise. The flask was further cooled to -78 °C where triisopropyl borate (0.42 mL, 1.83 mmol) was syringed into the reaction mixture. A solution of 1-cyanonaphthalene (200 mg, 1.31 mmol) in THF (3 mL) was charged into the flask dropwise, noting a distinct colour change of yellow to red. Leaving the flask submerged in the dry ice/acetone bath, the reaction was allowed to mix overnight,

slowly warming to room temperature. The reaction was quenched with AcOH (0.15 mL, 2.61 mmol) and stirred for 30 minutes. 1,3 propanediol (0.37 mL, 5.22 mmol) was added into the quenched reaction mixture and stirred for a further 30 minutes. The reaction was diluted with CH_2Cl_2 (20 mL) and washed with $KHPO_4$ (10% v/v, 3 x 10 mL), brine (1 x 10 mL) and then dried over $MgSO_4$. The reactants were filtered and concentrated to give desired product as a white solid (81%).

^{1}H (300 MHz, $CDCl_3$, 25 °C); 8.37 – 8.34 (^3J=8.29, 1H, d, *Ar*C**H**), 8.02 – 7.99 (^3J=8.38, 1H, d, *Ar*C**H**), 7.90 - 7.88 (^3J=8.35, 2H, d, *Ar*C**H**), 7.70 - 7.58 (2H, m, *Ar*C**H**), 4.31 - 4.27 (^3J=5.51, 4H, t, -OC**H₂**CH₂C**H₂**O-) 2.19 - 2.12 (2H, m, -OCH₂C**H₂**CH₂O-). ^{11}B (96 MHz, $CDCl_3$, 25 °C); 29.7. ^{13}C (125 MHz, $CDCl_3$, 25 °C); 133.9, 133.2, 131.5, 130.0, 128.5, 128.3, 127.9, 126.0, 118.1, 115.0, 62.5, 27.4. **HRMS** calcd. for $C_{14}H_{12}BNO_2$ [M+H]+ (m/z): 238.1035, found: 238.1034. Melting Point 106 – 108 °C

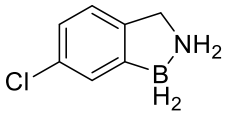

6-Chloro-2,3-dihydrobenzo[1,2]azaborole, **18**

Compound **18** was synthesised according to general procedure **C** reacting boronate ester **14** (200 mg, 0.90 mmol) and $LiAlH_4$ (2.71 mL, 2.71 mmol) in THF (10 mL) at -78 °C. The reaction was warmed to room temperature and placed under reflux for 3 hours. The reaction mixture was quenched by adding water dropwise, very slowly. The precipitate was filtered and washed with THF and the organic fraction concentrated to yield a white solid. Purification of the product was achieved following suspension in CH_2Cl_2 (20 mL), washing with 1 M HCl (3 x 10 mL) and drying over $MgSO_4$. The resultant organic layer was concentrated to isolate the white solid (61% yield).

^{1}H (300 MHz, DMSO, 25 °C); 7.13 - 7.11 (^4J=1.68, 1H, d, *Ar*C**H**), 7.09 – 7.07 (^3J=7.99, 1H, d, *Ar*C**H**), 7.00 – 6.97 (^3J=7.96, ^4J=2.06, 1H, dd, *Ar*C**H**), 6.06 (2H, br, N**H₂**), 3.94 (^3J=5.84, 2H, t, C**H₂**). ^{11}B (96 MHz, DMSO, 25 °C); -8.7. ^{13}C (125 MHz, DMSO, 25 °C); 140.1, 131.1, 128.0, 123.8, 122.9, 48.9. ^{1}**H NMR** (300 MHz, C_6D_6, 25 °C); 7.76 - 7.75 (^4J=1.50, 1H, d, *Ar*C**H**), 7.17 – 7.15 (^3J=7.96, ^4J=1.95, 1H, dd, *Ar*C**H**), 6.56 – 6.53 (^3J=8.11, 1H, d, *Ar*C**H**), 2.84 (^3J=6.08, 2H, t, C**H₂**), 2.24 (2H, br, N**H₂**). ^{11}**B NMR** (96 MHz, C_6D_6, 25 °C); -6.9 (1B, t). ^{13}**C NMR** (125 MHz, C_6D_6, 25 °C); 137.6, 133.8, 129.9, 125.9, 125.0, 122.4, 50.2. **HRMS** calcd. for C_7H_9BClN [M+H]+ (m/z): 152.0433, found: 152.0432. Melting Point 141 – 143 °C.

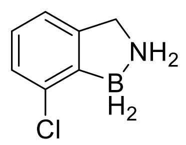

7-Chloro-2,3-dihydrobenzo[1,2]azaborole, **20**

Compound **20** was synthesised according to general procedure C reacting boronate ester **16** (200 mg, 0.90 mmol) and $LiAlH_4$ (1.0 mol in THF, 2.71 mL, 2.71 mmol) in THF (10 mL) at -78 °C. The reaction was warmed to room temperature and placed under reflux for 3 hours. The reaction mixture was quenched by adding water dropwise, very slowly. The precipitate was filtered and washed with THF and the organic fraction concentrated to yield a white solid. Purification of the product was achieved following suspension in CH_2Cl_2 (20 mL), washing with 1 M HCl (3 x 10 mL) and drying over $MgSO_4$. The resultant organic layer was concentrated to isolate the white solid (66% yield).

^{1}H (300 MHz, DMSO, 25 °C); 7.01 (3H, m, *Ar*C**H**), 6.07 (2H, br, N**H₂**), 4.02 (2H, t, C**H₂**). ^{11}B (96 MHz, DMSO, 25 °C); -8.8. ^{13}C (125 MHz, DMSO, 25 °C); 143.5, 134.9, 126.4, 125.9, 119.7, 49.8. ^{1}**H NMR** (300 MHz, C_6D_6, 25 °C); 7.35 - 7.32 (^{3}J=7.84, 1H, d, *Ar*C**H**), 6.92 (^{3}J=8.11, 1H, t, *Ar*C**H**), 6.65 – 6.63 (^{3}J=7.39, 1H, d, *Ar*C**H**), 3.08 (^{3}J=5.97, 2H, t, C**H**$_2$), 2.54 (2H, br, N**H**$_2$). ^{11}**B NMR** (96 MHz, C_6D_6, 25 °C); -7.0 (1B, t). ^{13}**C NMR** (125 MHz, C_6D_6, 25 °C); 141.7, 137.2, 127.3, 119.8, 51.4. **HRMS** calcd. for C_7H_9BClN [M+H]+ (m/z): 152.0433, found: 152.0430. Melting Point 112-114 °C

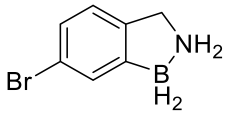

6-Bromo-2,3-dihydrobenzo[1,2]azaborole, **19**

Compound **19** was synthesised according to general procedure **C** reacting boronate ester **15** (200 mg, 0.75 mmol) and $LiAlH_4$ (1.0 mol in THF, 2.26 mL, 2.26 mmol) in THF (10 mL) at -78 °C. The reaction was warmed to room temperature and placed under reflux for 3 hours. The reaction mixture was quenched by adding water dropwise, very slowly. The precipitate was filtered and washed with THF and the organic fraction concentrated to yield a white solid. Purification of the product was achieved following suspension in CH_2Cl_2 (20 mL), washing with 1M HCl (3 x 10 mL) and drying over $MgSO_4$. The resultant organic layer was concentrated to isolate the white solid (64% yield).

^{1}H (300 MHz, DMSO, 25 °C); 7.28-7.27 (^{4}J=1.50, 1H, d, *Ar*C**H**), 7.14-7.11 (^{3}J=7.94, ^{4}J=1.93, 1H, dd, *Ar*C**H**), 7.04-7.01 (^{3}J=7.98, 1H, d, *Ar*C**H**), 6.04 (2H, br, N**H**$_2$), 3.90 (^{3}J=5.96, 2H, t, C**H**$_2$). ^{11}B (96 MHz, DMSO, 25 °C); -8.7. ^{13}C (125 MHz, DMSO, 25 °C); 140.5, 130.9, 126.6, 123.4, 120.2, 48.9. ^{1}**H NMR** (300 MHz, C_6D_6, 25 °C); 7.95 - 7.94 (^{4}J=1.32, 1H, d, *Ar*C**H**), 7.28 – 7.24 (^{3}J=8.03, ^{4}J=1.85, 1H, dd, *Ar*C**H**), 6.50 – 6.47 (^{3}J=7.90, 1H, d, *Ar*C**H**), 2.76 (^{3}J=6.11, 2H, t, C**H**$_2$), 2.01 (2H, br, N**H**$_2$). ^{11}**B NMR** (96 MHz, C_6D_6, 25 °C); -7.1 (1B, t). ^{13}**C NMR** (125 MHz, C_6D_6,

25 °C); 138.2, 133.3, 127.2, 126.0, 123.1, 50.7. **HRMS** calcd. for C_7H_9BBrN [M+H]+ (m/z): 197.9908, found: 199.9907. Melting Point 147-149 °C

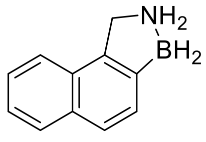

2,3-dihydronaphtho[1,2]azaborole, **25**

Compound **25** was synthesised according to general procedure **C** reacting boronate ester **24** (200 mg, 0.84 mmol) and $LiAlH_4$ (2.53 mL, 2.53 mmol) in THF (10 mL) at -78 °C. The reaction was warmed to room temperature and placed under reflux for 2 days. The reaction mixture was quenched by adding water dropwise, very slowly. The precipitate was filtered and washed with THF and the organic fraction concentrated to yield a white solid. Purification of the product was achieved following suspension in CH_2Cl_2 (20 mL), washing with 1M HCl (3 x 10 mL) and drying over $MgSO_4$. The resultant organic layer was concentrated to isolate the white solid (85% yield).

^{1}H (300 MHz, DMSO, 25 °C); 7.94 – 7.32 (6H, m, *Ar*C**H**), 6.18 (2H, br, N**H₂**), 4.38 (^{3}J=6.07, 2H, t, C**H₂**). ^{11}B (96 MHz, DMSO, 25 °C); -9.4. ^{13}C (125 MHz, DMSO, 25 °C); ^{1}H (300 MHz, C_6D_6, 25 °C); 8.01 - 7.98 (^{3}J=4.48, 1H, d, *Ar*C**H**), 7.79 - 7.76 (^{3}J=8.05, 1H, d, *Ar*C**H**), 7.72 - 7.70 (^{3}J=7.57, 1H, d, *Ar*C**H**), 7.36 - 7.22 (4H, m, *Ar*C**H**), 3.35 (^{3}J=7.99, 2H, t, C**H₂**) 2.44 (2H, b, N**H₂**). ^{11}B (96 MHz, C_6D_6, 25 °C); -7.1 (1B, t). ^{13}C (125 MHz, C_6D_6, 25 °C); 133.3, 132.9, 129.2, 129.2, 129.1, 128.9, 127.5, 125.9, 125.6, 124.0, 122.6, 49.8. **HRMS** calcd. for $C_{11}H_{12}BN$ [M-H]+ (m/z): 168.0979, found: 168.0975. Melting Point 140 – 142 °C.

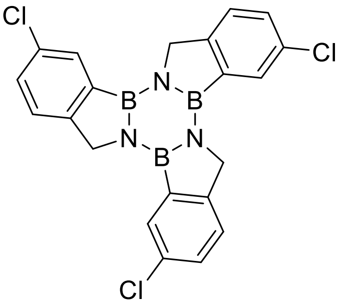

3,8,13-trichloroborazatruxene, **4**

Compound **4** was synthesised according to general procedure **E**. Amine borane **18** (100 mg, 0.65 mmol) was charged into a microwave tube alongside a stir bar. Toluene was syringed into the tube and sonicated for 10 minutes. The tube was placed in a microwave and irradiated for 2 hours at 180 °C. The resulting precipitate was filtered and washed with toluene, THF and CH_2Cl_2. The product was isolated as a white precipitate (63%).

^{1}H (300 MHz, DMSO-d_6, 85 °C); 8.05 (s, 1H *Ar*C**H**), 7.68 - 7.67 (d, 1H, *Ar*C**H**), 7.59 - 7.58 (d, 1H, *Ar*C**H**), 5.06 (s, 2H, C**H2**). ^{11}B (SSNMR); 25.6, 14.3. ^{13}C (SSNMR); 152.0, 137.4, 135.8, 130.2, 124.4, 51.0. **HRMS** calcd. for $C_{21}H_{15}B_3Cl_3N_3$ [M+H]+ (m/z): 448.0699, found: 448.071.

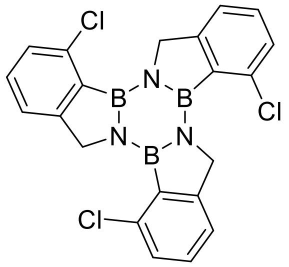

4,9,14-trichloroborazatruxene, **6**

Compound **6** was synthesised according to general procedure **E**. Amine borane **20** (100 mg, 0.65 mmol) was charged into a microwave tube alongside a stir bar. Toluene was syringed into the tube and sonicated for 10 minutes. The tube was placed in a microwave and irradiated for 2 hours at 180 °C. The resulting precipitate was filtered and washed with toluene, THF and CH_2Cl_2. The product was isolated as a white precipitate (65%).

^{11}B (SSNMR); 26.6, 15.5. ^{13}C (SSNMR); 155.7, 138.5, 133.5, 131.2, 127.7, 119.9, 56.4. **HRMS** calcd. for $C_{21}H_{15}B_3Cl_3N_3$ [M+H]+ (m/z): 448.0699, found: 448.0702.

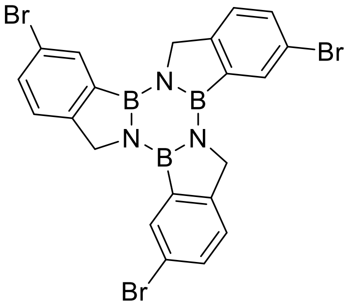

3,8,13-tribromoborazatruxene, **5**

Compound **5** was synthesised according to general procedure **E**. Amine borane **19** (100 mg, 0.51 mmol) was charged into a microwave tube alongside a stir bar. Toluene was syringed into the tube and sonicated for 10 minutes. The tube was placed in a microwave and irradiated for 2 hours at 180 °C. The resulting precipitate was filtered and washed with toluene, THF and CH_2Cl_2. The product was isolated as a white precipitate (73%).

^{11}B (SSNMR); 25.3, 14.1, 3.6. ^{13}C (SSNMR); 150.8, 137.3, 130.7, 124.9, 122.2, 51.8. **HRMS** calcd. for $C_{21}H_{15}B_3Br_3N_3$ [M+H]+ (m/z): 581.9166, found: 581.9179.

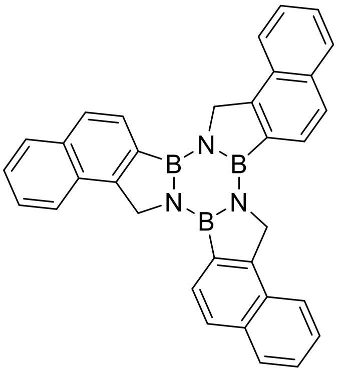

Tribenzo[*c,c′,c″*]borazatruxene, **8**

Compound **8** was synthesised according to general procedure **E**. Amine borane **25** (100 mg, 0.59 mmol) was charged into a microwave tube alongside a stir bar. Toluene was syringed into the tube and sonicated for 10 minutes. The tube was placed in a microwave and irradiated for 2 hours at 180 °C. The resulting precipitate was filtered and washed with toluene, THF and CH_2Cl_2. The product was isolated as a white precipitate (55%).

^{11}B (SSNMR); 25.9, 14.8, 5.6. ^{13}C (SSNMR); 152.2, 140.4, 133.3, 128.0, 125.42, 50.4. **HRMS** calcd. for $C_{33}H_{24}B_3N_3$ [M+H]+ (m/z): 496.2323, found: 496.2330.

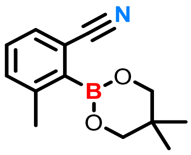

2-(5,5-dimethyl-1,3,2-dioxaborinan-2-yl)-3-methylbenzonitrile (103 mg, 0.2 mmol), **17**

Procedure adapted from Rochais's original protocol.[4] In a flame-dried 50 mL two necked flask equipped with condenser, under argon atmosphere, 2-bromo-3-methylbenzonitrile (800 mg, 4.1 mmol), bis(neopentyl glycolato)diboron (1383 mg, 6.1 mmol, 1.5 equiv.), Pd(dppf)Cl_2 (233 mg, 0.3 mmol, 7 mmol%) and CH_3COOK (1602 mg, 16.3 mmol, 4.0 equiv.) were vacuumed for 1 h and back filled with argon, then dissolved in anhydrous 1,4-dioxane (20 mL) and heated up under reflux. After 1 h, the reaction mixture was cooled to room temperature and the organic solvent was removed under reduced pressure. The residue was re-dissolved in EtOAc (30 mL), transferred to a separating funnel and washed with distilled water (2 x 20 mL) and brine (1 x 20 mL). The combined water phase was back-extracted once with EtOAc (20 mL) while the combined organic phase was dried over $MgSO_4$, filtered and the solvent removed under reduced pressure. The crude product was purified by silica gel flash chromatography (n-hexane / CH_2Cl_2 70 : 30 to CH_2Cl_2 100) to afford the desired **17** as a white solid (830 mg, 89%). ***^{1}H NMR***: (500 MHz, $CDCl_3$) δ (ppm) 7.47 (d, *J*=7.5 Hz, 1H), 7.34-7.28 (m, 2H), 3.84 (s, 4H), 2.46 (s, 3H), 1.11 (s, 6H). ***^{13}C NMR***: (125 MHz, $CDCl_3$) δ (ppm) 142.8, 133.6, 130.2, 129.3, 120.1, 116.0, 72.7, 31.9, 22.2, 22.1. ***^{11}B NMR***: (160 MHz, $CDCl_3$) δ (ppm) 27.4. ***HRMS*** calc. for C13H16BNO2 $[M+H]^+$ ($^m/_z$): 230.1347, found: 230.1350.

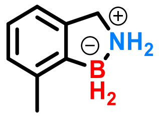

7-methyl-2,3-dihydro-1H-benzo[c][1,2]azaborole, **21**

Compound **21** was synthesized according to the general **PROCEDURE D** reacting 2-(5,5-dimethyl-1,3,2-dioxaborinan-2-yl)-3-methylbenzonitrile (103 mg, 0.2 mmol) and $LiAlH_4$ (≈1.0 M in THF) (2.2 mL, 2.2 mmol, 5.0 equiv.) in anhydrous THF (2.0 mL) under argon atmosphere. The desired product was obtained as a white powder (56 mg, 95%). ***^{1}H NMR***: (500 MHz, $CDCl_3$) δ (ppm) 7.06-7.01 (m, 2H), 6.94 (d, *J*=7.0 Hz, 1H), 4.15 (t, *J*=6.5 Hz, 2H), 3.96 (br, 2H), 2.32 (s, 3H). ***^{13}C NMR***: (125 MHz, $CDCl_3$) δ (ppm) 139.7, 138.4, 127.7, 125.7, 118.6, 51.5, 22.1. ***^{11}B NMR***: (160 MHz, $CDCl_3$) δ (ppm) -10.4 (t, *J*=85 Hz). ***HRMS*** calc. for C8H12BN $[M-H]^+$ ($^m/_z$): 132.0979, found: 132.0975.

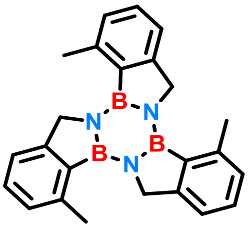

4,9,15-trimethylborazatruxene, **7**

Compound **7** was synthesized according to the general **PROCEDURE E** reacting 7-methyl-2,3-dihydro-1H-benzo[c][1,2]azaborole (**21**, 100 mg, 0.75 mmol) in anhydrous toluene (3 mL) under argon atmosphere. After 2 h, the reaction mixture was cooled to room temperature, diluted with EtOAc (20 mL) and transferred in a 100 mL flask. After the organic solvents were removed under reduced pressure, the crude product was purified by washing the solid with (n-hexane / CH_2Cl_2 80 : 20) (4 x 5 mL) to afford the desired **7** as a white solid (70 mg, 72%). ***^{1}H NMR***: (400 MHz, TCE-d_2, 75 °C) δ (ppm) 7.42 (d, *J*=5.0 Hz, 6H), 7.25 (t, *J*=5.0 Hz, 3H), 5.18 (s, 6H), 2.97 (s, 9H). ***^{13}C NMR***: (126 MHz, TCE-d_2, 80 °C) δ (ppm) 154.7, 140.7, 129.8, 128.8, 120.2, 56.2, 25.6. ***^{11}B NMR***: (128 MHz, TCE-d_2, 75 °C) δ (ppm) 36.1. ***HRMS*** calc. for C24H24B3N3 $[M+H]^+$ ($^m/_z$): 388.2322, found: 388.2326.

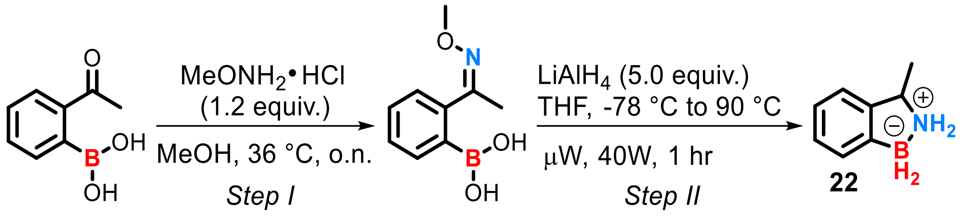

In the first step the procedure was adapted from Gillingham's original protocol.[5] In a flame-dried 10 mL two necked flask, under argon atmosphere, (2-acetylphenyl)boronic acid (100 mg, 0.6 mmol) and methoxyamine hydrochloride (60 mg, 0.7 mmol, 1.2 equiv.) were was dissolved in anhydrous methanol (4 mL) and the mixture was stirred at 36 °C overnight. After cooling to room temperature, the organic solvent was removed under reduced pressure, the residue was re-dissolved in anhydrous THF (3 mL) and transferred via cannula in a beforehand flame-dried 10 mL µW tube equipped with a stirring bar and sealed with a perforated plastic cap under argon atmosphere. The system was cooled to -78°C and after 15 min, freshly titrated $LiAlH_4$ (≈1.0 M in THF) (3.0 mL, 3.0 mmol, 5.0 equiv.) was added dropwise under vigorous stirring, and then the resulting mix was slowly allowed to warm to room temperature. The needle was removed to seal, and the µW tube was placed into the µW machine and the mixture was stirred and irradiated by microwave dielectric heating at 90 °C (power 40W). After 1 h, the reaction mixture was cooled to room temperature, diluted with anhydrous THF and transferred in a 100 mL conical flask. After cooling to -5 °C, the reaction was quenched dropwise with water under vigorous stirring yielding a white precipitate. The formed suspension was filtered on a plug of $MgSO_4$ and the solid was washed with THF (3 x 10 mL) and EtOAc (3 x 10 mL). After the organic solvents were removed under reduced pressure, the residue was re-dissolved in EtOAc (20 mL), transferred to a separating funnel and washed with distilled water (6 x 10 mL) and brine (1 x 10 mL). The combined water phase was back-extracted once with EtOAc (15 mL) while the combined organic phase was dried over $MgSO_4$, filtered and the solvent removed under reduced pressure to afford the desired **26** as a white solid (56 mg, 70% over 2 steps). ***^{1}H NMR***: (500 MHz, $CDCl_3$) δ (ppm) 7.46 (d, *J*=7.5 Hz, 1H), 7.22 (t, *J*=7.5 Hz, 1H), 7.14 (t, *J*=7.0 Hz, 1H), 7.07 (d, *J*=7.5 Hz, 1H), 4.57-4.50 (m, 1H), 4.39 (br, 2H), 1.61 (d, *J*=6.5 Hz, 3H). ***^{13}C NMR***: (125 MHz, $CDCl_3$) δ (ppm) 144.0, 129.6, 127.4, 125.4, 121.1, 59.0, 20.6. ***^{11}B NMR***: (160 MHz, $CDCl_3$) δ (ppm) -11.0 (t, *J*=96 Hz). ***HRMS*** calc. for C8H12BN $[M+H]^+$ ($^m/_z$): 132.0979, found: 132.0975.

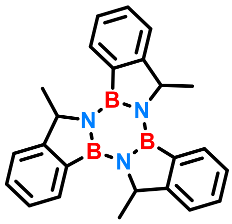

5,10,15-trimethylborazatruxene, **9**

Compound **9** was synthesized according to the general **PROCEDURE E** reacting 3-methyl-2,3-dihydro-1H-benzo[c][1,2]azaborole **22** (100 mg, 0.75 mmol) in anhydrous toluene (3 mL) under argon atmosphere. After 2 h, the reaction mixture was cooled to room temperature, diluted with EtOAc (20 mL) and transferred in a 100 mL flask. The organic solvents were removed under reduced pressure to afford the desired **9** as a white solid (90 mg, 93%). By washing the solid with a mix of n-hexane / CH_2Cl_2 (80 : 20) (3 x 2 mL) was possible to isolate the *syn* diastereoisomers from the *anti*, showing more affinity for the mix utilized.

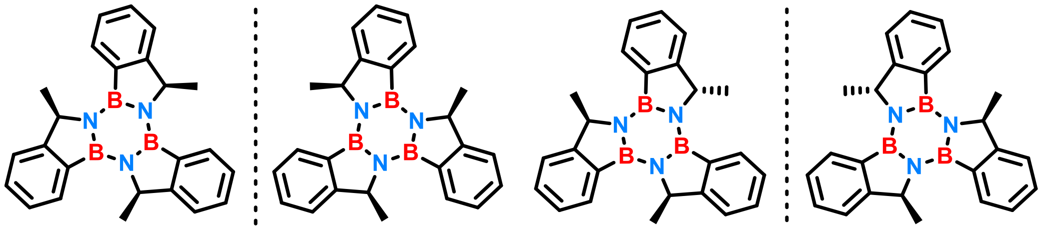

(5*R*,10*R*,15*R*)-9 **(5*S*,10*S*,15*S*)-9** **(5*R*,10*R*,15*S*)-9** **(5*S*,10*S*,15*R*)-9**

Syn: ***^{1}H NMR***: (500 MHz, $CDCl_3$) δ (ppm) 8.03 (d, *J*=7.5 Hz, 3H), 7.54-7.49 (m, 6H), 7.44 (t, *J*=7.0 Hz, 3H), 5.27-5.23 (q, *J*=7.0 Hz, 3H), 1.78 (d, *J*=7.0 Hz, 9H). ***Anti***: ***^{1}H NMR***: (500 MHz, $CDCl_3$) δ (ppm) 8.04 (d, *J*=7.5 Hz, 3H), 7.54-7.50 (m, 6H), 7.44 (t, *J*=7.0 Hz, 3H), 5.36-5.32 (q, *J*=7.0 Hz, 3H), 1.75 (d, *J*=6.5 Hz, 3H), 1.70 (d, *J*=6.5 Hz, 3H), 1.65 (d, *J*=6.5 Hz, 3H). ***^{13}C NMR***: (125 MHz, $CDCl_3$) δ (ppm) 160.0, 131.7, 129.9, 126.8, 122.9, 58.9, 24.7. ***^{11}B NMR***: (160 MHz, $CDCl_3$) δ (ppm) 34.4. ***HRMS*** calc. for C24H24B3N3 $[M+H]^+$ ($^{m}/_{z}$): 388.2322, found: 388.2322.

HPLC data for compound 9

Chiral HPLC separation of the of the *syn* enantiomers pair

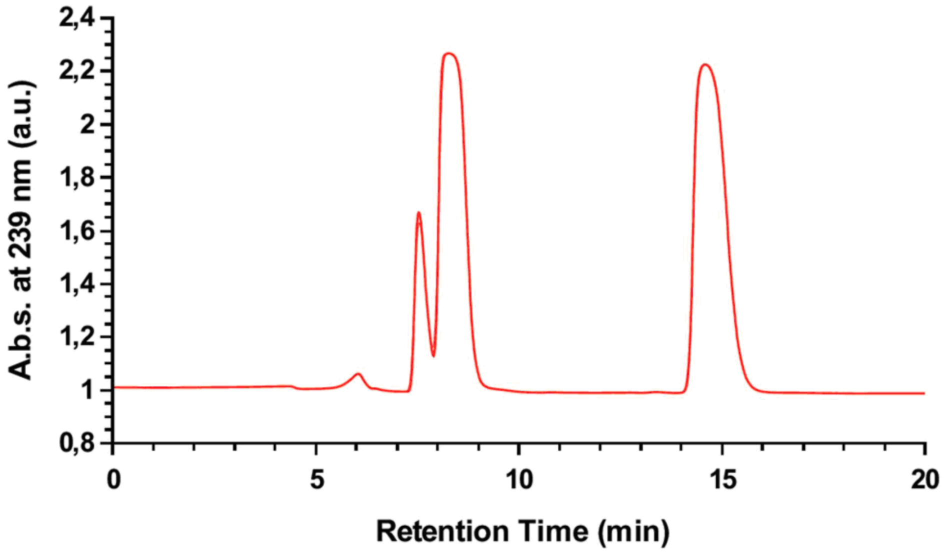

Figure S1 | HPLC chromatogram related to the *syn* enantiomers mixture obtained loading 30µL of the *syn* solution and eluting with n-hexane : isopropyl alcohol (98:2) at the flow rate of 0.5 mL/min. The UV detector was set up at 239 nm the optimum absorbance wavelength for the aromatic rings.

Autosampler Method
Injection Source : Autosampler
Injection volume : 30.0 µL
Loop size : 200 µL
Fixed mode : Off
Excess volume : 10 µL
Air cushion : 10 µL
Sample syringe size : 250 µL
Sample speed : Medium

Detector Parameters
A (nm) : 239 nm
BWA (nm) : 20 nm
RWA (nm) : 360 nm

B (nm) : 279 nm
BWB (nm) : 20 nm
RWB (nm) : 360 nm

Pump Parameters

Step	Time	Flow	Hexane	CH_3CN	MeOH	IPA	Curve
0	1.0	0.5	98.0	0.0	0.0	2.0	0.0
1	20.0	0.5	98.0	0.0	0.0	2.0	0.0

Table S1 | HPLC optimal conditions found for the method developed for the separation of the *syn* enantiomers

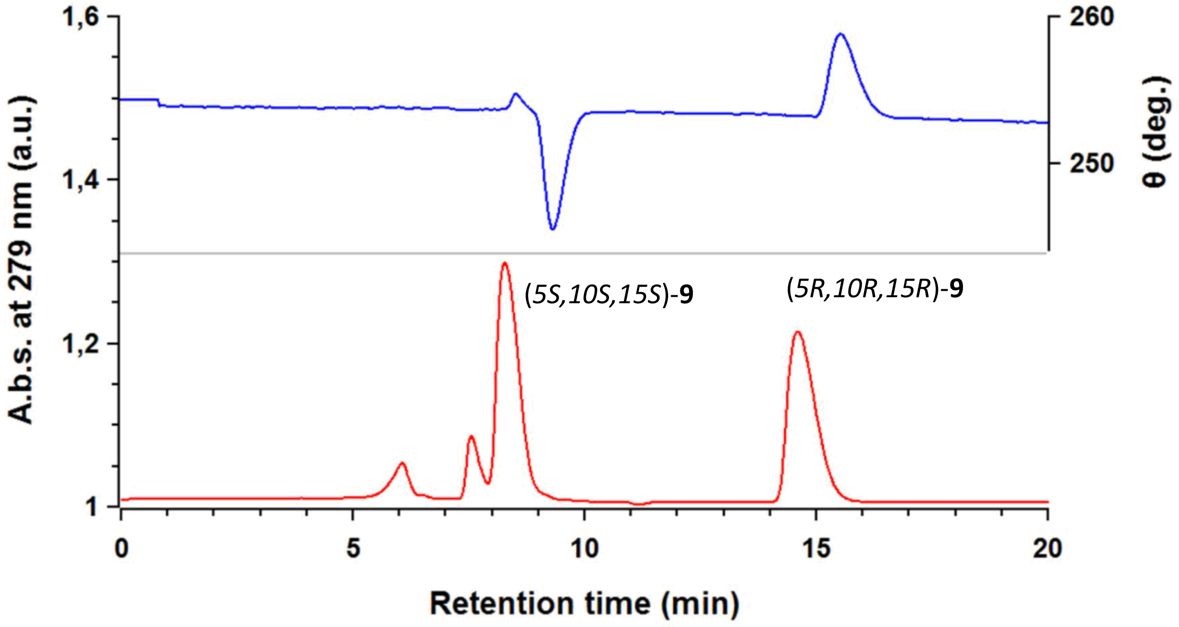

Figure S2 | HPLC chromatogram of the *syn* enantiomers mixture obtained in the same operative conditions of the previous example with the detector set up at 279 nm, the optimum absorbance wavelength for the borazine ring. A second optical rotation detector was utilized to detect the chirality of the two enantiomers. The polarogram overlaid on the chromatogram display the two main peaks that rotate symmetrically the polarized light in the opposite position confirming the opposite chirality.

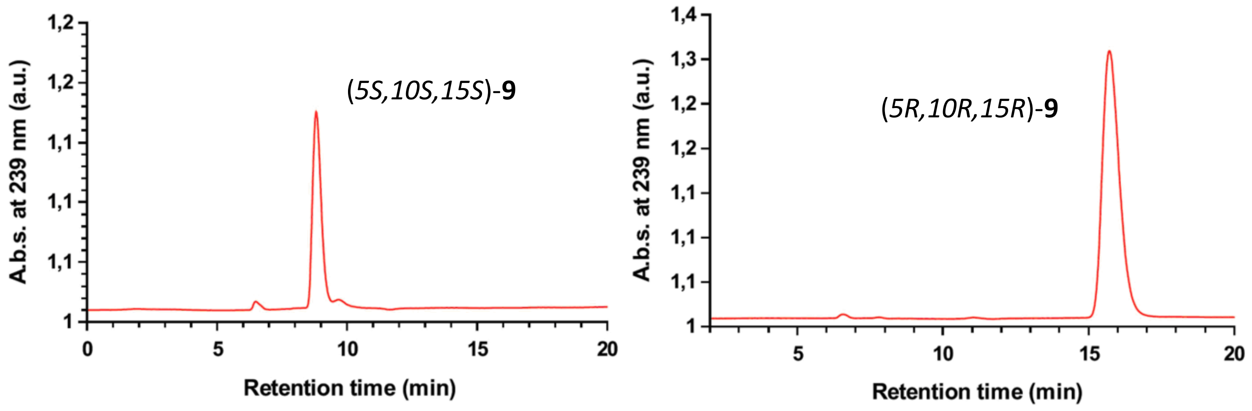

Figure S3 | HPLC chromatogram of the two *syn* enantiomers isolated by multiple injections of the mixture and collection of the two fractions separately.

Chiral HPLC separation of the of the *anti* enantiomers pair.

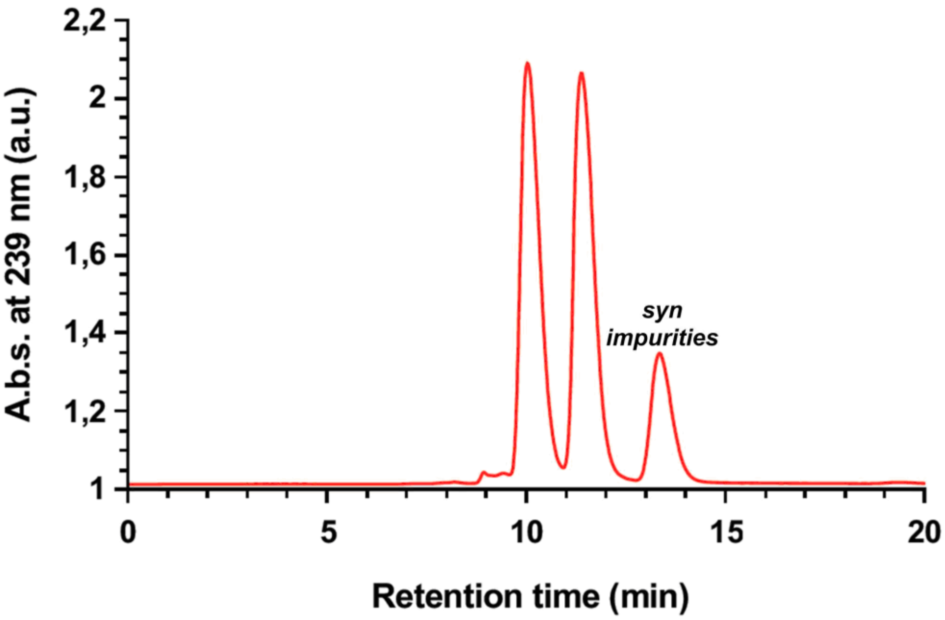

Figure S4 | HPLC chromatogram related to the *anti* enantiomers mixture obtained loading 10µL of the *anti* solution and eluting with n-hexane : isopropyl alcohol (99:1) at the flow rate of 0.5 mL/min. The UV detector was set up at 239 nm the optimum absorbance wavelength for the aromatic rings. According with the ^{1}H-NMR spectrum of the *anti* mixture isolated, *syn* enantiomers impurities are also detected in the HPLC chromatogram

Autosampler Method
Injection Source : Autosampler
Injection volume : 10.0 µL
Loop size : 200 µL
Fixed mode : Off
Excess volume : 10 µL
Air cushion : 10 µL
Sample syringe size : 250 µL

Detector Parameters
A (nm) : 239 nm
BWA (nm) : 20 nm
RWA (nm) : 360 nm

B (nm) : 279 nm
BWB (nm) : 20 nm
RWB (nm) : 360 nm

Pump Parameters

Step	Time	Flow	Hexane	CH_3CN	MeOH	IPA	Curve
0	15.0	0.5	99.0	0.0	0.0	1.0	0.0
1	20.0	0.5	99.0	0.0	0.0	1.0	0.0

Table S2 | HPLC optimal conditions found for the method developed for the separation of the *anti* enantiomers

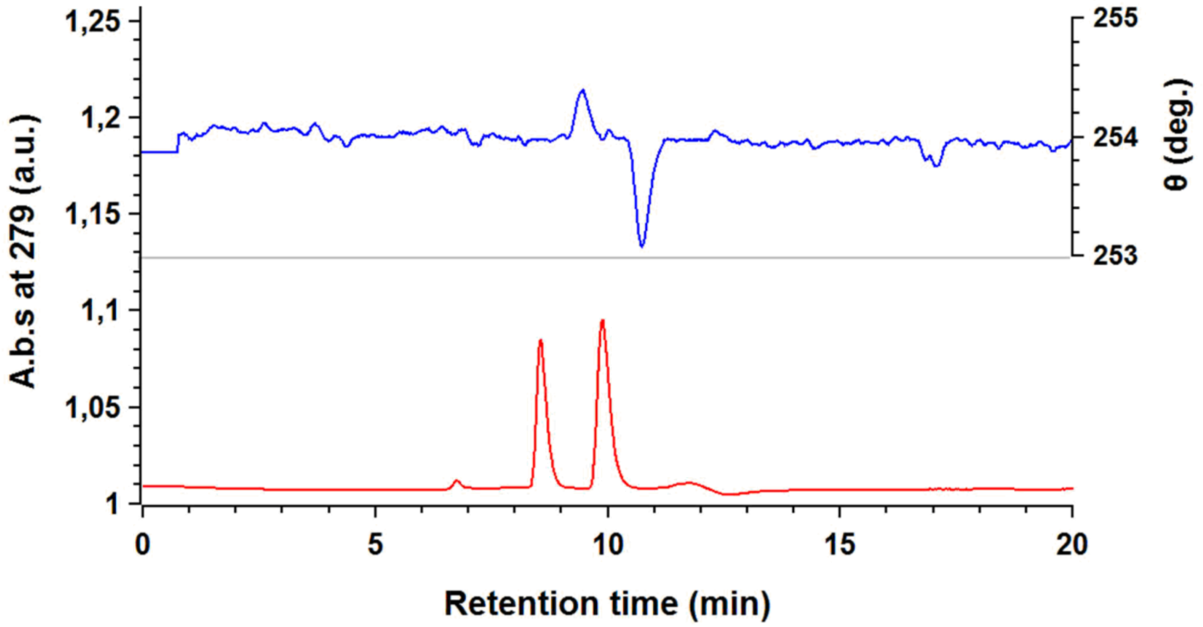

Figure S5 | HPLC chromatogram of the *anti* enantiomers mixture obtained in the same operative conditions of the previous example with the detector set up at 279 nm, the optimum absorbance wavelength for the borazine ring. A second optical rotation detector was utilized to detect the chirality of the two enantiomers. The polarogram overlaid on the chromatogram display the two main peaks that rotate symmetrically the polarized light in the opposite position confirming the opposite chirality.

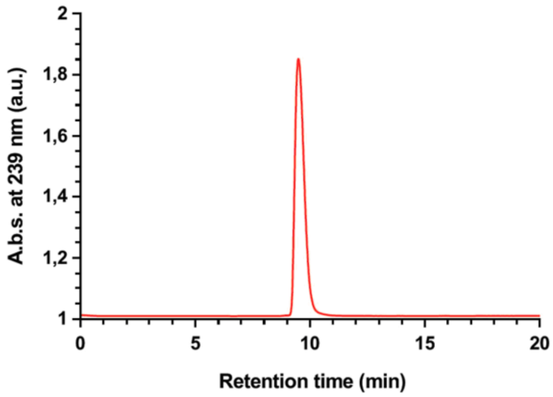

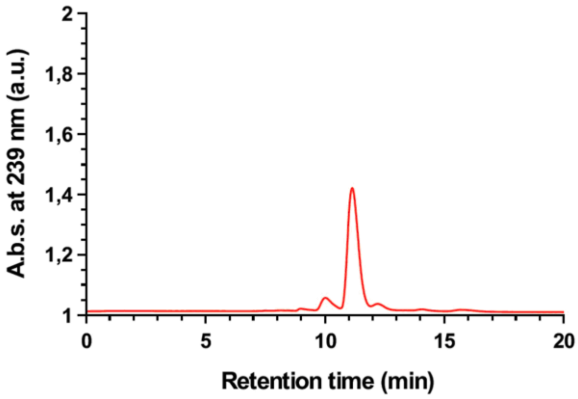

Figure S6 | HPLC chromatograms of the two *anti* enantiomers isolated by semi-prep HPLC.

Autosampler Method
Injection Source : Autosampler
Injection volume : 100.0 µL
Loop size : 200 µL
Fixed mode : Off
Excess volume : 10 µL
Air cushion : 10 µL
Sample syringe size : 250 µL

Detector Parameters
A (nm) : 239 nm
BWA (nm) : 20 nm
RWA (nm) : 360 nm

B (nm) : 279 nm
BWB (nm) : 20 nm
RWB (nm) : 360 nm

Pump Parameters

Step	Time	Flow	Hexane	CH_3CN	MeOH	IPA	Curve
0	20.0	1.20	99.0	0.0	0.0	1.0	0.0
1	20.0	0.40	99.0	0.0	0.0	1.0	0.0
2	3.0	0.80	80.0	0.0	0.0	20.0	0.0
3	15.0	1.20	80.0	0.0	0.0	20.0	0.0
4	3.0	1.20	99.0	0.0	0.0	1.0	0.0
5	5.0	1.20	99.0	0.0	0.0	1.0	0.0

Table S3 | Optimal semi-preparative HPLC conditions for the collection of the two *anti* enantiomers

CD data for compound 9

Circular Dichroism (CD) spectra of the *syn* enantiomeric species collected.

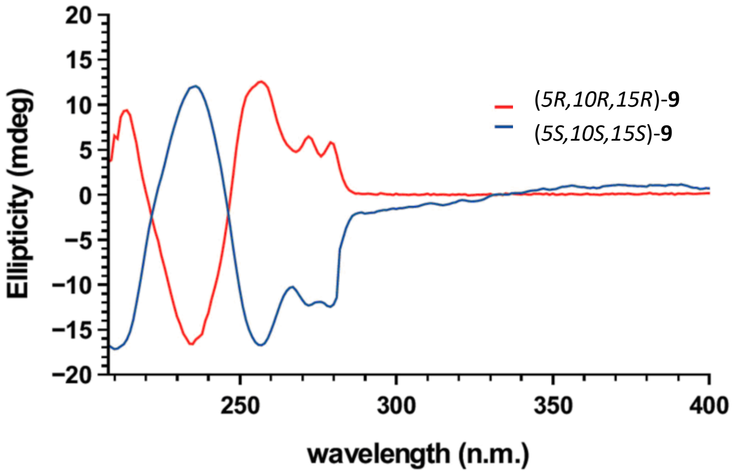

Figure S7 | The Circular Dichroism (CD) spectra collected for the *syn* enantiomeric pair display an intense induced CD signal. The first enantiomer isolated (blue) absorbs with a positive band in the aromatic absorption region (239 nm) and a negative band in the borazine region (272-279 nm) while the second enantiomer (red) displays opposite and symmetric CD maximum.

syn

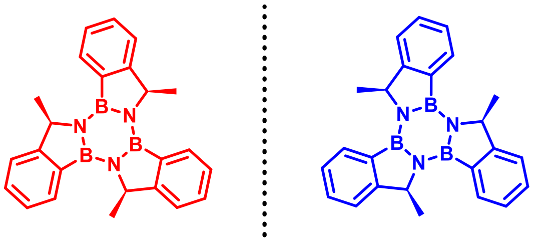

(5*R*,10*R*,15*R*)-9 **(5*S*,10*S*,15*S*)-9**

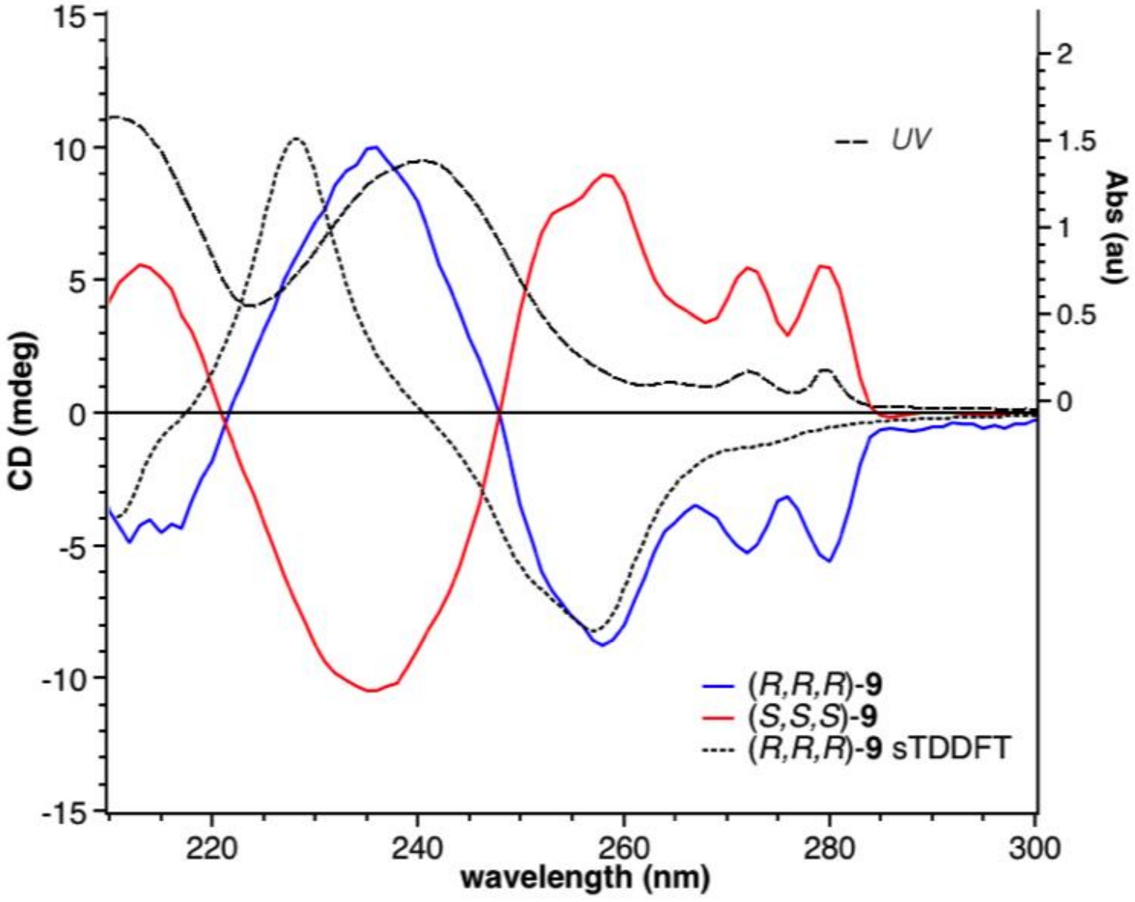

Figure S8 | The Circular Dichroism (CD) spectra (blue and red) displays a good match with the computed CD data (dotted) calculated for (*R,R,R*)-**9** using the sTDDFT method with M06-2X exchange correlational with 6311G(dp) basis sets. The UV-Vis absorption spectrum (dashed black) is also displayed in the graph. Comparing the experimental with the computed data was then possible to assign the absolute configuration to the two *syn* enantiomers (*SSS*-**9** and *RRR*-**9**) collected in the first and the second fractions.

Circular Dichroism (CD) spectra of the *anti* enantiomeric species collected.

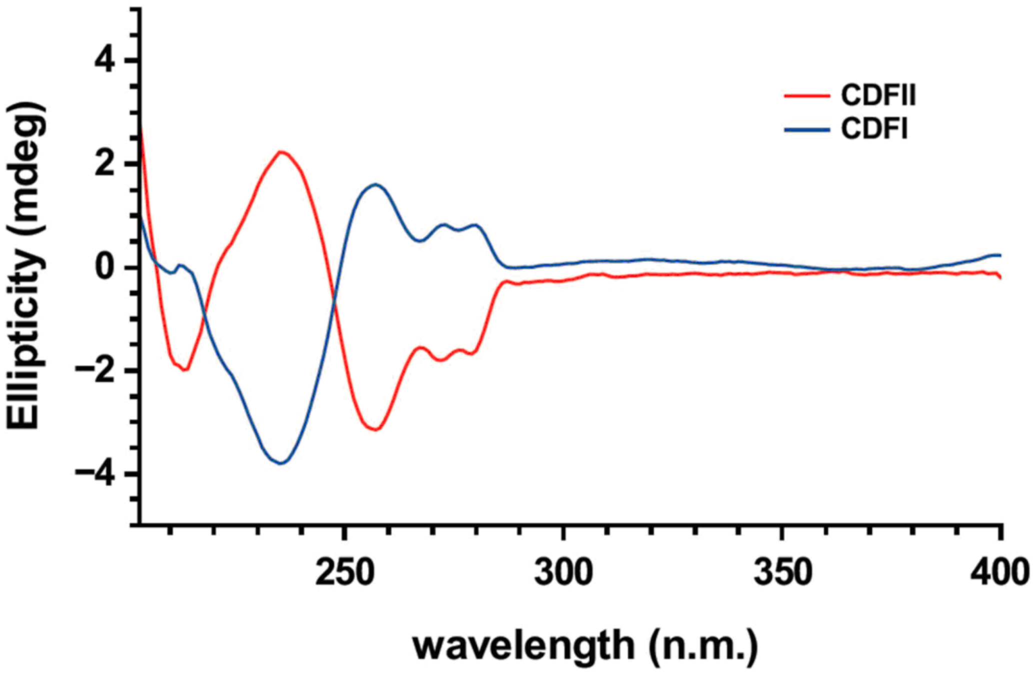

Figure S9 | The Circular Dichroism (CD) spectra collected for the *anti* enantiomeric pair display an intense induced CD signal. The first enantiomer isolated (blue) absorbs with a negative band in the aromatic absorption region (239 nm) and a positive band in the borazine region (272-279 nm) while the second enantiomer (red) displays opposite and symmetric CD maximum.

UV-Vis, Emission and Excitation spectra

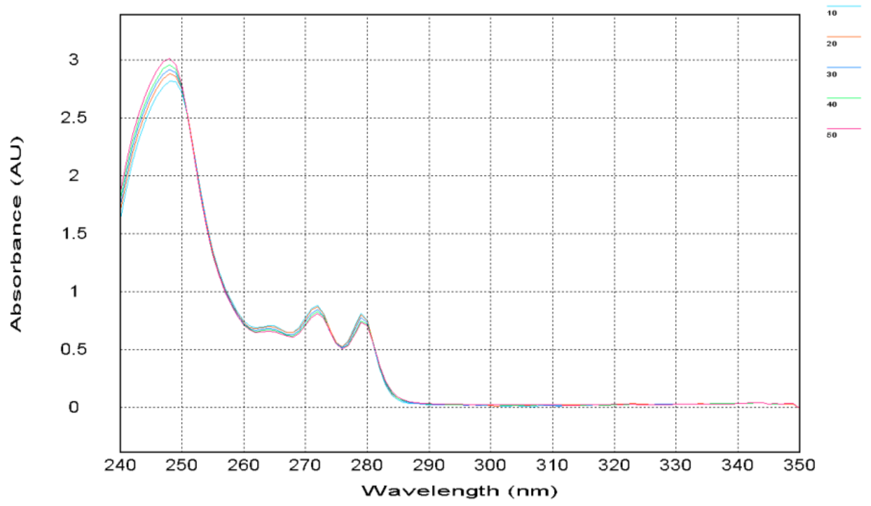

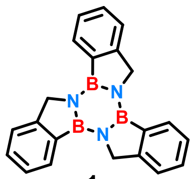

1

λ_{ex} (nm) = 279.5

λ_{em} (nm) = 294

ε (L mol^{-1} cm^{-1}) =10650

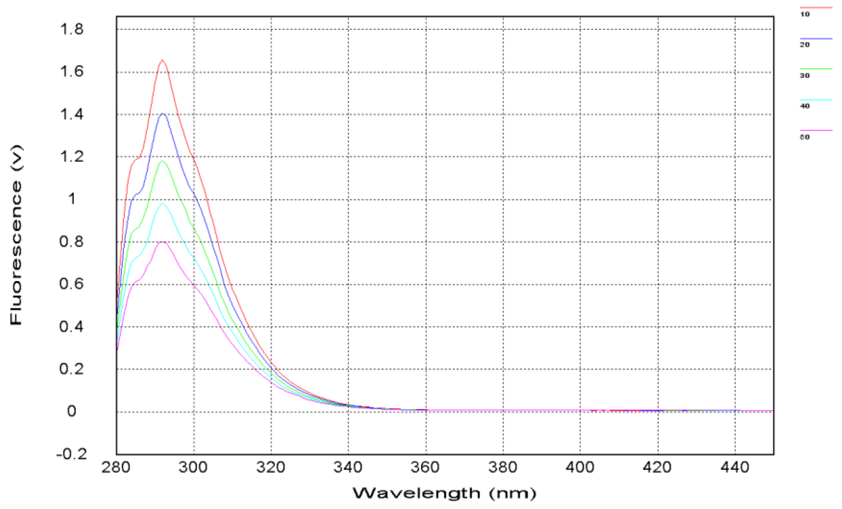

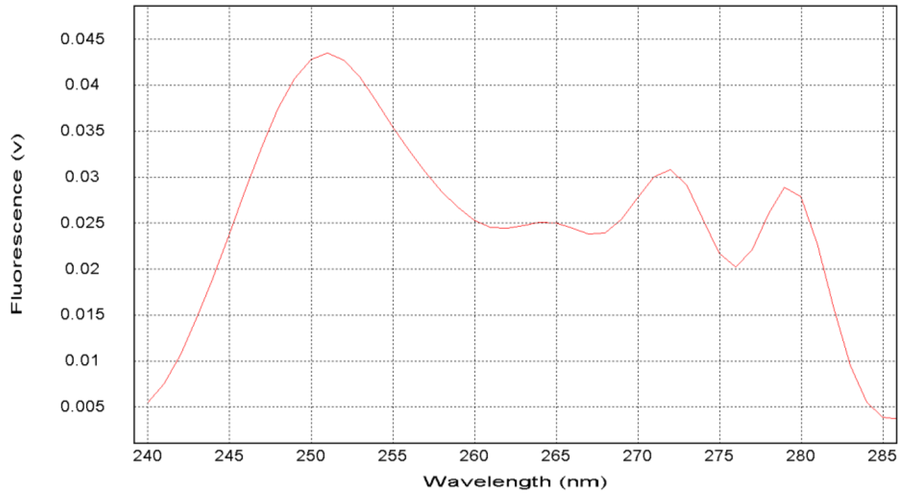

Figure S10 | Variable temperature absorption (top) and emission (middle) spectra, and excitation spectrum (bottom) of borazine **1** in $CHCl_3$

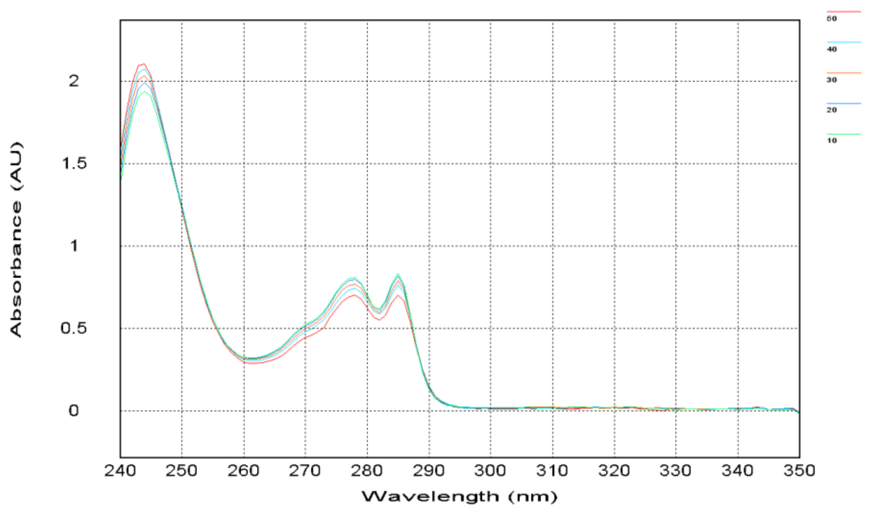

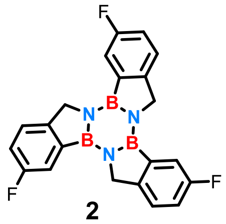

2

λ_{ex} (nm) = 285.5
λ_{em} (nm) = 301
ε (L mol^{-1} cm^{-1}) =10280

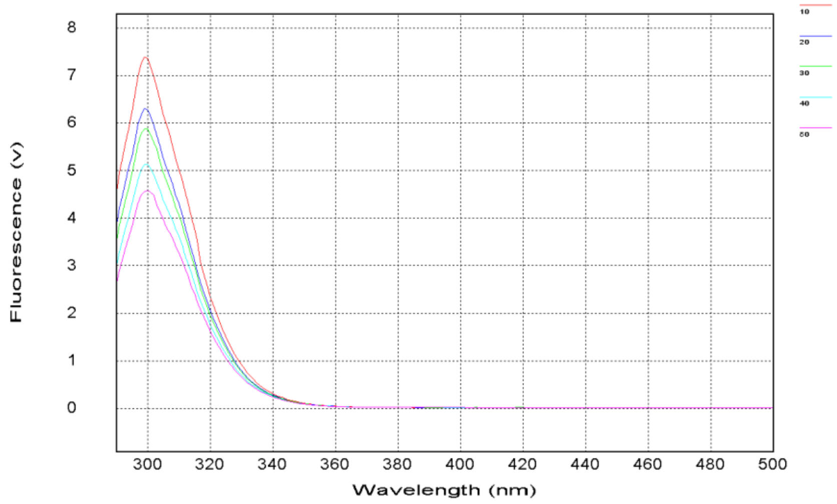

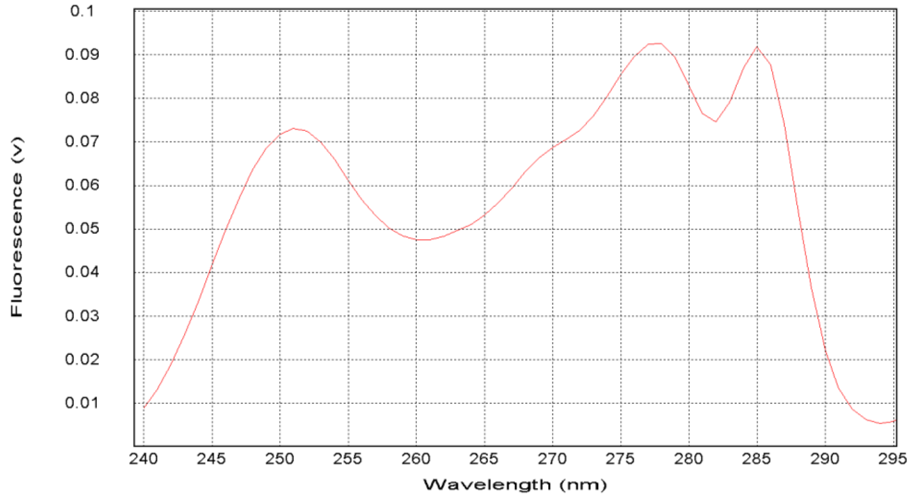

Figure S11 | Variable temperature absorption (top) and emission (middle) spectra, and excitation spectrum (bottom) of borazine **2** in $CHCl_3$

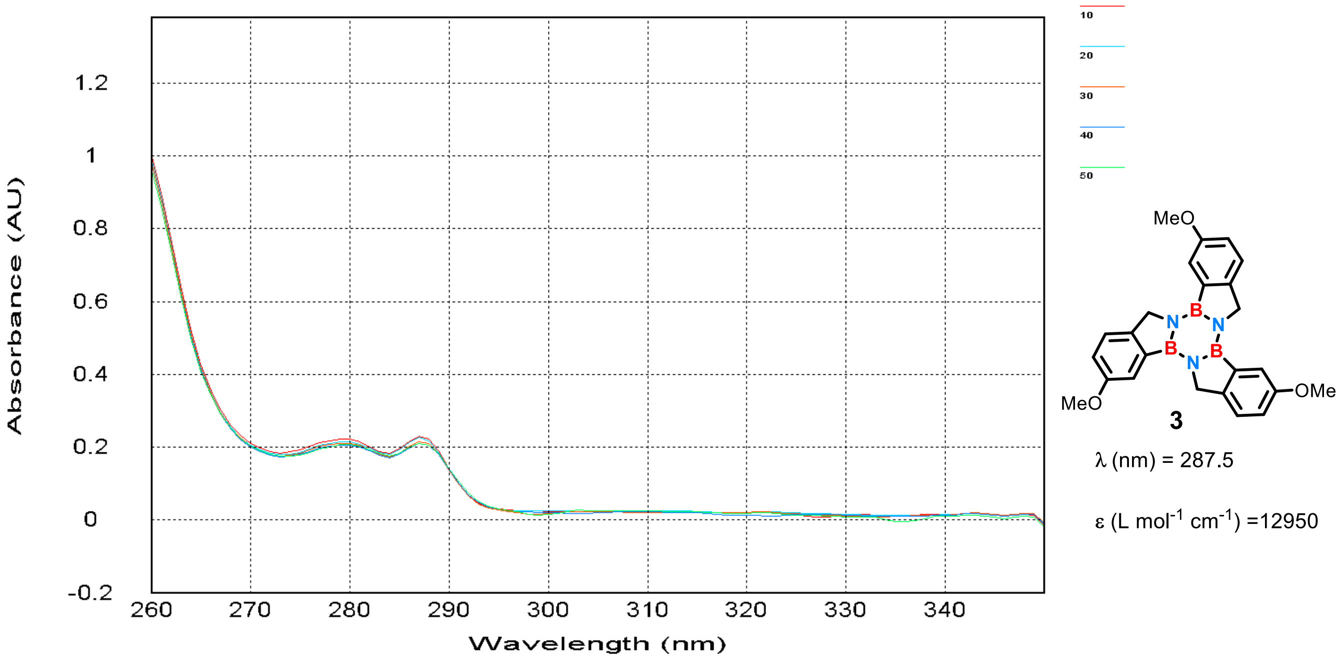

Figure S12 | Variable Temperature Absorption spectra of borazine **3** in $CHCl_3$

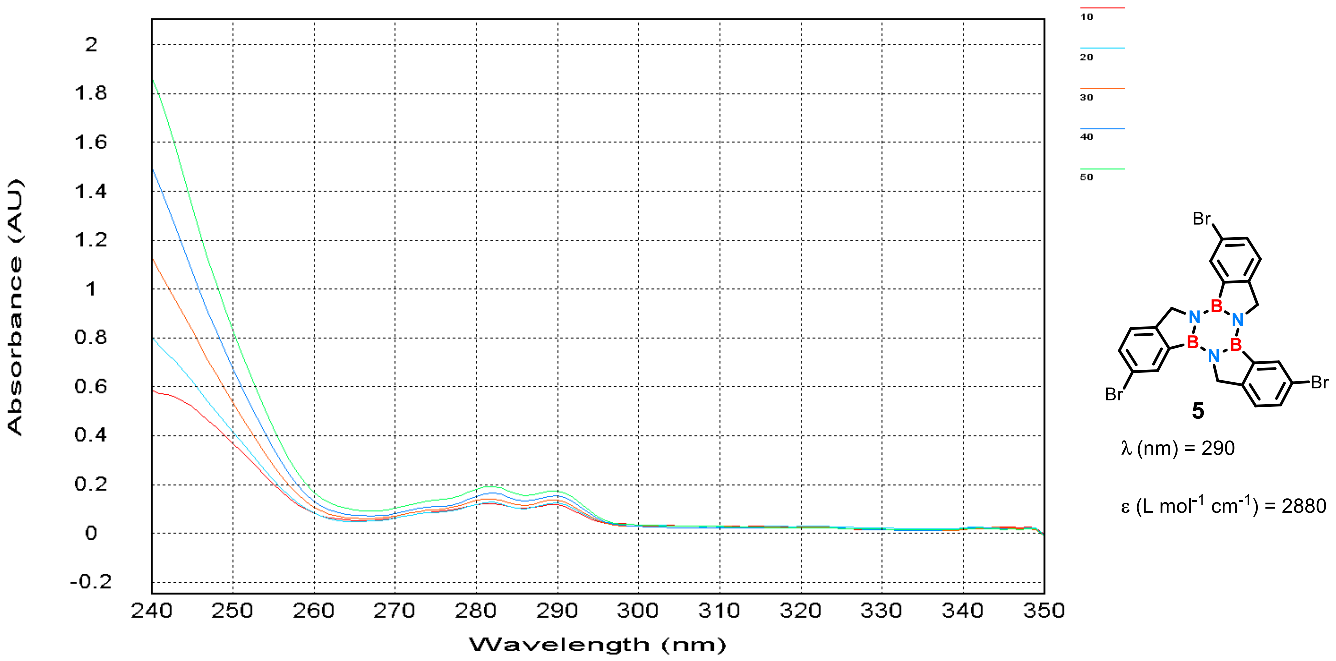

Figure S13 | Variable Temperature Absorption spectra of borazine **5** in $CHCl_3$

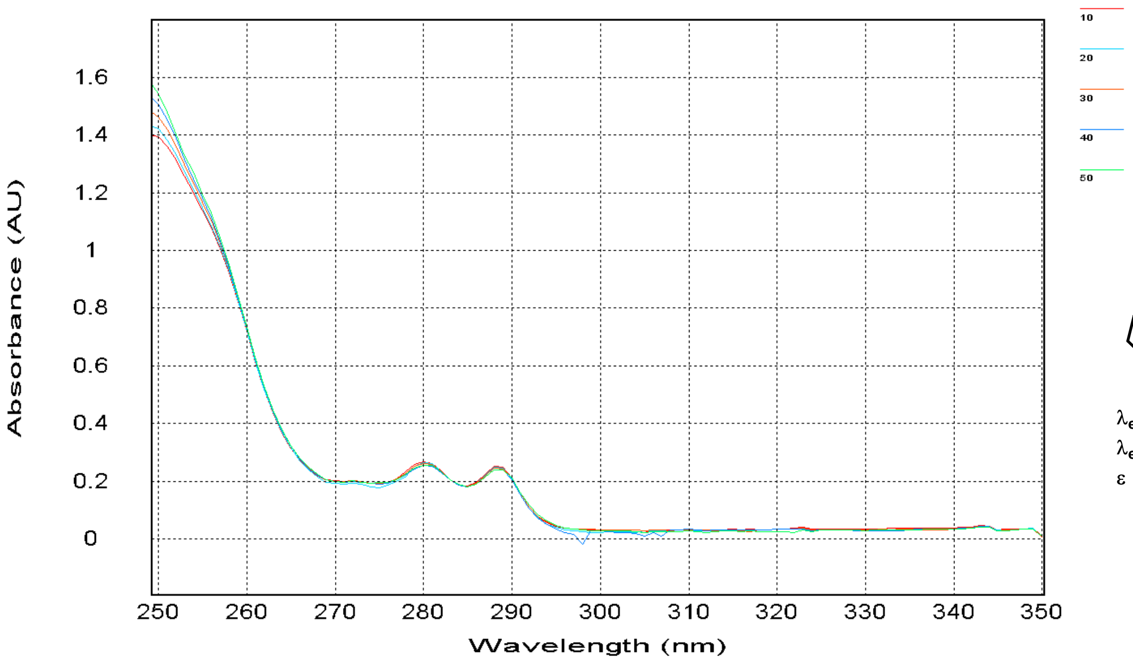

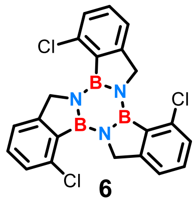

6

λ_{ex} (nm) = 289

λ_{em} (nm) = 298

ε (L mol^{-1} cm^{-1}) = 4410

Figure S14 | Variable Temperature Absorption spectra of borazine **6** in $CHCl_3$

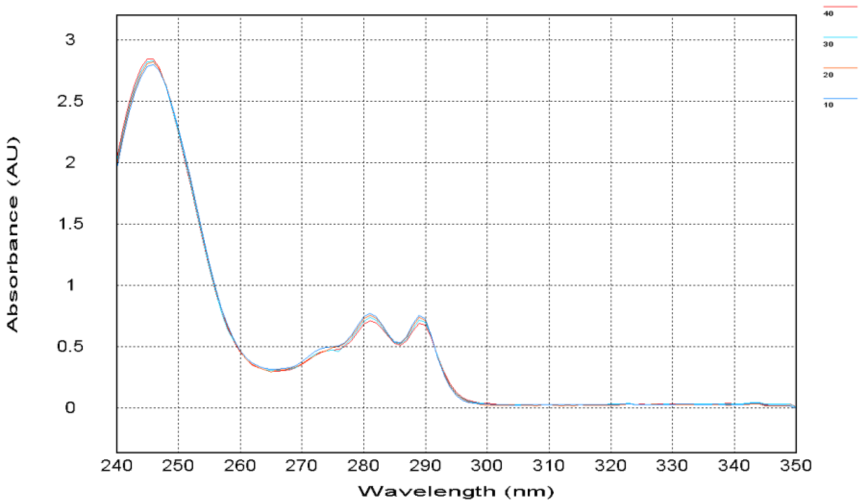

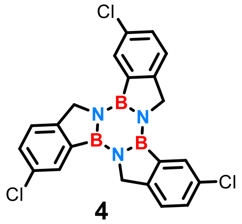

4

λ_{ex} (nm) = 290

λ_{em} (nm) = 305

ε (L mol^{-1} cm^{-1}) = 6740

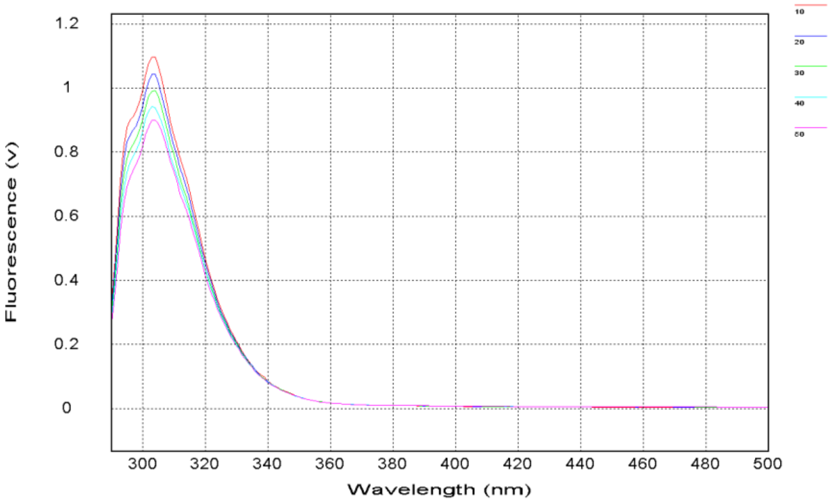

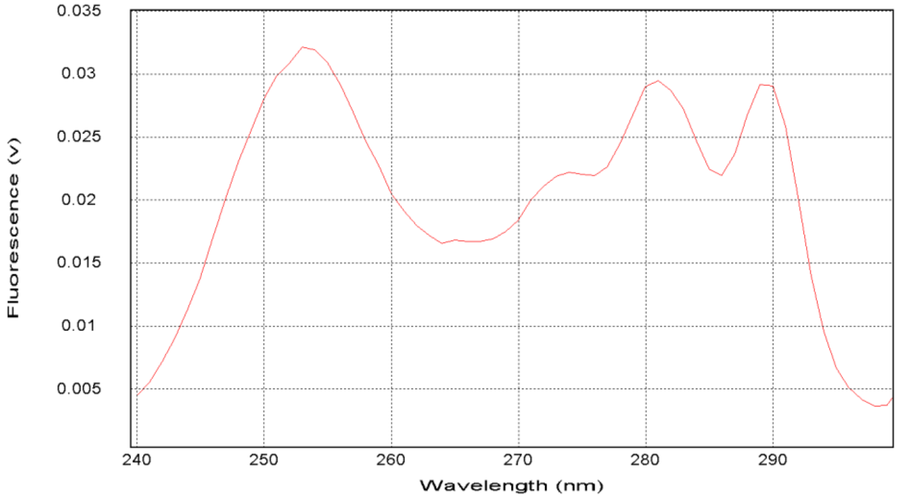

Figure S15 | Variable temperature absorption (top) and emission (middle) spectra, and excitation spectrum (bottom) of borazine **4** in $CHCl_3$

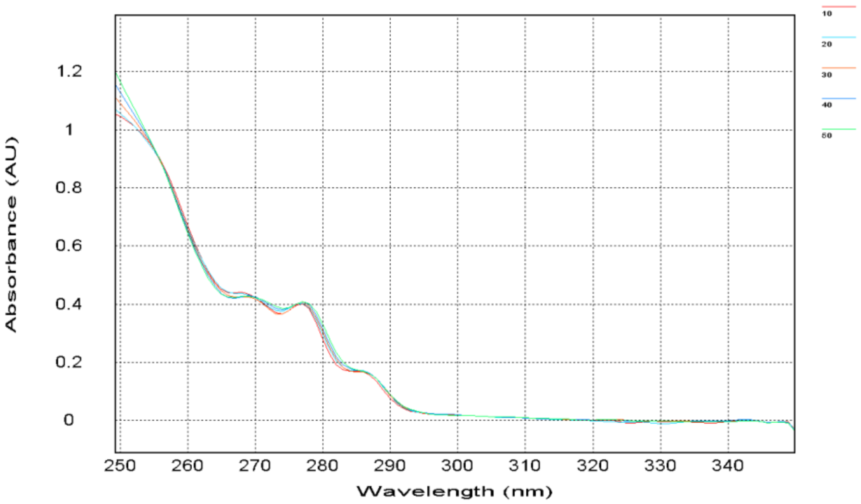

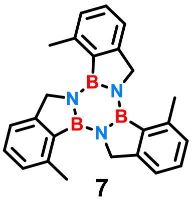

7

λ_{ex} (nm) = 278
λ_{em} (nm) = 301
ε (L mol^{-1} cm^{-1}) = 6563

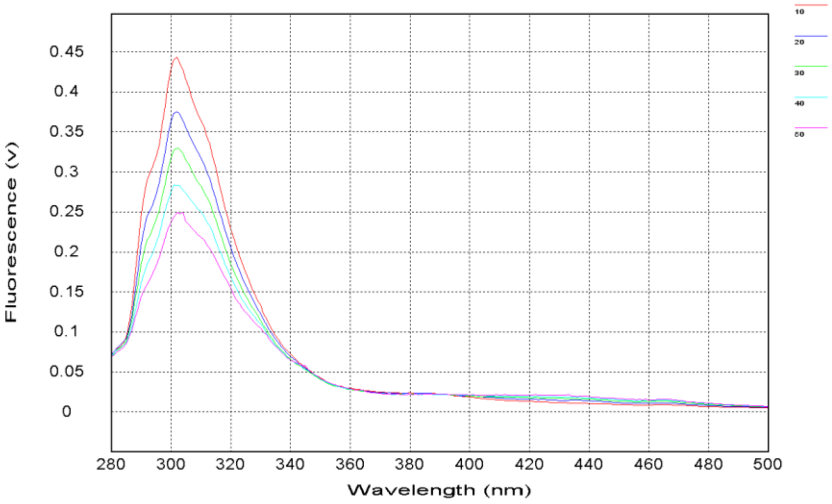

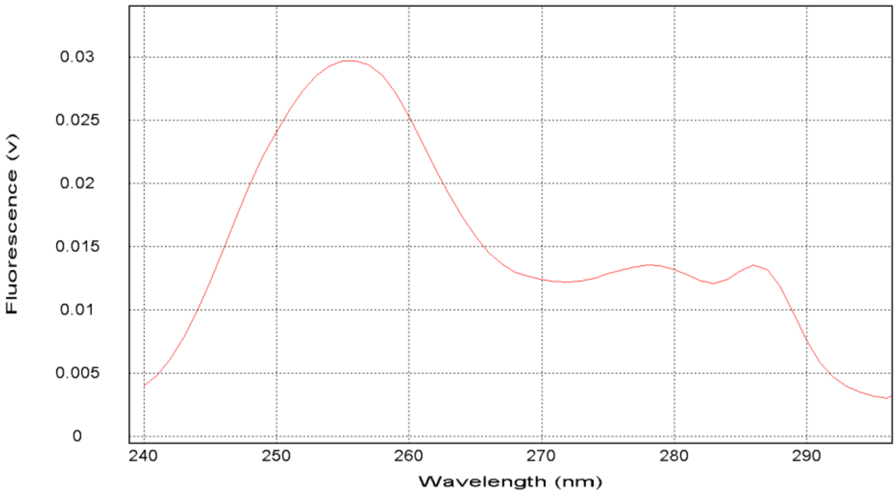

Figure S16| Variable temperature absorption (top) and emission (middle) spectra, and excitation spectrum (bottom) of borazine **7** in $CHCl_3$

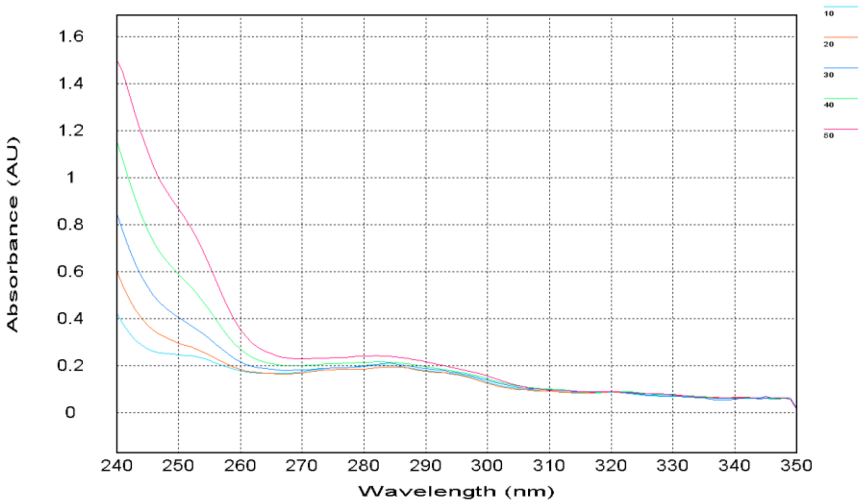

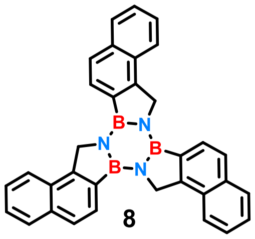

8

λ_{ex} (nm) = 298.5
λ_{em} (nm) = 344
ε (L mol^{-1} cm^{-1}) = 7930

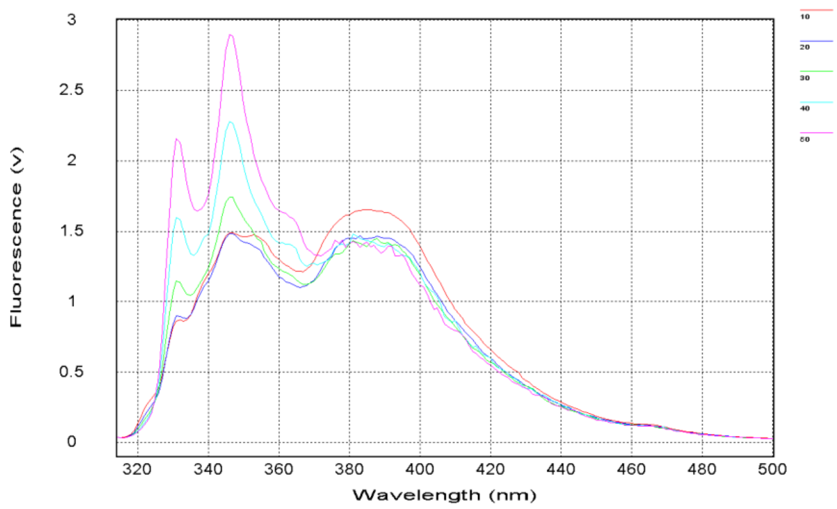

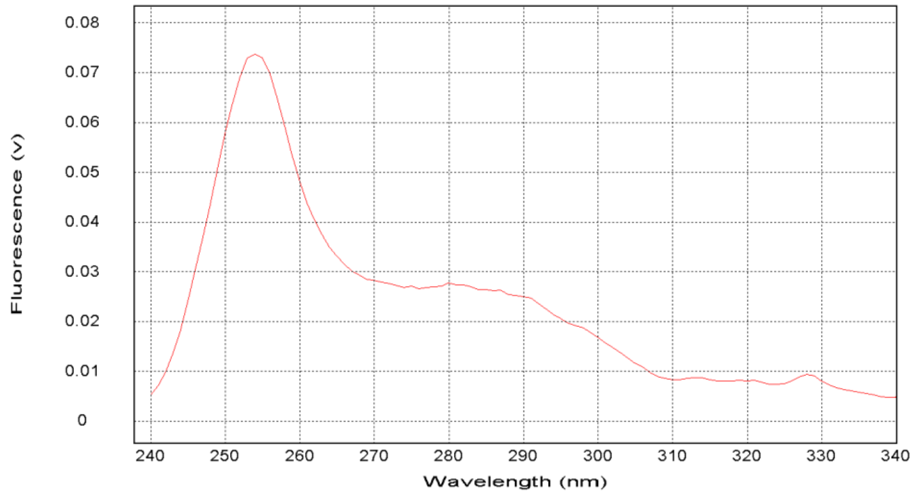

Figure S17 | Variable temperature absorption (top) and emission (middle) spectra, and excitation spectrum (bottom) of borazine **8** in $CHCl_3$

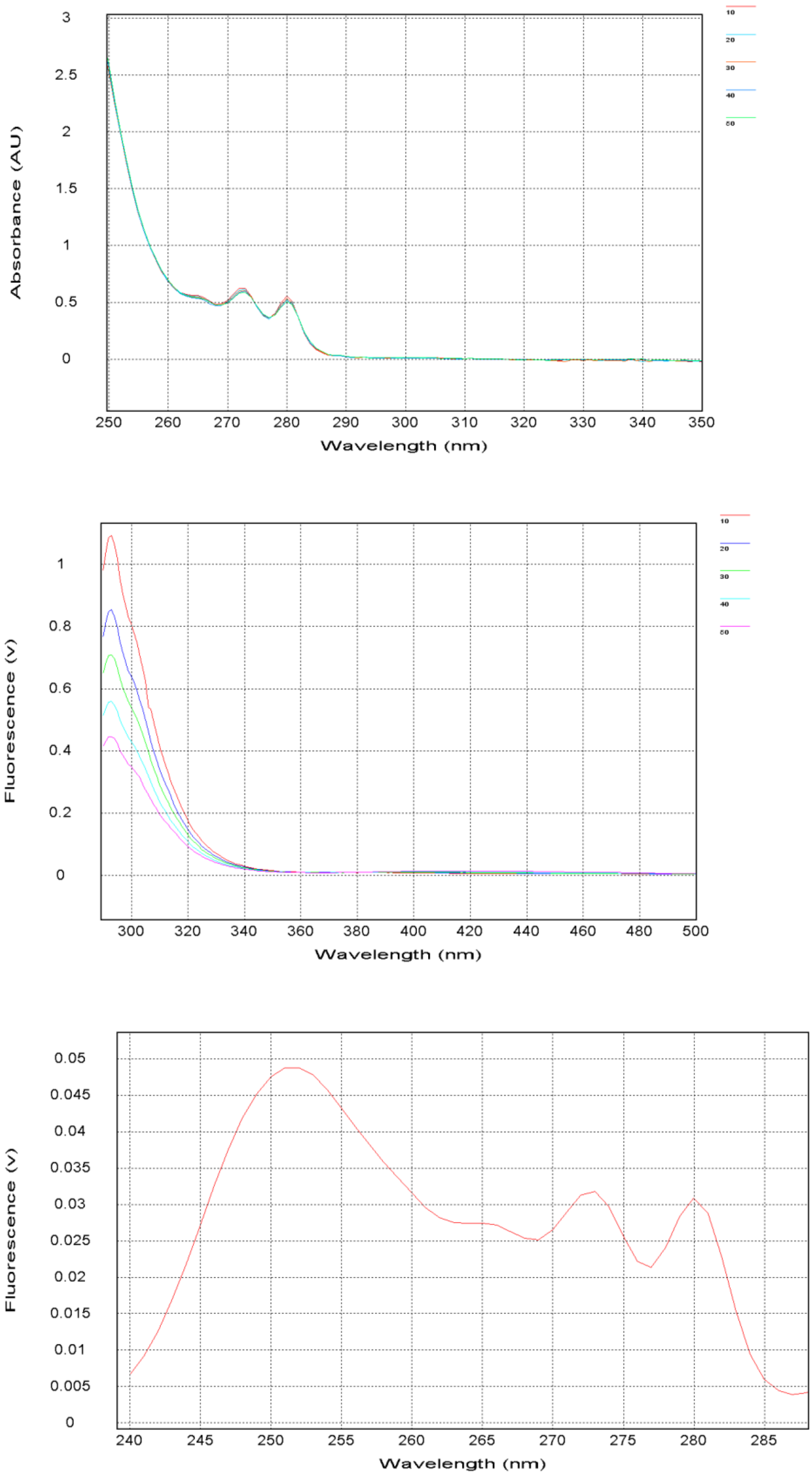

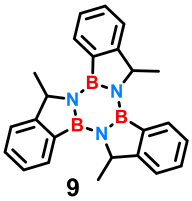

9

λ_{ex} (nm) = 272
λ_{em} (nm) = 293
ε (L mol^{-1} cm^{-1}) = 3177

Figure S18 | Variable temperature absorption (top) and emission (middle) spectra, and excitation spectrum (bottom) of borazine **9** in $CHCl_3$

X-ray data for compounds 11, 1 and 9-*syn*

Table S4. Crystal data and structure refinement for 11.

Identification code	11	
Empirical formula	C7 H10 B N	
Formula weight	118.97	
Temperature	149.99(11) K	
Wavelength	0.71073 Å	
Crystal system	Monoclinic	
Space group	$P2_1/n$	
Unit cell dimensions	a = 6.4509(3) Å	α= 90°.
	b = 10.8604(5) Å	β= 99.032(4)°.
	c = 9.7158(5) Å	γ = 90°.
Volume	672.24(6) $Å^3$	
Z	4	
Density (calculated)	1.175 Mg/m^3	
Absorption coefficient	0.067 mm^{-1}	
F(000)	256	
Crystal size	0.600 x 0.400 x 0.250 mm^3	
Theta range for data collection	3.550 to 30.215°.	
Index ranges	-8<=h<=8, -14<=k<=15, -12<=l<=13	
Reflections collected	6000	
Independent reflections	1776 [R(int) = 0.0219]	
Completeness to theta = 25.242°	99.9 %	
Absorption correction	Semi-empirical from equivalents	
Max. and min. transmission	1.00000 and 0.93214	
Refinement method	Full-matrix least-squares on F^2	
Data / restraints / parameters	1776 / 0 / 98	
Goodness-of-fit on F^2	1.048	
Final R indices [I>2sigma(I)]	R1 = 0.0455, wR2 = 0.1116	
R indices (all data)	R1 = 0.0572, wR2 = 0.1192	
Extinction coefficient	n/a	
Largest diff. peak and hole	0.339 and -0.197 e.$Å^{-3}$	

Table S5. Atomic coordinates (x 10^4) and equivalent isotropic displacement parameters (Å^{2}x 10^3) for **11**. U(eq) is defined as one third of the trace of the orthogonalized U^{ij} tensor.

	x	y	z	U(eq)
B	4751(2)	4819(1)	7529(1)	23(1)
N	6504(2)	5725(1)	7010(1)	22(1)
C(1)	8096(2)	4939(1)	6462(1)	24(1)
C(2)	6946(2)	3761(1)	6020(1)	19(1)
C(3)	7601(2)	2864(1)	5161(1)	23(1)
C(4)	6337(2)	1836(1)	4814(1)	26(1)
C(5)	4464(2)	1711(1)	5345(1)	28(1)
C(6)	3851(2)	2604(1)	6226(1)	24(1)
C(7)	5073(2)	3654(1)	6576(1)	19(1)

Table S6. Crystal data and structure refinement for 1.

Identification code	**1**	
Empirical formula	C21 H18 B3 N3	
Formula weight	344.81	
Temperature	150(2) K	
Wavelength	0.71073 Å	
Crystal system	Monoclinic	
Space group	P 2_1/c	
Unit cell dimensions	a = 5.13460(10) Å	[illegible]= 90°.
	b = 14.3862(3) Å	[illegible]= 91.5300(9)°.
	c = 23.4929(6) Å	[illegible] = 90°.
Volume	1734.74(7) $Å^3$	
Z	4	
Density (calculated)	1.320 Mg/m^3	
Absorption coefficient	0.076 mm^{-1}	
F(000)	720	
Crystal size	0.600 x 0.150 x 0.080 mm^3	
Theta range for data collection	2.962 to 27.487°.	
Index ranges	-6<=h<=6, -18<=k<=18, -30<=l<=30	
Reflections collected	22750	
Independent reflections	3982 [R(int) = 0.0929]	
Completeness to theta = 25.242°	99.7 %	
Absorption correction	Semi-empirical from equivalents	
Max. and min. transmission	1.003 and 0.793	
Refinement method	Full-matrix least-squares on F^2	
Data / restraints / parameters	3982 / 0 / 244	
Goodness-of-fit on F^2	1.009	
Final R indices [I>2sigma(I)]	R1 = 0.0525, wR2 = 0.1093	
R indices (all data)	R1 = 0.1074, wR2 = 0.1289	
Extinction coefficient	n/a	
Largest diff. peak and hole	0.215 and -0.231 e.$Å^{-3}$	

Table S7. Atomic coordinates (x 10^4) and equivalent isotropic displacement parameters ($Å^2$x 10^3) for **1**. U(eq) is defined as one third of the trace of the orthogonalized U^{ij} tensor.

	x	y	z	U(eq)
N(1)	-1532(2)	5164(1)	2571(1)	27(1)
N(2)	1267(3)	4021(1)	2108(1)	27(1)
N(3)	-1916(3)	4922(1)	1536(1)	28(1)
B(1)	558(4)	4494(1)	2612(1)	27(1)
B(2)	89(4)	4246(1)	1558(1)	27(1)
B(3)	-2782(4)	5383(1)	2042(1)	27(1)
C(1)	-1880(3)	5624(1)	3123(1)	30(1)
C(2)	76(3)	5161(1)	3522(1)	28(1)
C(3)	481(3)	5347(1)	4097(1)	34(1)
C(4)	2370(3)	4845(1)	4399(1)	37(1)
C(5)	3851(3)	4172(1)	4130(1)	34(1)
C(6)	3444(3)	3989(1)	3557(1)	30(1)
C(7)	1554(3)	4486(1)	3244(1)	27(1)
C(8)	3360(3)	3334(1)	2050(1)	29(1)
C(9)	3373(3)	3121(1)	1418(1)	28(1)
C(10)	5026(3)	2509(1)	1146(1)	33(1)
C(11)	4739(4)	2398(1)	560(1)	37(1)
C(12)	2847(4)	2891(1)	252(1)	37(1)
C(13)	1232(3)	3509(1)	525(1)	34(1)
C(14)	1480(3)	3633(1)	1114(1)	28(1)
C(15)	-3317(3)	5283(1)	1027(1)	30(1)
C(16)	-5209(3)	5984(1)	1254(1)	28(1)
C(17)	-6989(3)	6520(1)	941(1)	34(1)
C(18)	-8611(3)	7121(1)	1227(1)	35(1)
C(19)	-8505(3)	7175(1)	1814(1)	33(1)
C(20)	-6734(3)	6650(1)	2126(1)	30(1)
C(21)	-5017(3)	6056(1)	1850(1)	28(1)

Table S8. Crystal data and structure refinement for borazine **9-syn**.

Identification code	**9-syn**	
Empirical formula	C24 H24 B3 N3	
Formula weight	386.89	
Temperature	150.01(10) K	
Wavelength	1.54184 Å	
Crystal system	Trigonal	
Space group	R-3	
Unit cell dimensions	a = 20.4365(5) Å	α= 90°.
	b = 20.4365(5) Å	β= 90°.
	c = 8.6540(2) Å	γ = 120°.
Volume	3130.12(17) Å^3	
Z	6	
Density (calculated)	1.231 Mg/m^3	
Absorption coefficient	0.540 mm^{-1}	
F(000)	1224	
Crystal size	0.150 x 0.150 x 0.120 mm^3	
Theta range for data collection	4.327 to 72.930°.	
Index ranges	-24<=h<=25, -25<=k<=24, -6<=l<=10	
Reflections collected	7994	
Independent reflections	1383 [R(int) = 0.0400]	
Completeness to theta = 67.684°	100.0 %	
Absorption correction	Semi-empirical from equivalents	
Max. and min. transmission	1.00000 and 0.88943	
Refinement method	Full-matrix least-squares on F^2	
Data / restraints / parameters	1383 / 0 / 154	
Goodness-of-fit on F^2	1.074	
Final R indices [I>2sigma(I)]	R1 = 0.0366, wR2 = 0.0963	
R indices (all data)	R1 = 0.0411, wR2 = 0.0995	
Extinction coefficient	0.00048(8)	
Largest diff. peak and hole	0.156 and -0.134 e.Å^{-3}	

Table S9. Atomic coordinates (x 10^4) and equivalent isotropic displacement parameters ($Å^2$x 10^3) for borazine **9-syn**. U(eq) is defined as one third of the trace of the orthogonalized U^{ij} tensor.

	x	y	z	U(eq)
B(1)	3049(2)	5868(3)	4523(4)	23(1)
N(1)	3854(1)	6396(1)	4478(2)	24(1)
C(1)	2971(1)	5064(1)	4682(2)	26(1)
C(2)	2364(1)	4335(1)	4860(2)	32(1)
C(3)	2492(1)	3734(1)	5072(3)	42(1)
C(4)	3226(1)	3861(1)	5108(3)	39(1)
C(5)	3833(1)	4590(1)	4930(2)	34(1)
C(6)	3706(1)	5191(1)	4718(2)	27(1)
C(7)	4298(1)	6011(1)	4534(2)	26(1)
C(8)	4770(2)	6148(2)	3074(3)	32(1)
B(2)	3351(2)	5973(2)	4527(4)	23(1)
N(2)	2641(1)	5968(1)	4483(2)	24(1)
C(21)	3135(1)	5124(1)	4697(2)	28(1)
C(22)	2350(1)	4712(1)	4676(2)	29(1)
C(23)	1977(1)	3931(1)	4846(2)	38(1)
C(24)	2388(1)	3563(1)	5036(3)	44(1)
C(25)	3173(1)	3976(1)	5057(3)	42(1)
C(26)	3547(1)	4756(1)	4887(2)	32(1)
C(27)	1989(1)	5194(1)	4509(2)	26(1)
C(28)	1514(2)	5012(1)	3038(3)	33(1)

Molecular Modelling data

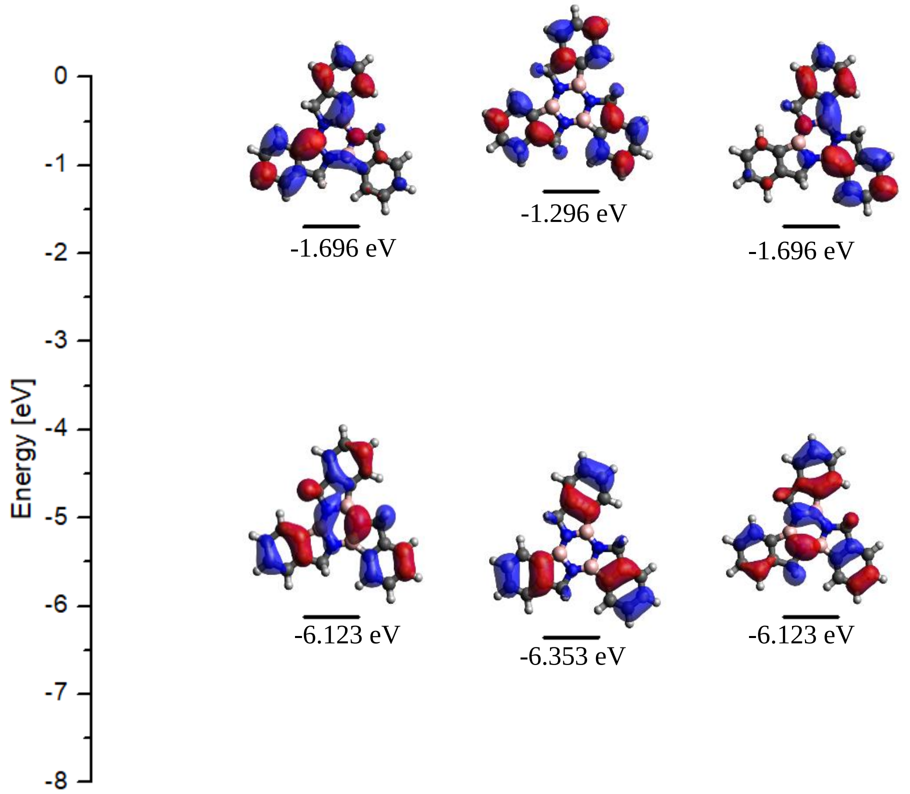

Figure S19 | Energy levels diagram and frontier orbitals of borazatruxene **1** resulted from M11-L/6-311G(d,p) geometry optimization

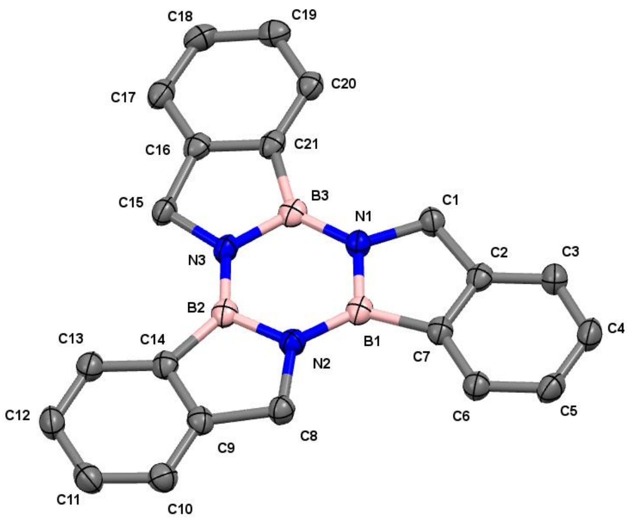

Table S10. a) Average bond distance of B1-N2, B2-N3 and B3-N1 in Å; b) Average bond distance of N1-B1, N2-B2 and N3-B3 in Å; c) Average bond angle of B1-N2-B2, B2-N3-B3, B3-N1-B1 in degrees; d) Average bond angle of N1-B1-N2, N2-B2-N3 and N3-B3-N1 in degrees; e) Average bond distance of C1-N1-B1, C8-N2-B2 and C15-N3-B3 in degrees; f) Average bond distance of N1-B1-C7, N2-B2-C14 and N3-B3-C21 in degrees. Numbering according the the figure above.

	B-N[a] bond [Å]	B-N[b] bond [Å]	B-N-B[c] angle [°]	N-B-N[d] angle [°]	C-N-B[e] angle [°]	N-B-C[f] angle [°]
X-Ray	1.419	1.445	121.7	118.2	110.6	106.7
B3LYP/6-31G	1.425	1.458	121.7	118.3	110.9	106.5
B3LYP/6-31G(d.p)	1.423	1.449	121.5	118.5	110.8	106.8
B3LYP/6-311G	1.422	1.454	121.7	118.3	110.8	106.6
B3LYP/6-311G(d.p)	1.423	1.448	121.5	118.5	110.8	106.7
M11/6-31G	1.421	1.452	121.1	118.8	111.3	106.2
M11/6-31G(d.p)	1.421	1.445	119.1	119.1	111.2	106.5
M11/6-311G	1.419	1.450	118.8	118.9	111.2	106.3
M11/6-311G(d.p)	1.420	1.444	119.1	119.1	111.3	106.4
PM7	1.389	1.477	120.4	119.6	110.4	106.3
M06-2X/6-31G	1.420	1.453	118.6	118.6	111.3	106.3
M06-2X/6-31G(d.p)	1.420	1.445	121.2	118.8	111.1	106.5
M06-2X/6-311G	1.418	1.449	121.4	118.6	111.2	106.4
M06-2X/6-311G(d.p)	1.419	1.443	121.2	118.8	111.2	106.5
M11-L/6-31G	1.411	1.439	121.8	118.2	111.5	106.2
M11-L/6-311G	1.406	1.434	121.8	118.2	111.4	106.4
M11-L/6-31G(d.p)	1.409	1.429	121.5	118.5	111.4	106.5
M11-L/6-311G(d.p)	1.404	1.426	121.5	118.5	111.3	106.5

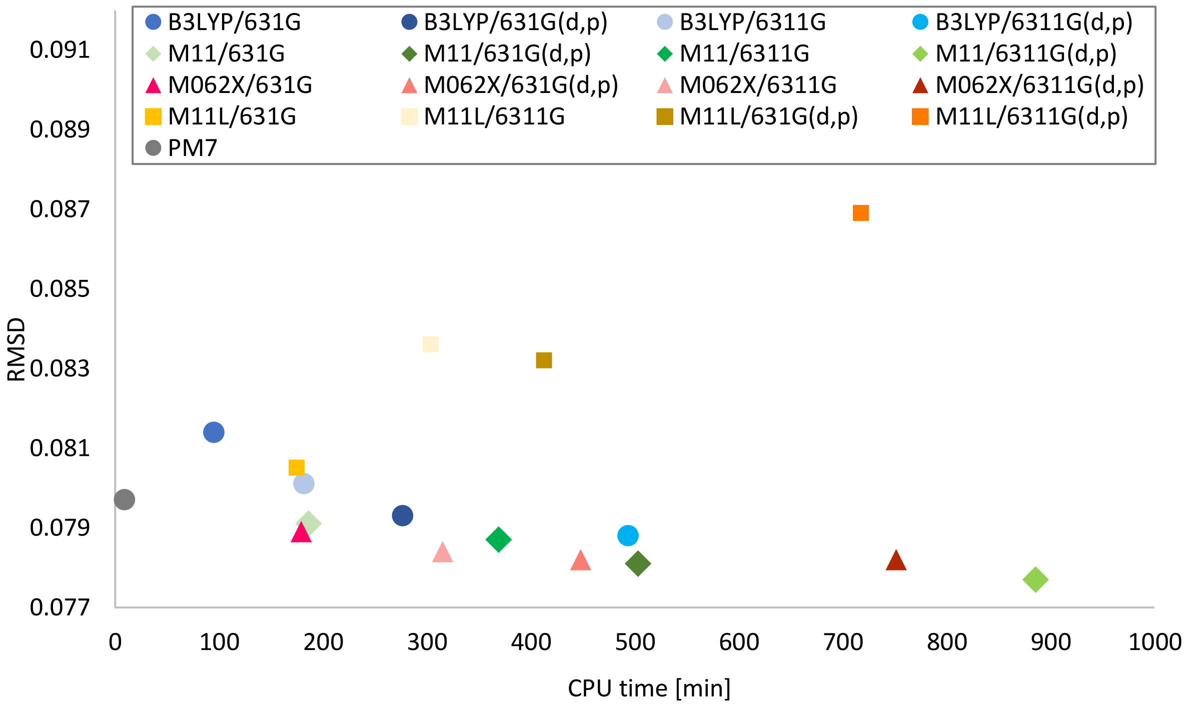

Figure S20 | Root mean squared deviation versus CPU time for various functionals and basis sets used for the geometry optimization of borazatruxene **1**

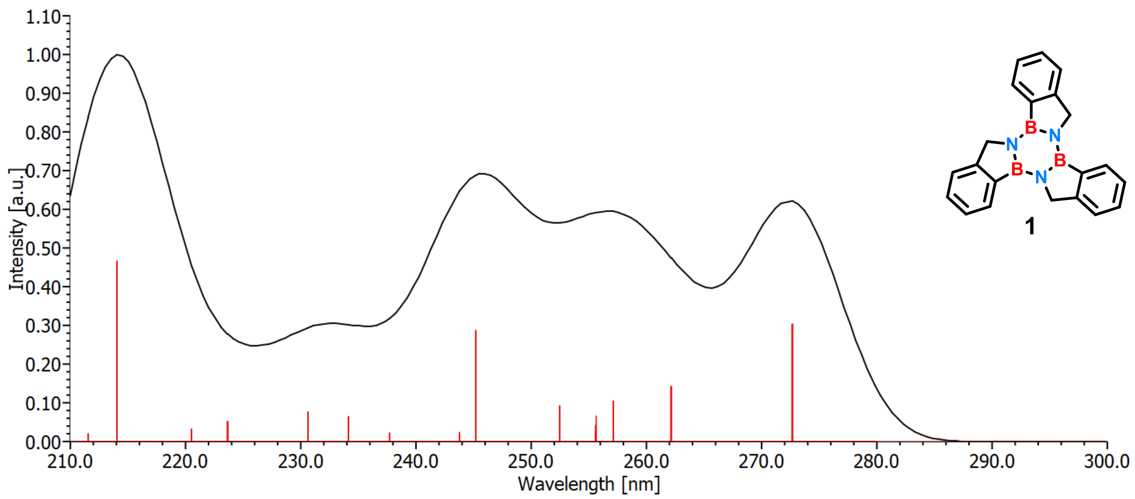

Figure S21| TD-DFT M11-L/6-311G(d,p) simulated UV-Vis spectra of borazine **1** in $CHCl_3$

Table S11 M11-L ($CHCl_3$) TD-DFT UV-Vis calculations of borazatruxenes 1-8 (excluding 4).

	Excitation (Experimental values) nm			Oscillator Strength (f)		
	1	2	3	1	2	3
Truxene	301.8 (298.5)	294.6 (290.5)	286.0 (275.5)	0.199	0.024	0.004
H	272.7 (279.5)	262.2 (272.0)	257.1 (265.0)	0.050	0.032	0.037
F	288.4 (285.5)	275.3 (278.5)	266.1 (271.0)	0.009	0.071	0.055
OMe	292.7 (287.5)	286.3 (280.5)	272.2 (277.5)	0.002	0.028	0.148
4-Cl	292.4 (289.5)	279.9 (281.0)	267.3 (275.5)	0.004	0.058	0.067
Br	294.4 (290.0)	282.3 (281.5)	267.8 (274.5)	0.003	0.051	0.068
3-Cl	289.1 (289.0)	279.2 (281.0)	267.9 (272.5)	0.017	0.049	0.016
Nap	358.4 (292.5)	357.3 (284.0)	312.2 (275.0)	0.06	0.007	0.03

AICD calculations have been performed for the M06-2X/6-311G optimized geometries of borazatruxene **1** and truxene using the IOP(10/93=1) option implemented in *Gaussian 16*.[6] Isosurface plots have been obtained using the AICD package[7] kindly provided by Dr. Rainer Herges group.

AICD isosurfaces are plotted in Figure S22, depicting the density of delocalized electrons for borazatruxene **1** as well as its organic counterpart, truxene. Current density vectors are plotted on top of the isosurface to illustrate its magnitude, as well as the diatropic or paratropic ring currents.

Both molecules exhibit strong delocalized electron density and paratropic currents in the arene region, with a small diatropic component inside these rings.

For truxene, delocalized electrons extend from the molecular core through two of the sp^2 carbon atoms of the five-membered ring to the lobes of the molecule, while in the case of borazatruxene **1** the boron atoms of the inorganic core break the delocalized electron density to the central core, therefore increasing its HOMO-LUMO gap.

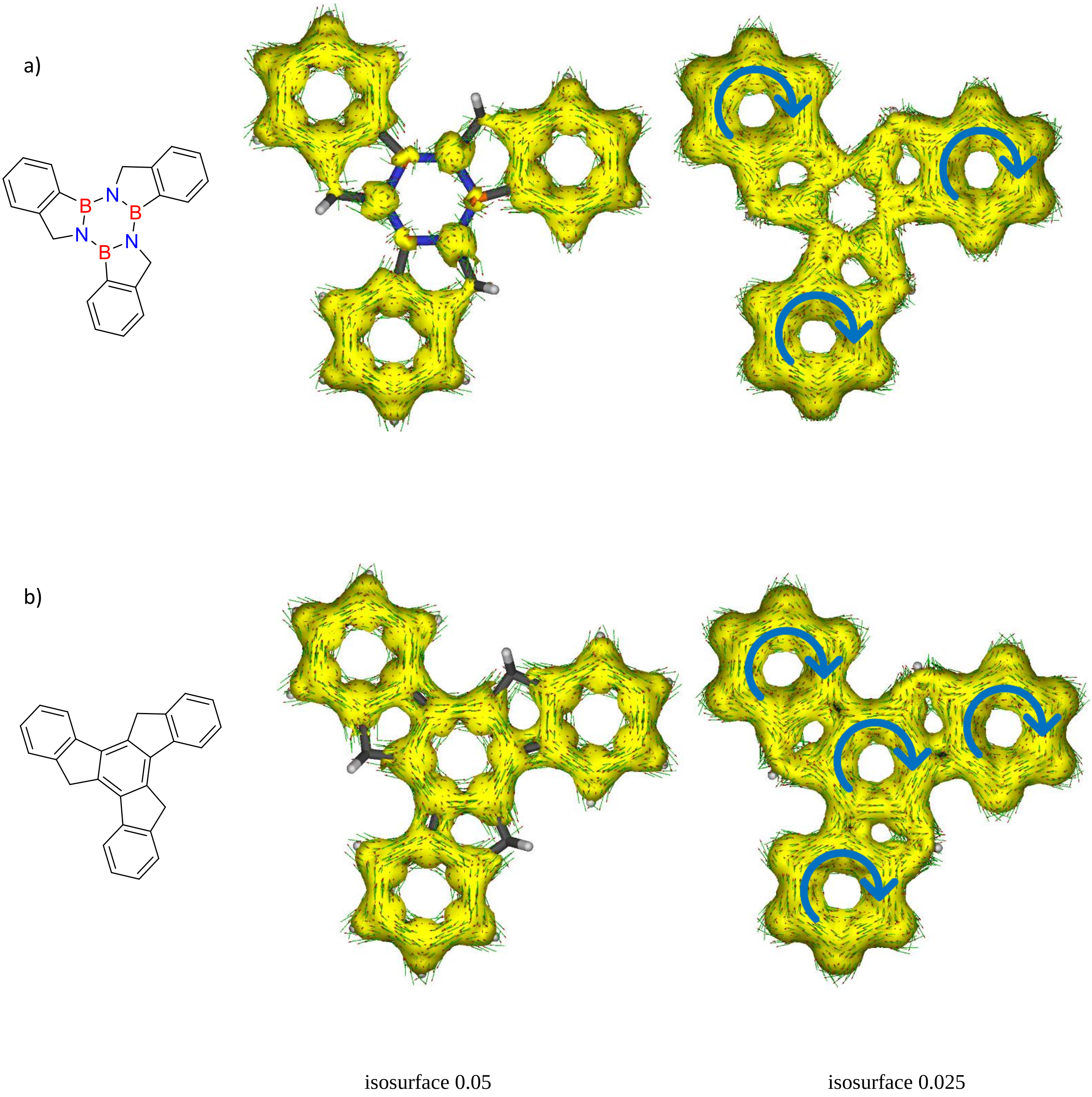

Figure S22. Anisotropy of the induced current density (AICD) isosurfaces and current density vectors of a) borazatruxene **1** and b) truxene. Magnetic field is perpendicular on the molecular plane and diatropic ring currents are turning clockwise.

NMR spectra

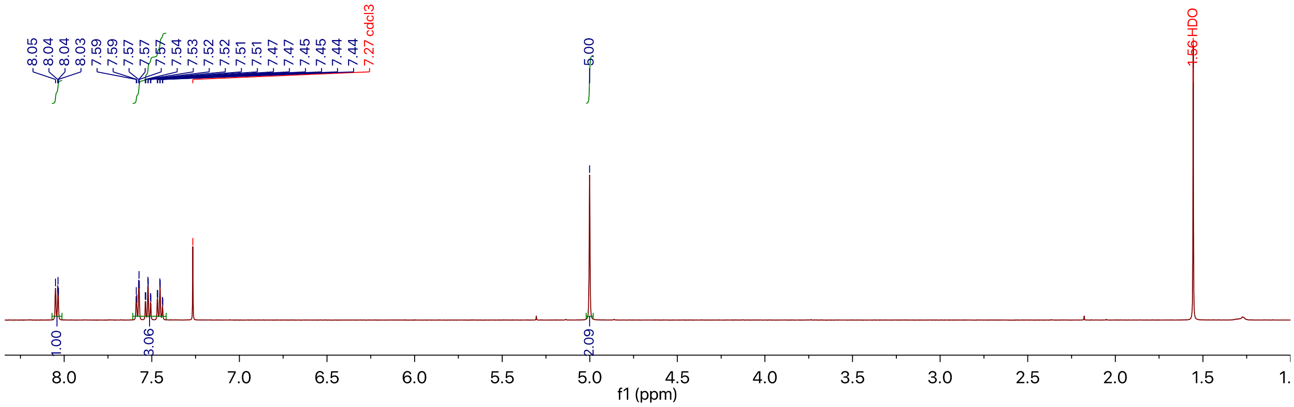

Figure S23. ^{1}H NMR spectrum of **1** ($CDCl_3$, 21 °C)

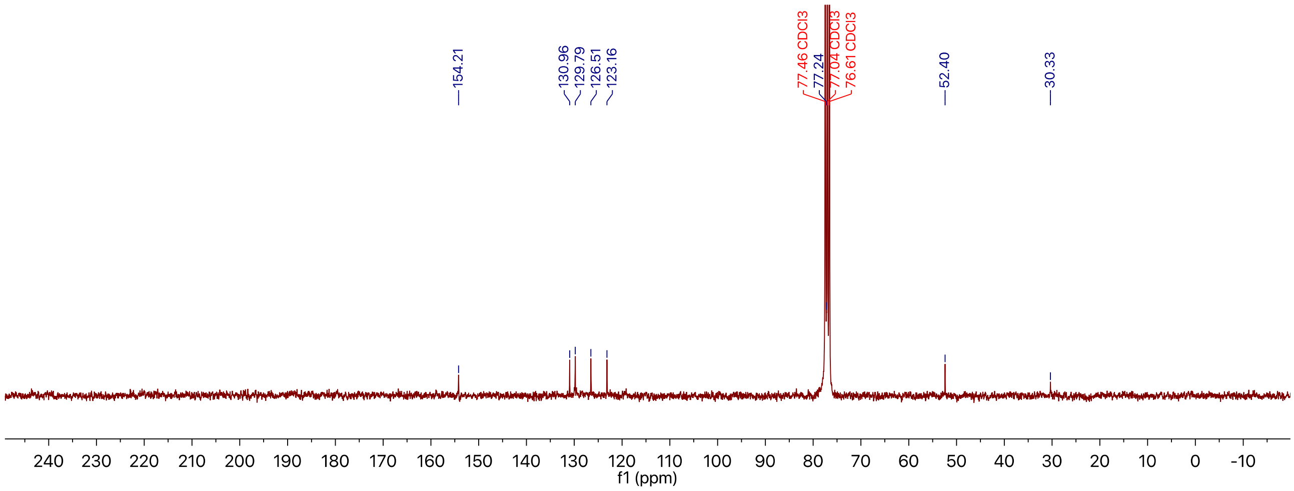

Figure S24. ^{13}C NMR spectrum of **1** ($CDCl_3$, 21 °C)

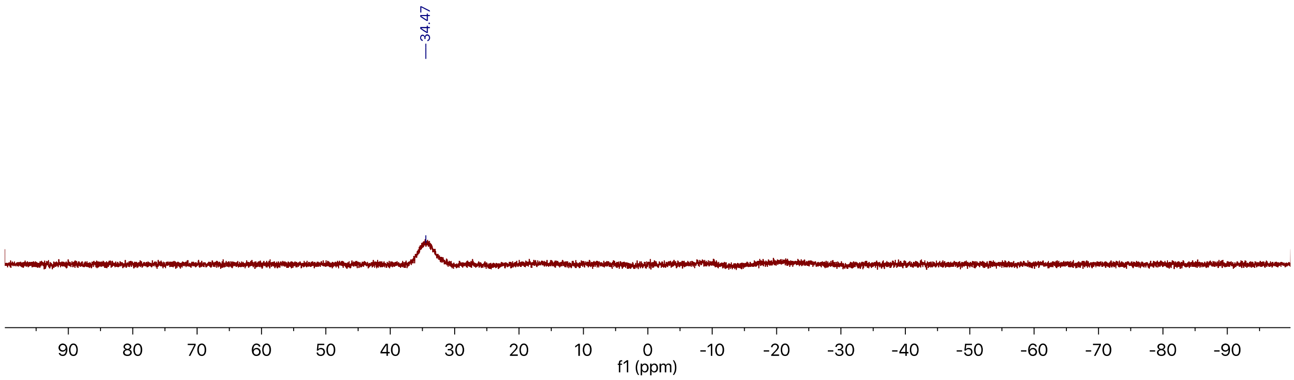

Figure S25. ^{11}B NMR spectrum of **1** ($CDCl_3$, 21 °C)

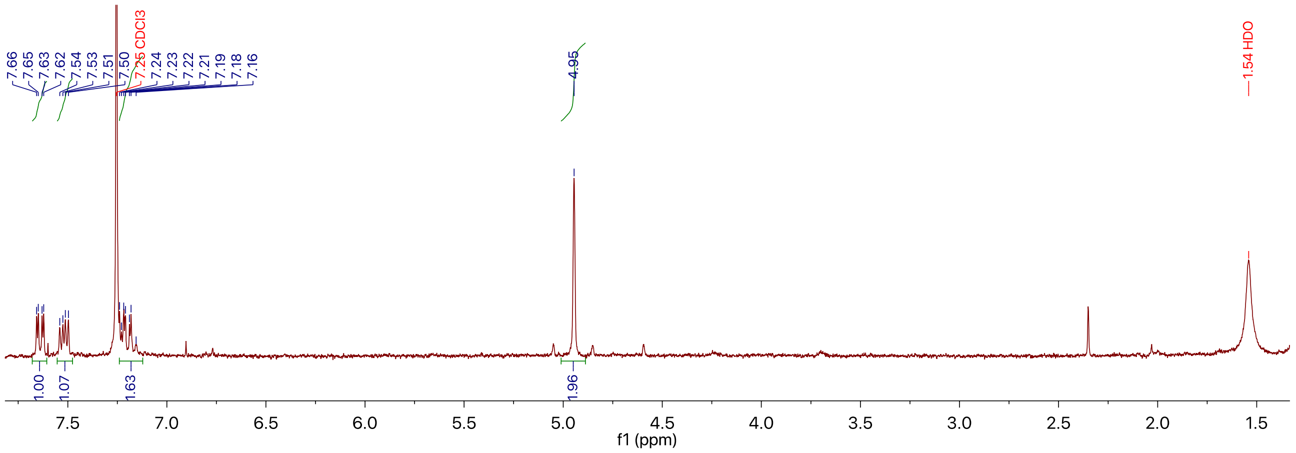

Figure S26. ^{1}H NMR spectrum of **2** ($CDCl_3$, 21 °C)

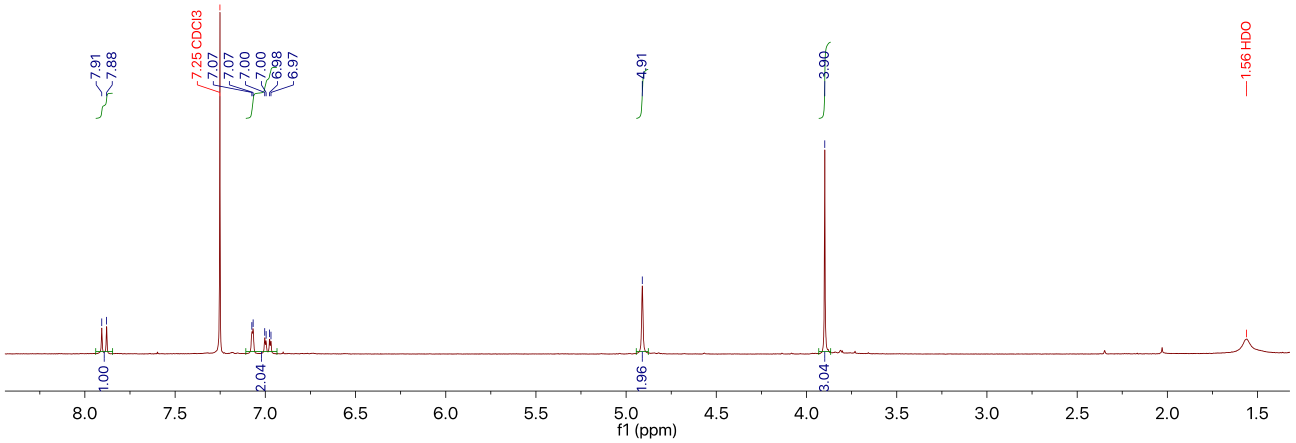

Figure S27. ^{1}H NMR spectrum of **3** ($CDCl_3$, 21 °C)

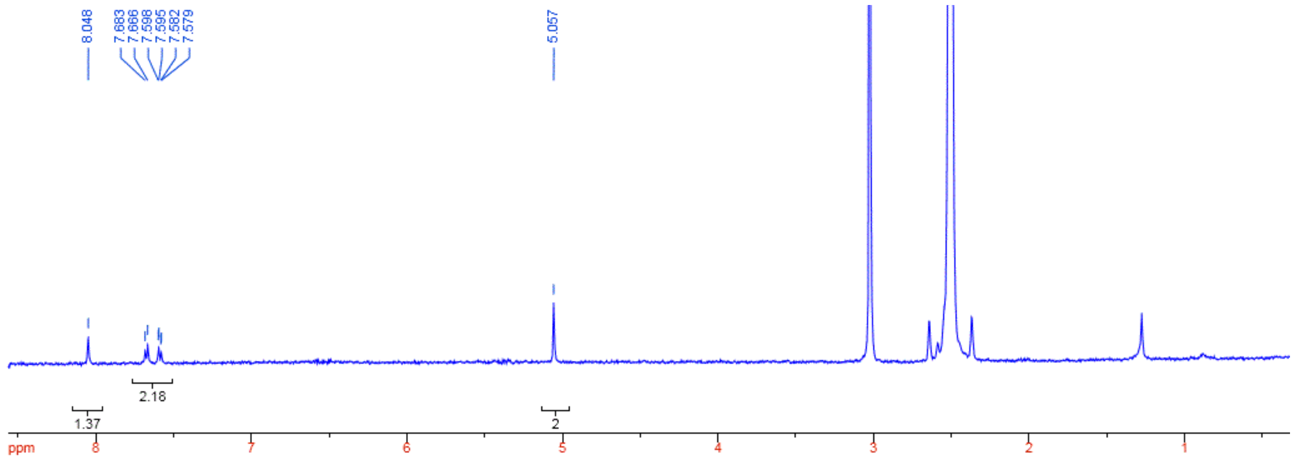

Figure S28. ^{1}H NMR spectrum of **4** (DMSO-d_6, 85 °C)

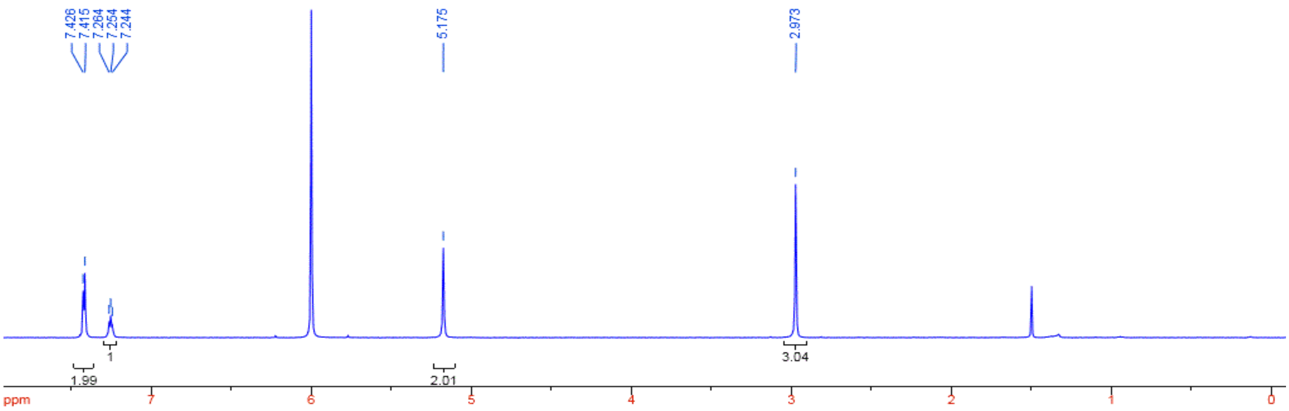

Figure S29. ^{1}H NMR spectrum of **7** (CD_2Cl_4, 75 °C)

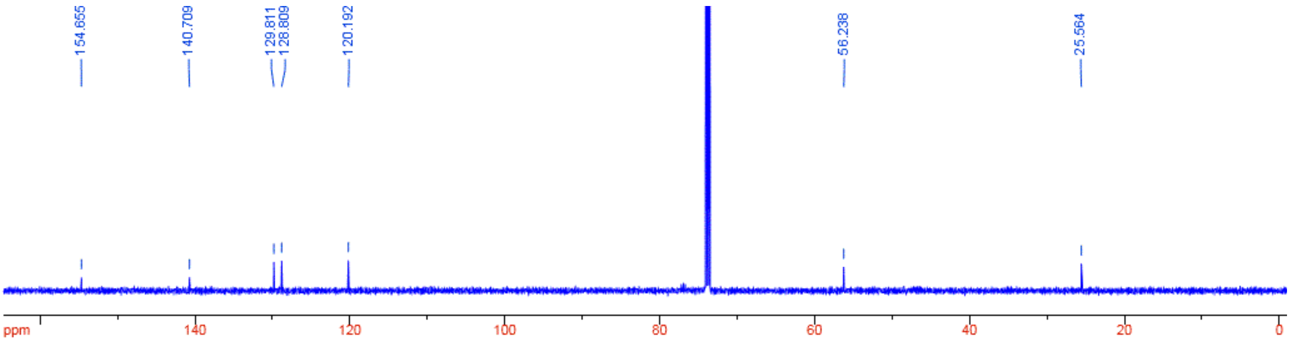

Figure S30. ^{13}C NMR spectrum of **7** (CD_2Cl_4, 80 °C)

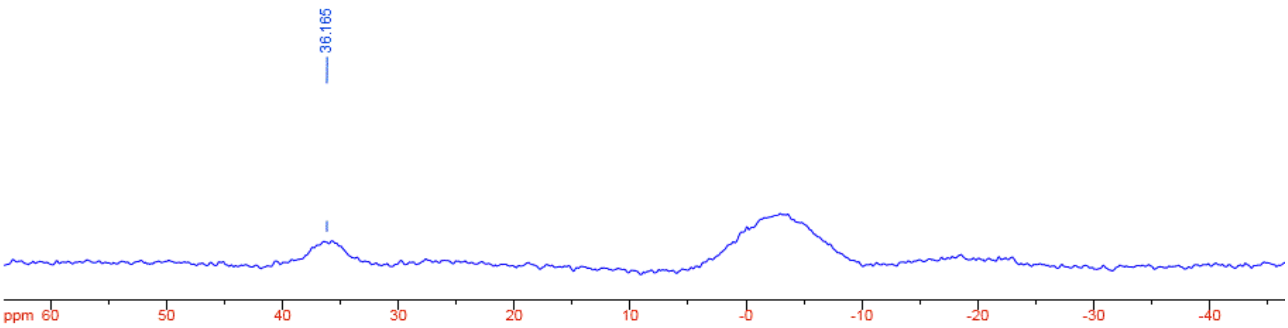

Figure S31. ^{11}B NMR spectrum of **7** (CD_2Cl_4, 75 °C)

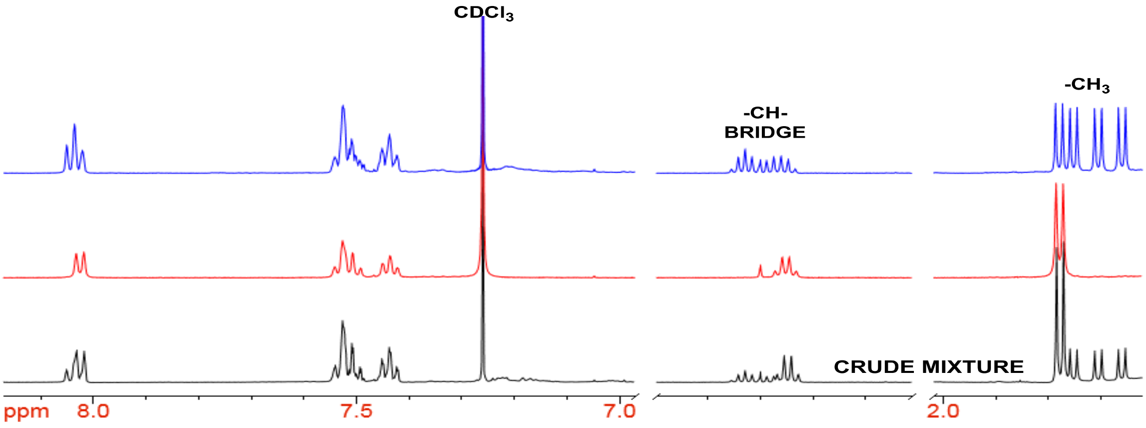

Figure S32. ^{11}H NMR spectra comparison of **9** (black: crude mixture, red: syn enantiomers, blue: 3:1 mixture of *anti* and *syn* enantiomeric pairs, $CDCl_3$, 21 °C)

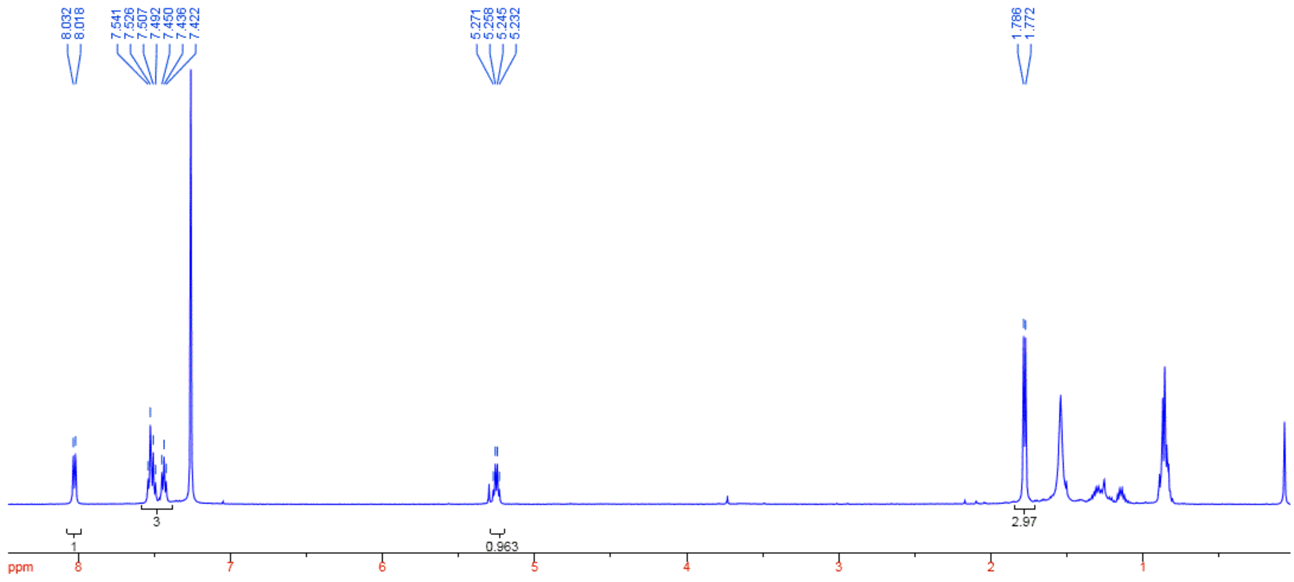

Figure S33. ^{1}H NMR spectrum of *syn*-**9** ($CDCl_3$, 21 °C)

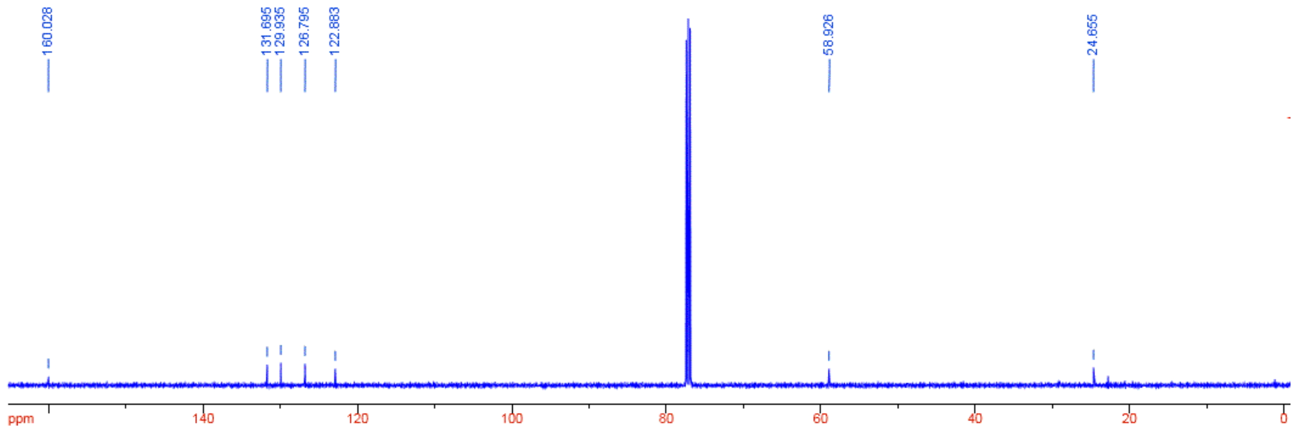

Figure S34. ^{13}C NMR spectrum of *syn*-**9** ($CDCl_3$, 21 °C)

Infrared data for borazatruxenes

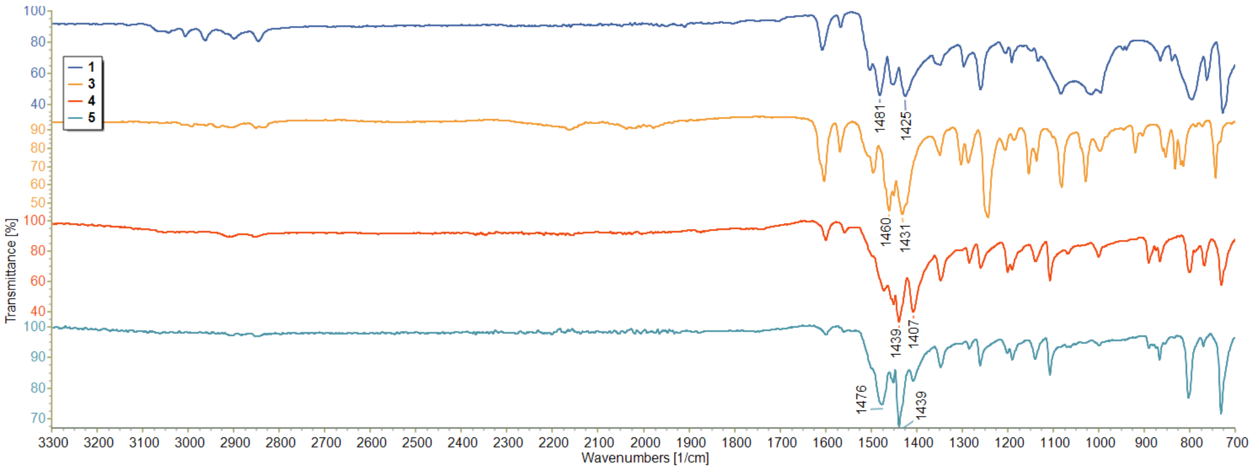

Figure S35. Stacked IR spectra of borazatruxenes **1**, **3**, **4** & **5**.

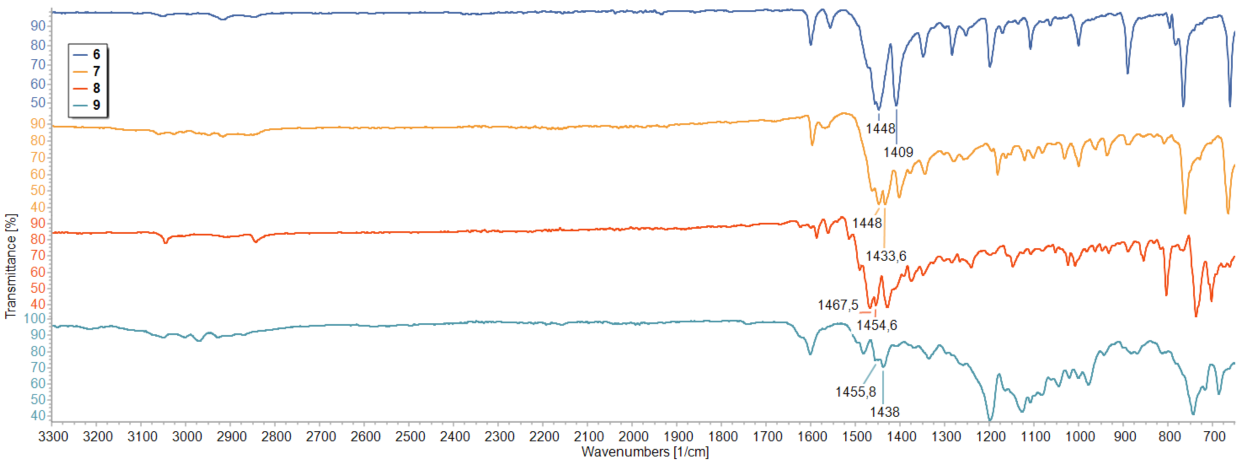

Figure S36. Stacked IR spectra of borazatruxenes **6**, **7**, **8** & **9**.

References

[1] M. P. Groziak, L. Chen, L. Yi, P. D. Robinson, *J. Am. Chem. Soc.* **1997**, *119*, 7817–7826.

[2] M. Lysén, H. M. Hansen, M. Begtrup, J. L. Kristensen, *J. Org. Chem.* **2006**, *71*, 2518–2520.

[3] J. C. Catlin, H. R. Snyder, *J. Org. Chem.* **1969**, *34*, 1664–1668.

[4] C. Rochais, R. Yougnia, T. Cailly, J. Sopková-de Oliveira Santos, S. Rault, P. Dallemagne, *Tetrahedron* **2011**, *67*, 5806–5810.

[5] P. Schmidt, C. Stress, D. Gillingham, *Chem Sci* **2015**, *6*, 3329–3333.

[6] Gaussian 16, Revision A.03, M. J. Frisch, G. W. Trucks, H. B. Schlegel, G. E. Scuseria, M. A. Robb, J. R. Cheeseman, G. Scalmani, V. Barone, G. A. Petersson, H. Nakatsuji, X. Li, M. Caricato, A. V. Marenich, J. Bloino, B. G. Janesko, R. Gomperts, B. Mennucci, H. P. Hratchian, J. V. Ortiz, A. F. Izmaylov, J. L. Sonnenberg, D. Williams-Young, F. Ding, F. Lipparini, F. Egidi, J. Goings, B. Peng, A. Petrone, T. Henderson, D. Ranasinghe, V. G. Zakrzewski, J. Gao, N. Rega, G. Zheng, W. Liang, M. Hada, M. Ehara, K. Toyota, R. Fukuda, J. Hasegawa, M. Ishida, T. Nakajima, Y. Honda, O. Kitao, H. Nakai, T. Vreven, K. Throssell, J. A. Montgomery, Jr., J. E. Peralta, F. Ogliaro, M. J. Bearpark, J. J. Heyd, E. N. Brothers, K. N. Kudin, V. N. Staroverov, T. A. Keith, R. Kobayashi, J. Normand, K. Raghavachari, A. P. Rendell, J. C. Burant, S. S. Iyengar, J. Tomasi, M. Cossi, J. M. Millam, M. Klene, C. Adamo, R. Cammi, J. W. Ochterski, R. L. Martin, K. Morokuma, O. Farkas, J. B. Foresman, and D. J. Fox, Gaussian, Inc., Wallingford CT, 2016.

[7] R. Herges, D. Geuenich, *J. Phys. Chem. A*, **2001**, *105*, 3214-3220
